# Reading and erasing of the phosphonium analogue of trimethyllysine by epigenetic proteins

**DOI:** 10.1038/s42004-022-00640-4

**Published:** 2022-03-07

**Authors:** Roman Belle, Jos J. A. G. Kamps, Jordi Poater, Kiran Kumar, Bas J. G. E. Pieters, Eidarus Salah, Timothy D. W. Claridge, Robert S. Paton, F. Matthias Bickelhaupt, Akane Kawamura, Christopher J. Schofield, Jasmin Mecinović

**Affiliations:** 1 Department of Chemistry and the Ineos Oxford Institute for Antimicrobial Research, Chemistry Research Laboratory, University of Oxford, 12 Mansfield Road, Oxford OX1 3TA, UK; 2 Chemistry−School of Natural and Environmental Sciences, Newcastle University, Newcastle upon Tyne NE1 7RU, UK; 3 Institute for Molecules and Materials, Radboud University, Heyendaalseweg 135, 6525 AJ Nijmegen, The Netherlands; 4 ICREA, Pg. Lluís Companys 23, 08010 Barcelona, Spain; 5 Departament de Química Inorgànica i Orgànica & IQTCUB, Universitat de Barcelona, Martí i Franquès 1-11, 08028 Barcelona, Spain; 6 Department of Theoretical Chemistry, Amsterdam Center for Multiscale Modeling, Vrije Universiteit Amsterdam, De Boelelaan 1083, 1081 HV Amsterdam, The Netherlands; 7 Department of Physics, Chemistry and Pharmacy, University of Southern Denmark, Campusvej 55, 5230 Odense, Denmark

## Abstract

*N*^ε^-Methylation of lysine residues in histones plays an essential role in the regulation of eukaryotic transcription. The ‘highest’ methylation mark, *N*^ε^-trimethyllysine, is specifically recognised by *N*^ε^-trimethyllysine binding ‘reader’ domains, and undergoes demethylation, as catalysed by 2-oxoglutarate dependent JmjC oxygenases. We report studies on the recognition of the closest positively charged *N*^ε^-trimethyllysine analogue, i.e. its trimethylphosphonium derivative (K_P_me_3_), by *N*^ε^-trimethyllysine histone binding proteins and *N^ε^-*trimethyllysine demethylases. Calorimetric and computational studies with histone binding proteins reveal that H3K_P_4me_3_ binds more tightly than the natural H3K4me_3_ substrate, though the relative differences in binding affinity vary. Studies with JmjC demethylases show that some, but not all, of them can accept the phosphonium analogue of their natural substrates and that the methylation state selectivity can be changed by substitution of nitrogen for phosphorus. The combined results reveal that very subtle changes, *e.g*. substitution of nitrogen for phosphorus, can substantially affect interactions between ligand and reader domains / demethylases, knowledge that we hope will inspire the development of highly selective small molecules modulating their activity.

Eukaryotic histones are subject to numerous posttranslational modifications (PTMs) that regulate expression in a context dependent manner. Histone lysine-residues are amongst the most frequently modified of all residues, including by acylation-type modifications, most commonly acetylation^1^. They are also iteratively *N*^ε^-methylated to give *N*^ε^-monomethylated (Kme_1_), *N*^ε^-dimethylated (Kme_2_), and *N*^ε^-trimethylated lysine (Kme_3_) residues (Fig. 1a). The roles of lysine *N*^ε^-methylation depend on factors including methylation state and location in nucleosomal complexes. Typically, histone H3 *N*^ε^-trimethyllysine 4 (H3K4me_3_), H3K36me_3_, and H3K79me_3_ are linked to transcriptional activation, while H3K9me_3_, H3K27me_3_ and H4K20me_3_ are linked to suppression^2^. *N*^ε^-Methyllysine groups are installed by histone *N*^ε^-lysine methyltransferases (KMTs, “writers”) and removed by histone *N*^ε^-lysine demethylases (KDMs, “erasers”) (Fig. 1b). *N*^ε^-Methyllysine chromatin binding proteins (“readers”) bind to specific *N*^ε^-methylated lysines to enable gene regulation^3,4^. KDMs have either an amine-oxidase or, more commonly, a JumonjiC (JmjC) catalytic domain^4^. The JmjC KDMs are Fe(II) and 2-oxoglutarate (2OG) dependent dioxygenases, which normally couple two electron substrate oxidation, e.g., *N*^ε^-methyllysine demethylation, to conversion of 2OG/O_2_ to succinate/CO_2_. JmjC KDMs catalyse removal of mono-, di-, or trimethyl groups *via* hydroxylation of an *N*^ε^-methyl group followed by decomposition of the hemiaminal intermediate producing formaldehyde and the demethylated product^4^.

The interplay between KMTs and KDMs regulates lysine methylation status, which in turn regulates binding of methylation state-specific chromatin binding modules. Four identified non-catalytic domains interact with *N*^ε^-trimethyllysines: plant homeodomains (PHD) (Fig. 1c), tandem tudor domains (TTD), chromodomains (CHD), and malignant brain tumour (MBT) proteins^3^, all of which bind *N*^ε^-trimethyllysine in a cage comprised of typically hydrophobic and aromatic residues^4^. Experimental and computational studies have shown that binding of *N^ε^*-trimethyllysine by readers is driven by cation-π interactions between the positively charged quaternary ammonium group of *N*^ε^-trimethyllysine and electron-rich aromatic residues and by release of water molecules from the cage^5–9^.

Misregulation of histone modification is linked to human disease. For example, DNA encoding for the PHD3 finger of the KDM5A demethylase can fuse with that of nuclear pore protein 98 (NUP98) leading to the NUP98-KDM5A-PHD3 fusion protein, which is linked to acute myeloid leukaemia^10^. BPTF, a core subunit of the ATP-dependent nucleosome remodelling factor (NURF), can fuse with NUP98 to result in primary refractory acute megakaryoblastic leukaemia protein^11^. Other readers forming NUP98 fusion proteins include PHF23, NSD1, and NSD3^12,13^.

Research on KMTs and KDMs^4^ has led to potent and partially selective inhibitors, of use in studying their roles and therapeutic potential. However, there remain challenges in achieving selectivity for particular KDM isoforms (there are >25 JmjC oxygenases and >60 2OG oxygenases in humans^14,15^). Development of selective inhibitors for chromatin binding modules, which are often present in epigenetic writers and erasers, has been particularly challenging, with reported inhibitors of the >100 human PHD^16^ and TTD^17^ domains being relatively weak and non-selective binders^18–20^. Knowledge of the selectivity of ligand binding by chromatin binding proteins and modifying enzymes is thus of both fundamental and medicinal interest^21^. To investigate the extent to which histone *N*^ε^-methyllysine readers and erasers can manifest selectivity, we synthesised a peptide containing the simplest possible positively charged *N*^ε^-trimethyllysine analogue, i.e., ^ε^-trimethylphosphonium lysine (K_P_me_3_), and studied its interactions with histone *N*^ε^-methyllysine readers and erasers (Fig. 1). The results reveal that, at least some, readers and erasers can discriminate between K_P_me_3_ and Kme_3_ peptides, suggesting that identification of drug-like selective inhibitors should be possible.

## Results

To study the effect of nitrogen substitution of *N*^ε^-trimethyllysine to phosphorus under in vitro conditions, the Fmoc-protected *P*^ε^-trimethylphosphonium analogue of *N*^ε^-trimethyllysine (Fmoc-K_P_me_3_-OH) was synthesised from L-lysine in nine steps (Supplementary Scheme 1). The Fmoc-K_P_me_3_-OH and the Fmoc-Kme_3_-OH control were incorporated into human histone H3-tail fragment peptides (histone H3 residues 1–10, ART(K_P_me_3_)QTARKS: H3K_P_4me_3_/ART(Kme_3_)QTARKS: H3K4me_3_; and histone H3 residues 1–15, ARTKQTAR(K_P_me_3_)STGGKA: H3K_P_9me_3_/ARTKQTAR(Kme3) STGGKA: H3K9me_3_) using Fmoc mediated solid-phase peptide synthesis (SPPS), followed by preparative HPLC (Supplementary Scheme 1).

### ITC analysis of H3K4me_3_ and H3K_P_4me_3_ with reader domains

**Fig. 2:**
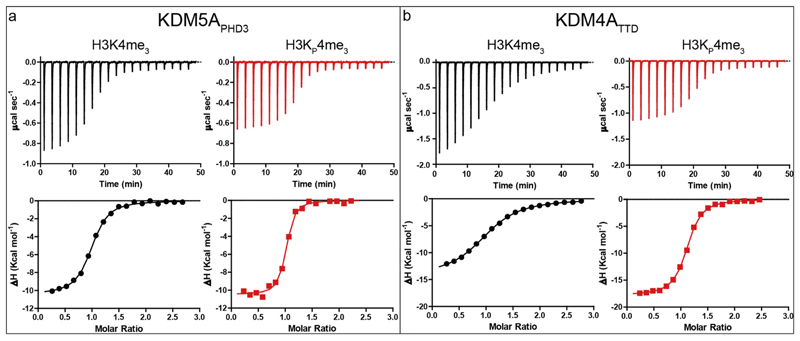
Thermodynamic analyses of binding. Representative ITC results from the interaction of (**a**) KDM5A_PHD3_ and (**b**) KDM4A_TTD_ with H3K4me_3_ (black) or H3K_P_4me_3_ (red) substrates. Top panels show the raw ITC data and the bottom panels show the processed results.

We investigated the thermodynamics of association of the H3K_P_4me_3_ peptide with five representative human reader domains containing either a PHD zinc finger or TTD, i.e. KDM5A_PHD3_ (JARID1A-PHD3, residues M1489–V1641)^10^, TAF3_PHD_ (R857–K924)^22^, BPTF_PHD_ (L2583–N2751)^23^, SGF29_TTD_ (R115–K293)^24^ and KDM4A_TTD_ (JMJD2A, Q897-P1011)^25^, selected on the basis of their preference for binding *N*^ε^-trimethyllysine over other (non)methylation marks (Kme_0_ < Kme_1_ < Kme_2_ < Kme_3_), and their domain and aromatic cage diversity. The recombinant readers were produced in *E. coli* following reported procedures^26^. Isothermal titration calorimetric (ITC) analyses were used to determine the dissociation constant (*K*_d_), the Gibbs free energy of binding (Δ *G*°), the enthalpy of binding (Δ*H*°), and the entropy of binding (Δ*S*°). Results with the H3K4me_3_ control peptide correlated with reported values^10,23–25,27^ (Table 1, Fig. 2, Supplementary Fig. 1).

Interestingly, for four of the readers, ITC experiments with H3K_P_4me_3_ indicated stronger complex formation than with H3K4me_3_ (Table 1). The largest increase was observed with KDM4A_TTD_, which manifested ~12-fold stronger binding with H3K_P_4me_3_ compared to H3K4me_3_. SGF29_TTD_ exhibits comparable binding affinity for H3K4me_3_ and H3K_P_4me_3_; note that it is the only reader tested not possessing a Trp residue in its hydrophobic cage^24^. This result correlates with the observed unusually strong binding of the neutral *N*^ε^-trimethyllysine carbon-analogue to SGF29_TTD_ compared with other reader proteins^5^. The increase in affinity for H3K_P_4me_3_ relative to H3K4me_3_ is generally a result of a more favourable Δ*H*°, with the values for the Δ*S*° remaining largely unchanged for four of the five readers, the exception being KDM4A_TTD_, (Table 1). Although the observed decreases in Δ*H*° are small (ΔΔ*H*°: − 0.4 to −1.3 kcal mol^−1^) for KDM5A_PHD3_, TAF3_PHD_, BPTF_PHD_ and SGF29_TTD_, the decrease for H3K_P_4me_3_ relative to H3K4me_3_ is relatively large (ΔΔ*H*°: −4.7 kcal mol^−1^) for KDM4A_TTD_. The more favourable Δ*H*° for binding for H3K_P_4me_3_ over H3K4me_3_ implies more favourable cation-π interactions between the trimethylphosphonium cation and the electron-rich aromatic cages, as found in related systems^5,7,8^. The longer C-P bond (1.87 Å) in H3K_P_4me_3_ compared to H3K4me_3_ (C-N bond in H3K4me_3_ is 1.47 Å) and increased volume (^+^Pme_4_: 115 Å^3^, ^+^Nme_4_: 105 Å^3^)^28^ may help position the methyl hydrogens of the quaternary phosphonium cation closer to the aromatic cage residues. Note that, the limited added volume of H3K_P_4me_3_ compared to H3K4me_3_ means both are likely to release the same number of water molecules from the cages, suggesting equal contributions to affinity due to reader desolvation. Overall, the ITC results imply that the readers efficiently recognise the phosphonium analogue of *N*^ε^-trimethyllysine: importantly, despite the subtle nature of the difference between H3K_P_4me_3_ compared to H3K4me_3_, differences in the relative binding efficiencies of the readers for the peptides were observed.

### Molecular dynamics simulations of histones with readers

**Fig. 3:**
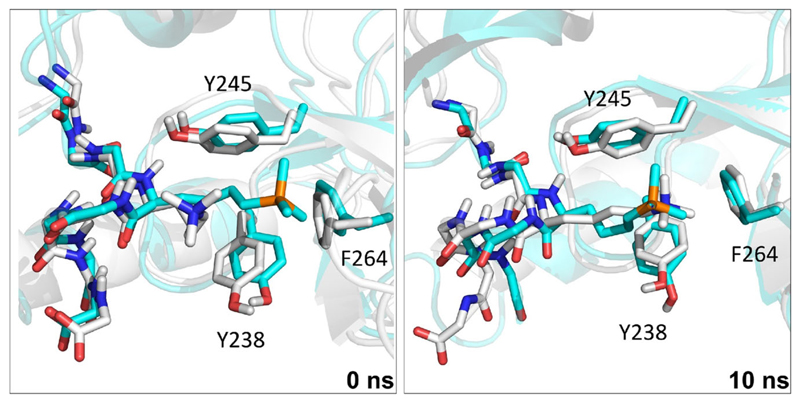
MD simulation studies for reader SGF29_TTD_. Snapshots of simulations for SGF29_TTD_ complexed with a histone H3 fragment (liquorice) containing K_P_4me_3_ (cyan) or K4me_3_ (white) at 0 ns and 10 ns.

We used molecular dynamics (MD) simulations to study how the readers bind to H3K4me_3_ and H3K_P_4me_3_. The *N*^ε^-trimethyllysine residue of H3K4me_3_ in structures of reader protein complexes was replaced with K_P_me_3_ with solvation in a 10 Å truncated octahedral box of TIP3P water^29^ and neutralised explicitly with sodium or chloride ions. AMBER12^30^ was used to simulate the systems for 10 ns (Supplementary Figs. 2–7, Supplementary Tables 1 and 2)^31^. Although this timescale is not long enough to observe events such ligand binding or substantial conformational changes, such simulations have been shown to be valuable in recent studies evaluating the stability of protein–ligand complexes and identifying potential favourable or unfavourable non-covalent interactions, including for reader–H3K4me_3_ complexes^7,8,32,33^. Over the simulation time, the SGF29_TTD_–H3K_P_4me_3_ complex manifests a similar pose to the SGF29_TTD_–H3K4me_3_ complex (Fig. 3), including in the hydrophobic cage (Y238, Y245, and F264) (Supplementary Figs. 2–7). The H3K_P_4me_3_ residue mimics the binding pose of H3K4me_3_ with respect to the cage residues, except for KDM5A_PHD3_ (Supplementary Fig. 4). With KDM5A_PHD3_, H3K_P_4me_3_ showed large fluctuations in the distance to W18 of the cage, an observation apparently reflected in previous MD studies where modifications to H3K4me_3_ yield less favourable interactions with the KDM5APHD3 W18 compared with W28^7,8^.

### Quantum chemical analyses in the gas and aqueous phase.

We then analysed the energetics of binding for Kme_3_ and K_P_me_3_ (the side chains of H3K4me_3_ and H3K4_P_me_3_, respectively) with TRP2, a model for two aromatic cage-comprising tryptophan residues, using quantum chemical methods. This model was chosen because KDM5A_PHD3_ has only two aromatic residues present in its aromatic cage (W1625 and W1635, Supplementary Figs. 4 and 5). Such a simple model cannot respect the dynamics of complex protein-protein interactions; however, the results are informative with respect to the interactions of Kme_3_ and K_P_me_3_ side chains with KDM5A_PHD3_^5,7,8^. We used dispersion-corrected density functional theory (DFT) employing BLYP-D3BJ/TZ2P and COSMO for simulating aqueous solutions with ADF. The model complex TRP2-K_P_me_3_ presents a 1.2 kcal mol^–1^ stronger bonding interaction than TRP2-Kme_3_, with a Δ*E*(aq): -11.4 and -10.2 kcal mol^–1^ for the K_P_me_3_ and Kme_3_ complexes, respectively (Table 2). The Kme_3_ and K_P_me_3_ side chains in the modelled complexes have similar conformations, despite the larger size of P (Table 2 and Supplementary Fig. 8). The models imply that both the Kme_3_ and K_P_me_3_ side chains undergo only small deformations on TRP2 binding, as reflected in the strain energies: Δ*E*(aq)_strain_: 0.1 and 0.8 kcal mol^–1^ for the Kme_3_ and K_P_me_3_ complexes, respectively. The preference for K_P_me_3_ over Kme_3_ is also manifested in the absence of water, although to a lesser extent: Δ*E*_int_: –27.6 and –28.0 kcal mol^–1^ for the Kme_3_ and K_P_me_3_ complexes, respectively. This result supports the above proposal that, energetically, desolvation effects (Δ*E*(desolv)_int_) are similar for Kme_3_ and K_P_me_3_.

**Fig. 4:**
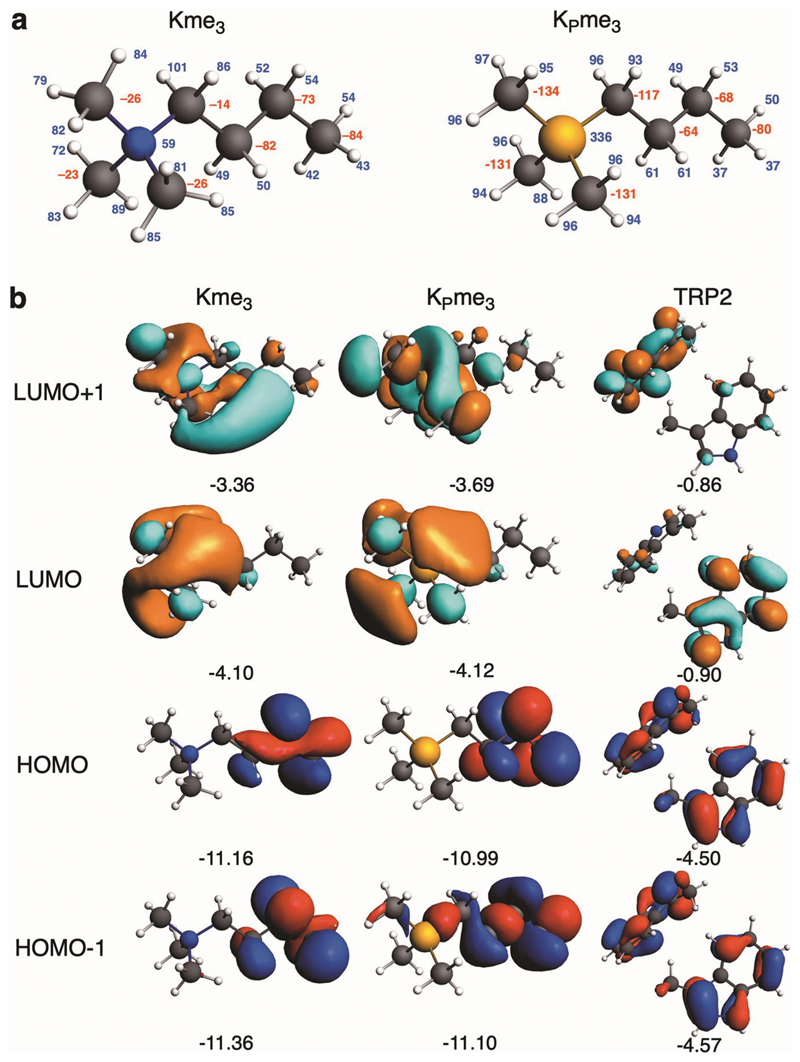
Analysis of Kme_3_ and K_P_me_3_ interactions with TRP2, a model for the KDM5A_PHD3_ reader. The TRP2 model employs the two tryptophan residues found in KDM5A_PHD3_ (W1625, W1635). **a** Calculated VDD atomic charges (in mili-a.u.) for H3K4me_3_ and H3K_P_4me_3_ (red: negative, blue: positive). **b** Frontier orbitals (with orbital energies in eV) for Kme_3_, K_P_me_3_, and TRP2, (isosurface drawn at 0.03), computed at the BLYP-D3BJ/TZ2P level using an X-ray structure for TRP2 (PDB: 3GL6).

We investigated why the TRP2 aromatic cage interacts more favourably with K_P_me_3_ than Kme_3_, using quantitative Kohn-Sham molecular orbital (KS-MO) theory and energy decomposition analysis (EDA) of Δ*E*_int_ (Table 2). The results imply that the more stabilizing interaction Δ*E*_int_ for H3K_P_4me_3_ originates from more attractive electrostatic (by 0.8 kcal mol^–1^), orbital (by 0.6 kcal mol^–1^), and dispersion (by 1.6 kcal mol^–1^) interactions. The stronger electrostatic attraction of K_P_me_3_ is due to the somewhat more positively charged methyl H atoms of the phosphonium group (Fig. 4a). The more attractive Δ*E*_oi_ term in TRP2–K_P_me_3_ results from stronger, more stabilizing donor-acceptor orbital interactions from π orbitals to the σ*_C-H_ type orbitals on the K_P_me_3_ side chain: the charge transfer is 0.09 electrons to K_P_me_3_ and only 0.04 electrons to Kme_3_ (Fig. 4b). The preference for K_P_me_3_ is caused by the lower energy of the σ*_C-H_ type orbitals of K_P_me_3_ and their better overlap with π orbitals (Supplementary Table 3). Our bonding analyses show that these cation-π interactions can be viewed as cationic CH-π interactions. Note that the more favourable bonding terms in the TRP2-K_P_me_3_ complex leads to a shorter d(H_Me_-C_TRP-5MR_) distance between the phosphonium group and the cage, which slightly amplifies all interaction terms, including the steric (Pauli) repulsion (by 2.7 kcal mol^–1^, Table 2).

We carried out EDA analyses for the homolytic formation of the C-H bonds in Kme_3_ and K_P_me_3_ at the BLYP-D3BJ/TZ2P level (Supplementary Table 4 and Supplementary Fig. 9). The larger proton affinity for Kme_3_ compared to K_P_me_3_ is maintained both in solution and in the gas phase. The EDA results imply that this derives substantially from the more favourable electrostatic interactions for Kme_3_ compared to K_P_me_3_ (by 5.4 kcal mol^–1^), even though the orbital interactions are more favourable for K_P_me_3_, though only by 1.2 kcal mol^–1^. The VDD charge on CMe is -268 me and on N is +42 me, whereas for K_P_me_3_ the VDD charge for CMe is -348 me and +302 me on P. The difference in homolytic formation of C-H bonds for Kme_3_ or K_P_me_3_ thus seems to be a subtle interplay of a more favourable (i.e. more negative) charge on the methyl carbon plus a less favourable (more positive) charge on the P atom in K_P_me_3_.

### MS and NMR studies show KDM4E_JmjC_ can demethylate H3K_P_9me_3_

**Fig. 5:**
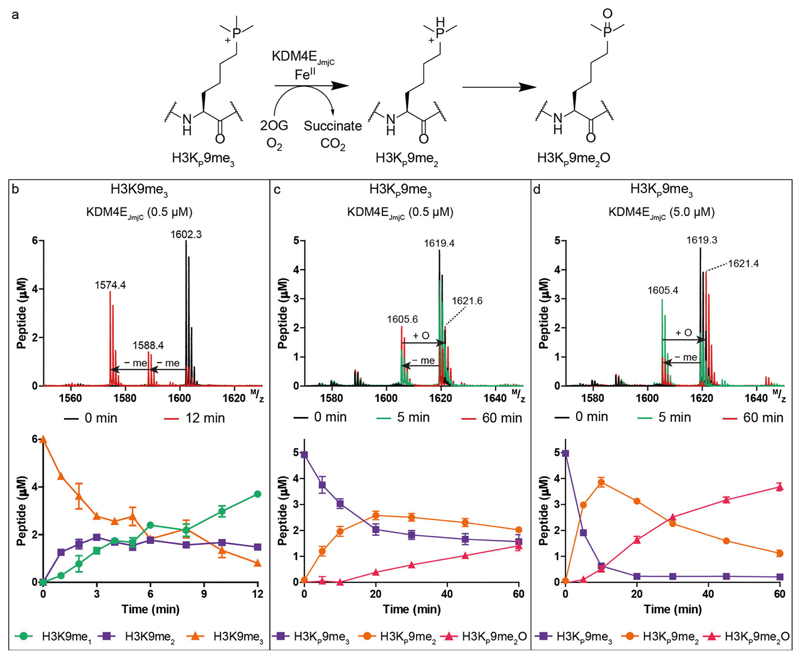
KDM4E_JmjC_ catalyses demethylation of H3K_P_9me_3_ to give H3K_P_9me_2_. **a** KDM4E_JmjC_ catalysed demethylation of H3K_P_9me_3_. **b** Mass spectra and time-course analysis of H3K9me_3_ (6.0 μM) and KDM4E_JmjC_ at 0 min (black) and 12 min (red) showing the substrate H3K9me_3_ (orange) and demethylated products H3K9me_2_ (purple) and H3K9me_1_ (green). Mass spectra and time-course analysis of H3K_P_9me_3_ (5.0 μM) and KDM4E_JmjC_ (**c** 0.5 μM, **d** 5.0 μM) at time points 0 (red), 5 (green) and 60 min (red) acquired using MALDI-TOF MS. Conditions: Asc (500 μM), Fe(II) (50 μM) and 2OG (100 μM). Errors represent standard deviations (*n* = 2 or 3).

Having demonstrated that H3K_P_4me_3_ is a stronger binder than H3K4me_3_ with most of the readers, we investigated whether JmjC KDMs can catalyse demethylation of H3K_P_9me_3_, as occurs for H3K9me_3_ (Fig. 5a). We chose human KDM4E_JmjC_ (M1–Q337), a histone H3K9me_3/2_ demethylase with relatively high demethylation activity as a model enzyme^34^. Reactions were monitored using MALDI-TOF mass spectrometry (Fig. 5b–d). KDM4E_JmjC_ (0.5 μM) efficiently catalysed the di-demethylation of the positive control H3K9me_3_ (6.0 μM) as indicated by two -14 Da mass shifts, as anticipated (Fig. 5b)^34^. Michaelis-Menten kinetics yielded a *K_M_* of 6.1 μM and k_cat_ of 5.3 min^–1^ (V_max_: 2.7 μM·min^–1^) (Supplementary Fig. 10), similar values to those reported using the shorter H3_7–14_K9me_3_ substrate (*K*_M_: 21.3 μM and *k*_cat_: 4.6 min^–1^)^35^.

When H3K_P_9me_3_ (5.0 μM) was treated with KDM4E_JmjC_ (0.5, 5.0 μM) (Fig. 5b, c), H3K_P_9me_3_ was consistently observed to only undergo a single demethylation (–14 Da) to give H3K_P_9me_2_. Masses corresponding to potential subsequent demethylation to give H3K_P_9me_1_ or H3KP9 were not detected. Along with formation of H3K_P_9me_2_, time-dependent production of another product, assigned as the phosphine oxide (H3K_P_9me_2_O) (+16 Da), was observed (Fig. 5c, d). Reaction of stoichiometric amounts of KDM4EJ_m_j_C_ (5.0 μM) and H3K_P_9me_3_ (5.0 μM) showed faster H3K_P_9me_2_ and H3K_P_9me_2_O product formation, but no evidence for H3K_P_9me_1_ or H3K_P_9 formation. Controls demonstrated little or no H3K_P_9me_2_ or H3K_P_9me_2_O formation without KDM4EJ_m_j_C_, ascorbate (Asc), Fe(II) or 2OG (Supplementary Fig. 11). By contrast, with H3K9me_3_ (which is a better substrate than H3K_P_9me_3_ – see below), without Asc and Fe(II) some demethylation was observed, likely reflecting co-purifying Fe(II) and 2OG (Supplementary Fig. 12). With Tris(2-carboxyethyl)phosphine (TCEP) as a reducing agent, rather than Asc, slightly increased yields of H3K_P_9me_2_ and H3K_P_9me_2_O were observed (Supplementary Fig. 13). Addition of catalase (to supress hydrogen peroxide formation^36^) with or without Asc or BSA did not substantially alter the amounts of H3K_P_9me_2_ or H3K_P_9me_2_O (Supplementary Fig. 13). To examine further whether reaction of H3K_P_9me_2_ to H3K_P_9me_2_O occurs enzymatically and / or non-enzymatically, reactions were quenched (H3K9me_3_: 5 min or H3K_P_9me_3_: 10 min) with formic acid, EDTA or 2,4-PDCA, incubated, then quenched again (H3K9me_3_: 30 min or H3K_P_9me_3_: 60 min) with formic acid (Supplementary Fig. 14). The H3K9me_3_ results show little variations in product profiles indicating that the reagents are experimentally effective. With H3K_P_9me_3_ where H3K_P_9me_2_ and H3K_P_9me_2_O are produced, on initial quenching with EDTA or 2,4-PDCA (which inhibits by chelating to Fe) we observed a slow increase in the peak corresponding to H3K_P_9me_2_O, but not H3K_P_9me_2_. The combined observations imply that slow production of H3K_P_9me_2_O from H3K_P_9me_2_ can occur via non-enzymatic as well as enzymatic oxidation. To verify that products detected using MALDI-TOF MS are not instrumental artefacts, time-course measurements were performed on H3K9me_3_ and H3K_P_9me_3_ with analysis by LC-MS. Similar demethylation and oxidation patterns, including production of H3K_P_9me_2_ and H3K_P_9me_2_O from H3K_P_9me_3_ were detected as observed with MALDI-TOF MS (Supplementary Fig. 15).

To directly compare the efficiency of demethylation of H3K9me_3_ (5.0 μM) and H3K_P_9me_3_ (5.0 μM), they were incubated with KDM4EJ_m_j_C_ (0.5 μM) in the same vessel (Supplementary Fig. 16). H3K9me_3_ was converted to H3K9me_2/1_ with the same efficiency as the control, but there was no evidence that H3K_P_9me_3_ was converted to H3K_P_9me_2_ or H3K_P_9me_2_O, although low levels of formation of H3K_P_9me_2_ cannot be ruled out as its peak overlaps with an isotope peak of H3K9me_3_. The combined results show H3K9me_3_ is a substantially better substrate than H3K_P_9me_3_.

**Fig. 6:**
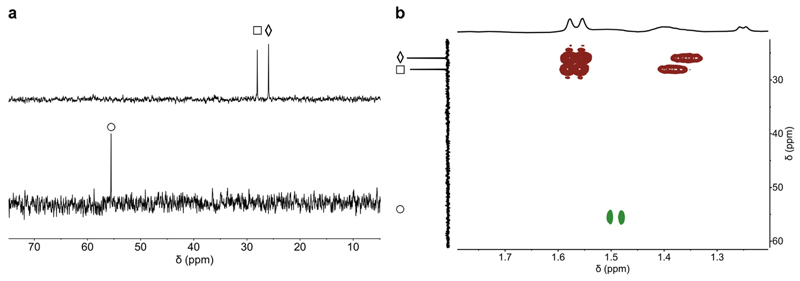
KDM4E_JmjC_ catalyses demethylation of H3K_P_9me_3_ to give H3K_P_9me_2_H and H3K_P_9me_2_O as monitored by ^31^P NMR and ^1^H-^3^P HMBC. **a** ^31^P NMR analyses of H3K_P_9me_3_ (◊) incubated with KDM4E, addition of acid (top) or quenched by heating (bottom). Evidence for formation of H3K_P_9me_2_H (□, δ_P_ = 28.4 ppm, top) and H3K_P_9me_2_O (○, δ_P_ = 55.6 ppm, bottom). **b** Overlay of ^1^H-^31^P HMBC analyses of the solutions after of H3K_P_9me_3_ incubated with KDM4E_Jm_j_C_, quenched by heating (green cross peaks), or addition of acid (red cross peaks). Experimental conditions: H3K_P_9me_3_ (500 μM), Asc (1.00 mM), 2OG (1.00 mM), Fe(II) (100 μM), KDM4E_JmjC_ (50 μM).

KDM4E_JmjC_-catalysed demethylation of H3K_P_9me_3_ was analysed by ^1^H and ^31^P NMR; in both cases, the reaction proceeded to give signals corresponding to H3K_P_9me_2_ and H3K_P_9me_2_O (Fig. 6). In the ^31^P NMR, distinct resonances were observed for H3K_P_9me_3_ (25.9 ppm), H3K_P_9me_2_H (28.4 ppm), and H3K_P_9me_2_O (55.6 ppm). Notably, a different ^31^P resonance (δ_P_: 28.4 ppm compared to 55.6 ppm) was observed when quenching the H3K_P_9me_3_ reaction with HCl (1 M); this was assigned as a protonated H3K_P_9me_2_H species, as supported by ^1^H-^31^P HMBC NMR, and comparison of chemical shifts with those for similar species, i.e. PMe_3_, PMe_3_H^+^, and P(O)Me_3_ (Supplementary Fig. 17), under identical conditions. ^31^P and ^1^H NMR time-course studies confirmed demethylation and conversion of 2OG to succinate (Supplementary Fig. 18).

To test whether formaldehyde is generated by H3K_P_9me_3_ demethylation, a formaldehyde dehydrogenase (FDH) coupled assay was employed (Supplementary Fig. 19a)^34,37^. With H3K9me_3_, this assay gave *K_M_:* 5.1 μM and *k_cat_:* 6.1 min^–1^ (*V*_max_: 0.61 μM·min^–1^) (Supplementary Fig. 19b,c), values comparable to those obtained by MALDI-TOF MS. Measurements using H3K_P_9me_3_ also show an KDM4E_JmjC_ and time-dependent increase in formaldehyde (Supplementary Fig. 20), but the low activity prohibited detailed kinetics analysis of H3K_P_9me_3_ using the FDH-assay.

### Demethylation of H3K_P_9me_3_ by other KDM4s

To investigate whether H3K_P_9me_3_ can be demethylated by other human KDM4 subfamily members, recombinant KDM4A_JmjC_ (M1–L359) and KDM4D_JmjC_ (M1–L358) were produced in *E. coli* following an adaption of literature procedures^38,39^. At a relatively high enzyme concentration, KDM4A_JmjC_ (2.4 μM) and KDM4D_JmjC_ (2.4 μM) demonstrate clear demethylation activity on H3K9me_3_ (5.0 μM) (Supplementary Figs. 21 and 22). With H3K_P_9me_3_ (5.0 μM) substantial turnover to H3K_P_9me_2_ was observed with KDM4A_JmjC_ and KDM4D_JmjC_, with little H3K_P_9me_2_O formation being observed. No evidence for further demethylation was accrued. Unlike the KDM4s, KDM3A/B (JMJD1A/B) and KDM7A/B (PHF8) do not catalyse demethylation of H3K9me_3_, but demethylate H3K9me_2/1_ to give the unmethylated lysine residue. To test the ability of KDM3 and KDM7 subfamily representatives to demethylate H3K_P_9me_2_ or H3K_P_9me_3_, KDM3A_JmjC_ (T515–S1317) and KDM7B_JmjC_ (M37–N483) were produced using baculovirus/sf9 and E. coli expression systems, respectively^40,41^. The synthesis of a histone H3 mimic peptide H3K_P_9me_2_ substrate is challenging as the tri-alkylated phosphine group is susceptible to oxidation during synthesis of the protected amino acid and during SPPS requiring oxygen-free conditions. Thus, to investigate if KDM3A_JmjC_ or KDM7B_JmjC_ can catalyse demethylation of H3K_P_9me_2_, an appropriate H3K_P_9me_2_ substrate was prepared in situ from H3K_P_9me_3_ using KDM4E_JmjC_ (2.0 μM), in the presence of KDM3A_JmjC_ (2.0 μM) or KDM7B_JmjC_ (2.0 μM). [Note, KDM7BJmjC exhibits significantly higher H3K9me2 demethylation rates with trimethylated lysine 4 (H3K4me_0_ < H3K4me_3_), but is also active without the H3K4me_3_ modification^40^]. The results show that KDM3A_JmjC_ and KDM7B_JmjC_ demethylate their ‘natural’ H3K9me_2_ substrate^40,42^, but do not catalyse demethylation of H3K9me_3_ (as anticipated) or H3K_P_9me_3_ (Supplementary Fig. 23a–f). Unlike KDM4E_JmjC_ alone, the combination of KDM4E_JmjC_ with KDM3AJmjC or KDM7BJmjC and H3K9me_3_ manifests conversion to H3K9me_1_. The same combinations but with H3K_P_9me_3_, produced H3K_P_9me_2_ (due to KDM4E_JmjC_ catalysis), but did not result in masses consistent with H3K_P_9me_1_ or H3K_P_9, implying that H3K_P_9me_2_ is not a substrate for KDM3AJmjC or KDM7BJmjC (Supplementary Fig. 23h, j, l).

## Discussion

Methylation of carbon, nitrogen, oxygen and sulphur atoms in large and small biomolecules is of central biological importance; methyl groups linked via heteroatoms are common in drugs and agrochemicals. Alkylated phosphines are commonly used in organic synthesis, *e.g*., in Wittig reagents. It is thus perhaps surprising that methylphosphonium and related chemistry has, to our knowledge, not been more widely investigated in biochemistry^43,44^, in particular with respect to the possibility of demethylation.

Our studies on interactions between H3K4me_3_ and H3K_P_4me_3_ and readers demonstrate that H3K_P_4me_3_ can substitute for H3K4me_3_^45^, in most cases with increased affinity, due to stronger cation-π interactions (bonding analyses reveal true cationic CH-π interactions). Notably, there are differences in the relative binding efficiencies of the readers with H3K4me_3_ compared to H3K_P_4me_3_, implying selective inhibition of readers by small drug-like molecules should be feasible. Similar observations have been made in relation to cation-π interactions between tetramethylammonium compounds and their tetramethylphosphonium analogues with respect to binding to aromatic cavities. Related studies with γ-butyrobetaine^43^ and the serine protease factor Xa^44^ suggest our observations may be of a general nature.

The results with H3K_P_4me_3_ contrast those for other H3K4me_3_ derivatives binding to readers, where typically comparable or lower affinities are observed (Supplementary Fig. 24, Supplementary Table 5) compared to H3K4me_3_. For example, studies comparing binding of H3K4me_3_ and H3KC4me_3_ to TAF3_PHD_ and KDM4A_TT_D reveal impaired binding of H3KC4me_3_(ΔK_d_ values of ~2-fold)^46^. By contrast, an increase in, or comparable, stability is observed for the H3K_P_4me_3_-reader complexes relative to the H3K4me_3_-reader complexes, with some showing much tighter binding (BPTF_PHD_, ΔK_d_: ~7-fold and KDM4A_TT_D, ΔK_d_: ~12-fold). For comparison, the difference in binding between H3K4me_3_ and unmodified-lysine is protein and condition dependent, but typically the ΔK_d_ is >20-fold in favour of H3K4me_3_^10,23–25,47,48^. Even more pronounced decreases in binding affinities are observed with KDM5A_PHD3_ and TAF3_PHD_^5^ when the K4me_3_ in H3K4me_3_ is substituted for glycine, highlighting the importance of the lysine side chain in binding. Thus, the substitution of H3K4me_3_ for H3K_P_4me_3_ can have a positive effect on binding, knowledge that might be exploited in inhibitor design.

Previous studies revealed that some JmjC KDMs can catalyse oxidation of substrates other than the established *N*^ε^-methylated lysine substrates, *e.g*., H3K9me_3/2_ for KDM4E, as demonstrated with *N*^ε^-methyl-ethyl-lysine-9, a substrate that undergoes both demethylation and deethylation^49^. However, analysis with *N*^ε^-diethyllysine showed no evidence of reaction, demonstrating limitation of the plasticity of the KDM4E active site towards alkylated lysine substrates. Some JmjC KDMs can also catalyse *N*-methyl arginine demethylation and with appropriately sized *N*^ε^-substitutions some can catalyse formation of stable alcohol products^49,50^. We found that H3K_P_9me_3_ is a demethylation substrate for human KDM4A/D/E to give H3K_P_9me_2_; this observation is consistent with the relatively small increase in volume when H3K_P_9me_3_ is compared to H3K9me_3_ (Δ[^+^Pme_4_-^+^Nme_4_]: 10 Å^3^)^28^, though the demethylation rate is significantly slower for H3K_P_9me_3_ than for H3K9me_3_. Strikingly, although KDM4 enzymes (KDM4A/D/E) catalysed formation of H3K_P_9me_2_, they did not catalyse its further demethylation to give H3K_P_9me_1_, despite efficient conversion of H3K9me_2_ to H3K9me_1_. We propose that this, at least in part, is due to the decreased pK_a_ of H3K_P_9me_2_ versus H3K9me_2_ – it seems that, at least for the KMD4 JmjC KDMs, the positively charged form of *N*^ε^-dimethyllysine H3K9me_2_ is the preferred substrate. Interestingly, we also observed conversion of H3K_P_9me_2_ to H3K_P_9me_2_O, possibly in part by non-enzymatic oxidation; we saw no evidence for formation of the analogous H3K9me_2_O N-oxide.

We also investigated whether the JmjC-domain of KDMs, which notably accept H3K9me_2_ can accept H3K_P_4me_2_ as a substrate. Since H3K_P_9me_2_ peptides are difficult to synthesise due to reactivity of the phosphine, we generated H3K_P_9me_2_ in situ from H3K_P_9me_3_ using KDM4E_JmjC_. The results with KDM3A and KDM7B, which naturally catalyse H3K9me_2_ demethylation, provide clear evidence they do not catalyse demethylation of H3K_P_9me_2_, revealing the ability of JmjC KDMs to accept P-analogues is subfamily dependent. As with the results for the readers, the results with JmjC KDMs show very small changes to the substrate, likely due to changes in size or charge, can make large differences in substrate selectivity. We hope that this knowledge will inspire medicinal chemistry efforts to identify JmjC KDM isoform specific inhibitors.

Phosphorous is essential for all life forms where it is principally found in its oxidised phosphate form, in nucleic acids, small molecules (*e.g*., ATP, NADPH), proteins and lipids, amongst other molecules. Alkylated phosphine compounds have, to our knowledge, not been identified in biology. In part this may be due to their tendency to be oxidized, as evident in our work where evidence for KDM4E-catalysed oxidation at H3K_P_9me_2_ to give H3K_P_9me_2_O, rather than H3K9me_1_ was accrued. However, phosphine (PH_3_) is present in the Earth’s atmosphere where it is proposed to be part of the phosphorus cycle^51^ and, may be present in the atmosphere of Venus^52^. Our results show that at least several related enzymes can act on reduced phosphine derivatives, highlighting the possibility that reduced phosphine derivatives might, at least in some specialised contexts, have a biological role, and/or that they may have played a role in the evolution of biology.

## Methods

### Protein production

The following purified reader domains: TAF3_PHD_ (R857-K924), KDM5A_PHD3_ (JARID1A, M1489-V1641), BPTF_PHD_ (L2583-N2751), KDM4A_TTD_ (JMJD2A, Q897-P1011) and SGF29_TTD_ (R115-K293) were prepared as reported^10,22–26^. The histone lysine demethylase were produced and purified to high purity *via* reported procedures KDM3A (JMJD1A, T515-S1317)^41^, KDM4A_JmjC_^39^ (JMJD2A, M1-L359), KDM4E_JmjC_ (JMJD2E, M1-Q337)^34^ and KDM7B^40^ (PHF8, M37-N483 [NP_001171]). KDM5D_JmjC_ was produced using an N-terminal hexa-His tagged KDM4D (KDM4D_M1-L358_) DNA construct, transformed into BL21(DE3) competent cells for recombinant protein production. Colonies were used to inoculate of LB media (50 mL) containing kanamycin (50 μg·mL^−1^) and chloramphenicol (34 μg·mL^−1^), which was placed in a 37 °C shaker overnight. The starter culture (10 mL) was used to inoculate TB media (6 × 1 L) containing kanamycin (50 μg·mL^−1^) in 2 L baffled shaker flasks. After reaching an of OD_600_ of ~0.8, the temperature was reduced to 18 °C; at OD_600_ ~0.9 the cells were induced by the IPTG (0.5 mM) addition. After shaking overnight, the culture was centrifuged (5000 rpm, 10 mins), the media was decanted, and the cell pellet was suspended in Lysis Buffer [HEPES (50 mM), NaCl (500 mM), imidazole (20 mM), glycerol (5%) and TCEP (0.5 mM) in water (pH 7.4)]. The suspension was lysed by passaging through a high-pressure cell breaker (Avestin – EmulsiFlex-C5) for three rounds. The lysate was cleared by centrifugation (60 minutes, 36,000 × *g*, 4°C), then loaded onto a Ni NTA gravity column. After extensive rinsing of the Ni-NTA gravity column with lysis buffer, the His-tagged protein was eluted in lysis buffer containing 300 mM imidazole. The eluted protein was concentrated and subjected to gel filtration chromatography using an AKTA Xpress system, an S200 16/600 gel filtration column, and GF buffer [HEPES (50 mM), NaCl (150 mM), glycerol (5%) and TCEP (0.5 mM) in water (pH 7.4)]. The protein identity was verified by LC-MS (ESI-TOF) observing a mass of 43759.7 Da, in accord with the predicted mass of 43751.6 Da.

### Peptide synthesis

Histone H3 mimic-peptides were prepared (as C-terminal amides) using standard SPPS methodology with *N*^α^-Fmoc protection. Reaction of the C-terminal amino acid with the Wang resin was done by suspending the Wang resin (1.0 g, 1.0 mmolηg^− 1^, 1.0 equivalent) in CH_2_Cl_2_/DMF (9:1, 10 mL), followed by addition of diisopropylcarbodiimide (DIC, 252 mg, 2.0 mmol, 2 equivalents), HOBt (270 mg, 2.0 mmol, 2.0 equivalents), DMAP (12.0 mg, 0.10 mmol, 0.1 equivalents), and the Fmoc-Aa-OH (2.0 mmol, 2.0 equivalents). The solution was stirred slowly for (20 h, rt). Ac_2_O (200 μL, 2.10 mmol, 2.1 equivalents) and pyridine (200 μL, 2.40 mmol, 2.4 equivalents) were then added, and the suspension was stirred for 30 min at rt. The suspension was filtered and the resin washed with DMF, CH_2_Cl_2_ (40 mL), and MeOH (40 mL) (×3), and dried before using in coupling steps.

#### Manual approach

Each coupling reaction was performed in DMF with the appropriate Fmoc-protected amino acid (3.0 equivalents), diisopropylcarbodiimide (DIC, 3.3 equivalents) and hydroxybenzotriazole (HOBt, 3.6 equivalents). Note, coupling of Fmoc-K_P_me_3_-OH was done for an extended period (at least 16 hours). Subsequently, free *N*-terminal amines were capped (Ac_2_O, 2.0 equivalents, pyridine, 2.4 equivalents) before treatment with piperidine. Completion each coupling reactions was determined by the Kaiser test, followed by removal of the Fmoc group by treatment with piperidine (20% v/v in DMF) for 30 min, with completion being determined by the Kaiser test. Washing in between steps was done by treatment of the resin with DMF (3×). Before acidic deprotection and cleavage, the resin was treated with DMF (3×) and Et_2_O (3×), then dried under reduced pressure.

#### Peptide synthesizer approach

Peptides were synthesized using a Liberty Blue microwave assisted solid phase peptide synthesizer (CEM corporation). The coupling steps were carried out using DIC and Oxyma in DMF in a microwave vessel at 90 °C. Each coupling step was performed in DMF with an excess of Fmoc-protected amino acids (5.0 equivalents). Note that the coupling step of Fmoc-K_P_me_3_-OH (2.0 equivalents) was performed manually, using HATU (2.5 equivalents) in DMF for 16 h at rt. Subsequently, any free N-terminal amine was capped using Ac_2_O (2.0 equivalents), and pyridine (2.4 equivalents) before treatment with piperidine.

Cleavage of the peptides was achieved using a mixture of TFA 92.5%, H_2_O 2.5%, triisopropylsilane 2.5%, and ethane-1,2-dithiol 2.5%; the product was precipitated from Et_2_O after 3–4 h. The crude product was suspended in Et_2_O, then centrifuged (3500 rpm, 4 minutes); the supernatant was then decanted (3 times). Purification of the peptides was performed by preparative HPLC. Analysis of the peptides was done by LC-MS and analytical HPLC. Conditions for a typical HPLC purification run were starting conditions: MeCN (3%) in H_2_O (both supplemented with 0.1% (v/v) TFA), a gradient to 100% MeCN over 30 minutes. Sample fractions were pooled based on the results of LC-MS analysis, then lyophilised to yield the desired product as a fluffy powder.

### Isothermal titration calorimetry

ITC studies followed a reported procedure^26^. The buffer used corresponded to that used in the final protein purification step. Briefly, TAF3_PHD_ and KDM4A_TTD_: [Tris (50 mM) in water (pH 7.5)]; KDM5A_PHD3_ and BTPF_PHD_: [Tris (50 mM), NaCl (20 mM) in water (pH 7.5)]; [Tris (25 mM), NaCl (50mM), 1,4-dithiothreitol (1.0 mM) in water (pH 7.5)]. Experiments were conducted using ITC200 automated (GE Healthcare Life Sciences, USA) instrument at 25 °C. Histone peptide titrations were performed with the same reader batches. Solutions of the reader in buffer (25–40 μM) and of the histone H3 peptide (350–600 μM) in buffer were prepared. The prepared solutions were plated into a 96-well plate and inserted into the instrument for analysis. Experiments were performed according to manufacturer’s default settings: Plate pre-rinse syringe clean. A total of 19 injections were performed; each experiment was repeated 3–5 times. Heats of dilution for histone peptides determined in control experiments were subtracted from the titration binding data. Data were analysed with Origin 6.0 (Microcal Inc., Northampton, Massachusetts, USA) and curve fitting with one-site binding mode was applied.

### MALDI-TOF demethylation experiments

A Bruker Daltonics MALDI-TOF/TOF AutoflexSpeed machine and Bruker MTP 384 target plates (polished steel BC, Part: 8280781) were used. The machine was controlled using Flex control (v. 3.4 build 135.10) and Compass for flex series (v. 1.4) software. Measurements were in the positive ion mode with the reflectron mode enabled. Incubations employed ProxiPlate-384™ (Perkin Elmer) plates into which was pipetted a solution of [H3_1-1.5_K9me_3_ (10.0 μM), (+)-sodium L-ascorbate (1.0 mM), (NH4)_2_Fe(II)(SO4)_2_ (100 μM), di-sodium 2-oxoglutarate (200 μM) in buffer (HEPES (50 mM)) in MilliQ (pH 7.5)] (5.0 μL) using ClipTip™ (Thermo Scientific™) pipette tips and E1-ClipTip™ (Thermo Scientific™). The enzyme solution [KDM4E_JmjC_ (1.0 μM) in HEPES (50 mM)] (5.0 μL) was added to initiate reaction at 37 °C. Reactions were quenched with formic acid in water (2%, 5.0 μL). Samples were then spotted onto a MALDI-TOF target plate (1.0 μL), MALDI matrix [sat. sol. α-cyano-4-hydroxycinnamic acid (10 mg·mL^–1^) in {trifluoro acetic acid, acetonitrile and MilliQ (0.1:50:50)}] (1.0 μL) was added, mixed, and dried in air. Samples were then analysed by MALDI-TOF. Specific experiments were supplemented with catalase from bovine liver (C3155-50MG, Merck) (5.0 μM), bovine albumin serum (BSA, Perkin Elmer, CR84-100, DTPA purified 7.5%) (5.0 μM), or Tris(carboxyethyl) phosphine hydrochloride salt (TCEP, M02624, Fluorochem) (500 μM).

### Demethylation experiments using LC-MS

KDM4E_JmjC_ demethylation studies of H3_1–15_K9me_3_ or H3_1–15_K_P_9me_3_ incubations using LC-MS were conducted as reported^31^, using Agilent RapidFire 365 and Agilent QTOF 6530 machines. In brief, samples were aspirated under vacuum (~50 μL), passed through a loop (10 μL, 400 ms) and wash on a SPE cartridge A (C_4_) using solvent A (1.5 mLηmin^–1^, 4500 ms). Peptides were eluted using solvent B (1.25 mLηmin^–1^, 4500 ms) and directed to the MS for measurements. The cartridge was equilibrated for the next sample (1.25 mLηmin^–1^, 500 ms) and needle was cleaned with an organic wash solution. Between each sample, an alternating inorganic, organic and inorganic washes were performed to avoid any potential carry over on the SPE cartridge from previous sample. Solvent A: formic acid (0.1%) in water; Solvent B: formic acid (0.1%), acetonitrile (85%) in water; Inorganic wash: water; Organic wash: acetonitrile. Demethylation reactions were conducted in a temperature-controlled room (22 °C) and the MS machine Real-time monitoring mode. A solution [Peptide (6.0 μM), sodium L-ascorbate (600 μM), (NH_4_)_2_Fe(II)(SO_4_)_2_ (60 μM), disodium 2OG salt (120 μM) in buffer] (550 μL) was prepared. The first time point (~50 μL) was aspirated and acquired in the absence of KDM4E_JmjC_ (t = 0 min). Subsequently, the enzyme solution [KDM4E_JmjC_ (3.0 μM) in buffer] (100 μL) was added and samples from the solution were taken every 2–2.5 min and measured. Note that the time was recorded between the addition and the first aspiration of the enzymatic reaction mixture [Peptide and Enzyme solution mixture]. Each measurement with the corresponding mass profile was time-stamped and the data was processed using Agilent Masshunter (B.06.00), MicroSoft Excel™, GraphPad Prism© (v. 5.0) and Adobe illustrator (15.0.0) software.

### Demethylation experiments studies using NMR

Incubations of H3_1–15_K_P_9me_3_ with KDM4E_JmjC_ were performed in Eppendorf tubes (1.5 mL). Conditions used for ^1^H and ^31^P NMR time-courses: H3_1–15_K_P_9me_3_ (250 μM) was incubated with KDM4E_JmjC_ (50 μM), sodium ascorbate (1.00 mM), 2OG (500 μM), and Fe(NH_4_)_2_(SO_4_)_2_ (100 μM) in HEPES-d_18_ buffer (50 mM, pH 7.5) in D_2_O (> 95% ^2^H). Reactions (160 μL total volume) were quenched by addition of HCl (1 M, 10 equivalents) after the indicated time. The samples were centrifuged (1 min, 14,500 rpm) and the supernatant transferred to an NMR tube (3 mm, Norell). For characterisation of the products from the incubation of H3_1–15_K_P_9me_3_ with KDM4E_JmjC_ the following conditions were used: Peptide H3_1–15_K_P_9me_3_ (500 μM) was incubated with sodium ascorbate (1.00 mM) 2-oxoglutarate (1.00 mM), Fe(NH_4_)_2_(SO_4_)_2_ (100 μM), and KDM4E_JmjC_ (50.0 μM), in HEPES-d_18_ buffer (50 mM, pH 7.5) in D_2_O (>95% ^2^H) for 1 hour. Reactions were quenched by addition of HCl (1 M, 10 equivalents), or by heating (95 °C, 10 min). Precipitated proteins were removed by centrifugation (1 min, 14,500 rpm) and the supernatant transferred to an NMR tube (3 mm, Norell). Spectra were measured using a Bruker 600 MHz machine and analysed using MestReNova 14.1 (MestReLabs, Spain; www.mestrelab.com) and Topspin 3.6.1 (Bruker, Germany; www.bruker.com).

### Quantum chemical analysis

Quantum chemical calculations were performed with the Amsterdam Density Functional software (ADF)^53^ using dispersion-corrected density functional theory at the BLYP-D3BJ/TZ2P level of theory^54^. Our BLYP-D3BJ/TZ2P approach provided results that are in excellent agreement with those of a recent high-level CCSD(T) benchmark study by Varma and coworkers (Supplementary Table 6)^55^. Solvation in water was simulated by means of the conductor like screening model (COSMO) of solvation implemented in ADF^56–59^. The cation-π interactions in TRP2–H3K4me_3_ and TRP2–H3K_P_4me_3_ complexes were analysed through quantitative Kohn–Sham molecular orbital theory combined with energy decomposition analysis (EDA)^60,61^. In this method the bond energy in water Δ*E*(aq) is a combination of the strain energy (Δ*E*_strain_(aq)) associated with deforming the cation and the reader from their equilibrium structures to the geometry they adopt in the complex, combined with the interaction energy (Δ*E*_int_(aq)) between these deformed fragments in the complex:

(1) $$\text{Δ}E(\text{aq})=\text{Δ}E_{\text{strain }}(\text{aq})+\text{Δ}E_{\text{int }}(\text{aq})$$

The role of desolvation in the complexation process can be analysed by splitting the solute–solute interaction (Δ*E*_int_(aq)) into the effect caused by the change in solvation (Δ*E*_int_(desolv)) and the remaining intrinsic interaction (Δ*E*_int_) between the unsolvated fragments in vacuum:

(2) $$\text{Δ}E_{\text{int }}(\text{aq})=\text{Δ}E_{\text{int }}(\text{ desolv })+\text{Δ}E_{\text{int }}$$

The interaction energy Δ*E*_int_ can be further decomposed by:

(3) $$\text{Δ}E_{\text{int}}=\text{Δ}V_{\text{elstat}}+\text{Δ}E_{\text{Pauli }}+\text{Δ}E_{\text{oi}}+\text{Δ}E_{\text{disp}}$$

where, Δ*V*_elstat_ corresponds to the classical electrostatic interaction between the unperturbed charge distributions of the deformed fragments, which is usually attractive. The Pauli repulsion (Δ*E*_Pauli_) term comprises the destabilizing interactions between occupied orbitals and is responsible for steric repulsions. The orbital interaction (Δ*E*_oi_) accounts for charge transfer (donor–acceptor interactions between occupied orbitals on one moiety with unoccupied orbitals of the other, including the HOMO–LUMO interactions) and polarization (empty/occupied orbital mixing on one fragment due to the presence of another fragment). Finally, the Δ*E*_disp_ term accounts for the dispersion interactions based on Grimme’s DFT-D3BJ correction. The charge distribution was analysed using the Voronoi deformation density (VDD) method^62^.

### Molecular dynamics simulations

MD simulations were carried out for 10 ns. Crystal structures for the models representing TAF3_PHD_ (PDB: 2K17), KDM4ATTD (PDB: 2GFA), KDM5A_PHD3_ (PDB: 2KGI), BPTF_PHD_ (PDB: 2F6J), and SGF29_TTD_ (PDB: 3ME9) readers were used as starting structures for the protein-ligand modelling. Starting structures were built by manually replacing the Kme_3_ residue of H3K4me_3_ with K_P_9me_3_ residue in the reader protein crystal structures complexes. AMBER12^30^ was used with the Amberff12SB force field to define protein partial charges. Hydrogen atom addition was performed with LEaP. Systems were solvated in a 10 Å truncated octahedral box of TIP3P^29^ water and neutralised explicitly with either sodium or chloride counterions. Non-bonding parameters of Zn(II), previously established from studies of KDM4A^63^, were employed. Atomic partial charges for H3K_p_9me_3_ correspond to the Restrained Electrostatic Potential (RESP)^64^ charges, as shown in Supplementary Table 2. Parameters for Kme_3_ were taken from previous work^31^. The final systems were minimised for 1,000 cycles of steepest-descent minimization followed by 1,000 cycles of conjugate-gradient minimization to remove close van der Waals contacts using the sander program in AMBER12. Equilibration was achieved using PMEMD to heat the systems to 310 K followed by independent MD simulations performed with a periodic boundary condition at a constant pressure of 1 atm with isotropic molecule-based scaling at a time step of 2.0 fs. All simulations used a dielectric constant of 1.0, Particle Mesh Ewald summation^65^ to calculate long-range electrostatic interactions and bond-length constraints applied to all bonds to H atoms. Trajectories were saved at 20 ps intervals and visualised using VMD^66^.

### Reporting summary

Further information on research design is available in the Nature Research Reporting Summary linked to this article.

## Supplementary Material

**Supplementary information** The online version contains supplementary material available at https://doi.org/10.1038/s42004-022-00640-4.

Reporting Summary

Supplementary Information

## Figures and Tables

**Fig. 1:**
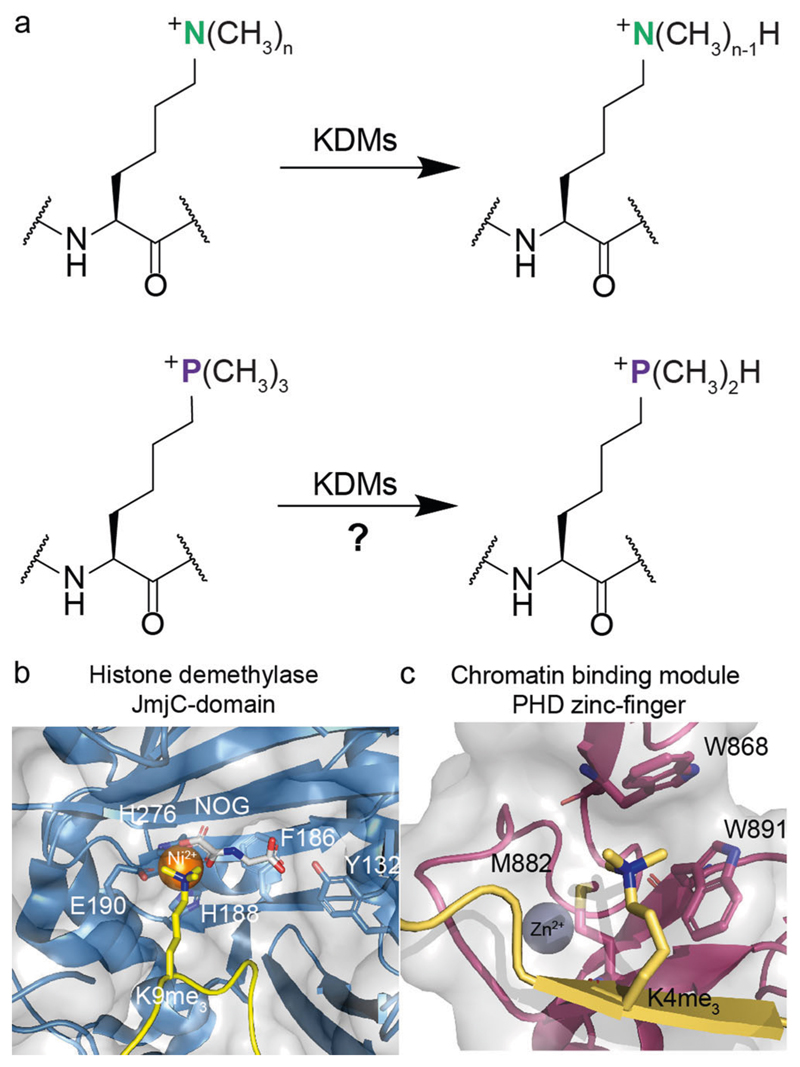
Demethylation and recognition of *N*^ε^-methylated lysines by erasing enzymes and reader proteins. **a** JmjC KDMs catalyse demethylation of *N^ε^-*trimethyllysine residues. Our work explored recognition of the simplest positively charged *N*^ε^-trimethyllysine analogue, i.e., the trimethylphosphonium derivative, by *N*^ε^-methyllysine binding proteins and *N*^ε^-methyllysine demethylases. n: number of methyl groups (3-1). **b** View from a structure of a JmjC KDM (KDM4A_Jm_j_C_, light blue) complexed with H3K9me_3_ (yellow) and NOG (N-oxalylglycine, a 2OG analogue, white) (PDB: 2OQ6). **c** View from a structure of a reader (TAF3_PHD_, purple) complexed with H3K4me_3_ (yellow) (PDB: 2K17). Nitrogen: dark blue; oxygen: red; sulphur: yellow; zinc: grey; nickel: orange.

**Table 1:** Thermodynamic parameters for association of H3K4me_3_ and H3K_P_4me_3_ peptides (ART(Kme_3_/K_P_me_3_)QTARKS) with five human reader domains^a^.

	H3K4me_3_				H3K_P_4me_3_			
	*K*_d_ (μM)	Δ*G*° (kcal mol^−1^)	Δ*H*° (kcal mol^−1^)	-TΔS° (kcal mol^−1^)	*K*_d_ (μM)	Δ*G*° (kcal mol^−1^)	Δ*H*° (kcal mol^−1^)	–TΔS° (kcal mol^−1^)
KDM5A_PHD3_	0.52	−8.6 ± 0.1	−10.4 ± 0.1	1.8 ± 0.1	0.20	−9.1 ± 0.1	−11.0 ± 0.1	1.9 ± 0.1
^TAF3^PHD	0.11	−9.5 ± 0.1	−10.7 ± 0.1	1.2 ± 0.1	0.048	−10.0 ± 0.1	−11.1 ± 0.1	1.1 ± 0.1
BPTF_PHD_	1.4	− 8.0 ± 0.1	−12.5 ± 0.1	4.5 ± 0.1	0.20	−9.1 ± 0.1	−13.8 ± 0.1	4.7 ± 0.1
SGF29_TTD_	3.2	−7.5 ± 0.1	−8.1 ± 0.1	0.6 ± 0.1	2.8	−7.6 ± 0.1	−8.6 ± 0.1	1.0 ± 0.1
KDM4A_TTD_	3.5	−7.4 ± 0.1	−13.0 ± 0.1	5.6 ± 0.1	0.30	−8.9 ± 0.1	−17.7 ± 0.1	8.8 ± 0.1

a Values obtained from 3 to 5 ITC experiments. Errors represent standard deviations.

**Table 2:** Quantum-chemical analyses (calculated energies in kcal mol^–1^, distances in Å) for the TRP2-Kme_3_ and TRP2-K_P_me_3_ complexes in water^a^.

	TRP2-Kme_3_^[b]^	TRP2-K_P_me_3_^[c]^
Δ*E*(aq)	–10.2	–11.4
Δ*E*(aq)_strain_	0.1	0.8
Δ*E*(aq)_int_	–10.3	–12.2
Δ*E*(desolv)_int_	17.3	15.7
Δ*E*_int_	–27.6	–28.0
Δ*E*_int_	20.8	23.5
Δ*V*_elstat_	–15.0	–15.8
Δ*E*_oi_	–13.0	–13.6
Δ*E*_disp_	–20.4	–22.0
d(H_Me_-C_TRP-6MR_)	2.88	2.90
d(H_Me_-C_TRP-5MR_)	2.78	2.68

a Computed using BLYP-D3BJ/TZ2P with COSMO to simulate aqueous solution. Structural rigidity imposed by the protein backbone is simulated through constrained geometry optimizations. See Eqs. (1-3) in Supplementary Information.

b TRP2 fixed, Kme_3_ free.

c TRP2 frozen, α-methyl carbon of K_P_me_3_ fixed to position in TRP2-Kme_3_ optimization.

## Data Availability

The authors declare that the main data supporting the findings of this study are available within the paper and its Supplementary Information file. Other relevant data are available from the corresponding authors upon reasonable request.
