# Supplementary material for: Reading and erasing of the phosphonium analogue of trimethyllysine by epigenetic proteins
Source: Commun Chem. 2022 Mar 7;5:27. doi: 10.1038/s42004-022-00640-4 (PMC7613515; doi:10.1038/s42004-022-00640-4)
Supplement: Supplementary file 1 — Supplementary Information [file 42004_2022_640_MOESM1_ESM.pdf]

# Supplementary Information

# Contents


1 Supplementary figures ........ 3

2 Supplementary Methods ........ 32

2.1 General experimental conditions ........ 32

2.2 Synthesis of Fmoc-Lys(Phosme$_3$)-OH ........ 33

3 References ........ 36

4 Spectral data for synthesised compounds ........ 37

# 1 Supplementary figures

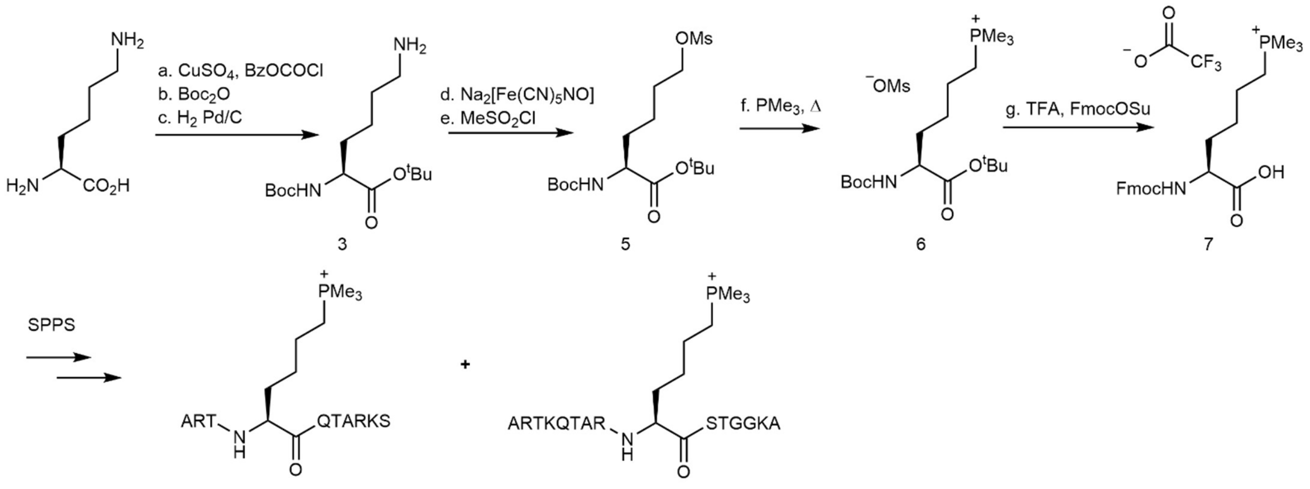


**Supplementary Scheme 1: Route for preparation of phosphonium salt 7 and of peptides using solid phase peptide synthesis (SPPS).** Reagents and conditions: a) 1) $CuSO_4$, $NaHCO_3$, benzyl chloroformate, $H_2O$, rt, 2 h; 2) $Boc_2O$, $NaHCO_3$, $H_2O$ : 1,4-dioxane (1 : 1), rt, 16 h, 47% (over 2 steps); b) $Boc_2O$, DMAP, $^t$BuOH, 30 °C, 20 h, 87%; c) 10% Pd/C, $H_2$, MeOH, rt, 24 h, 88%; d) $Na_2[Fe(CN)_5NO]$, borate buffer pH ~ 9.5, $H_2O$ : 1,4-dioxane (4 : 1), 60 °C, 24 h, 52%; e) $MeSO_2Cl$, $Et_3N$, $CH_2Cl_2$, rt, 3 h, 74%; f) microwave, trimethylphosphine, toluene, 150 °C, 2 h, quantitative; g) 1) TFA, rt, 2 h, 2) Fmoc-OSu, $NaHCO_3$, $H_2O$ : 1,4-dioxane (1 : 1), rt, 16 h, 51%. Fmoc-monomers were incorporated into 10-mer or 15-mer peptides (with C-terminal amides) using solid phase peptide synthesis (SPPS) and purified using HPLC.

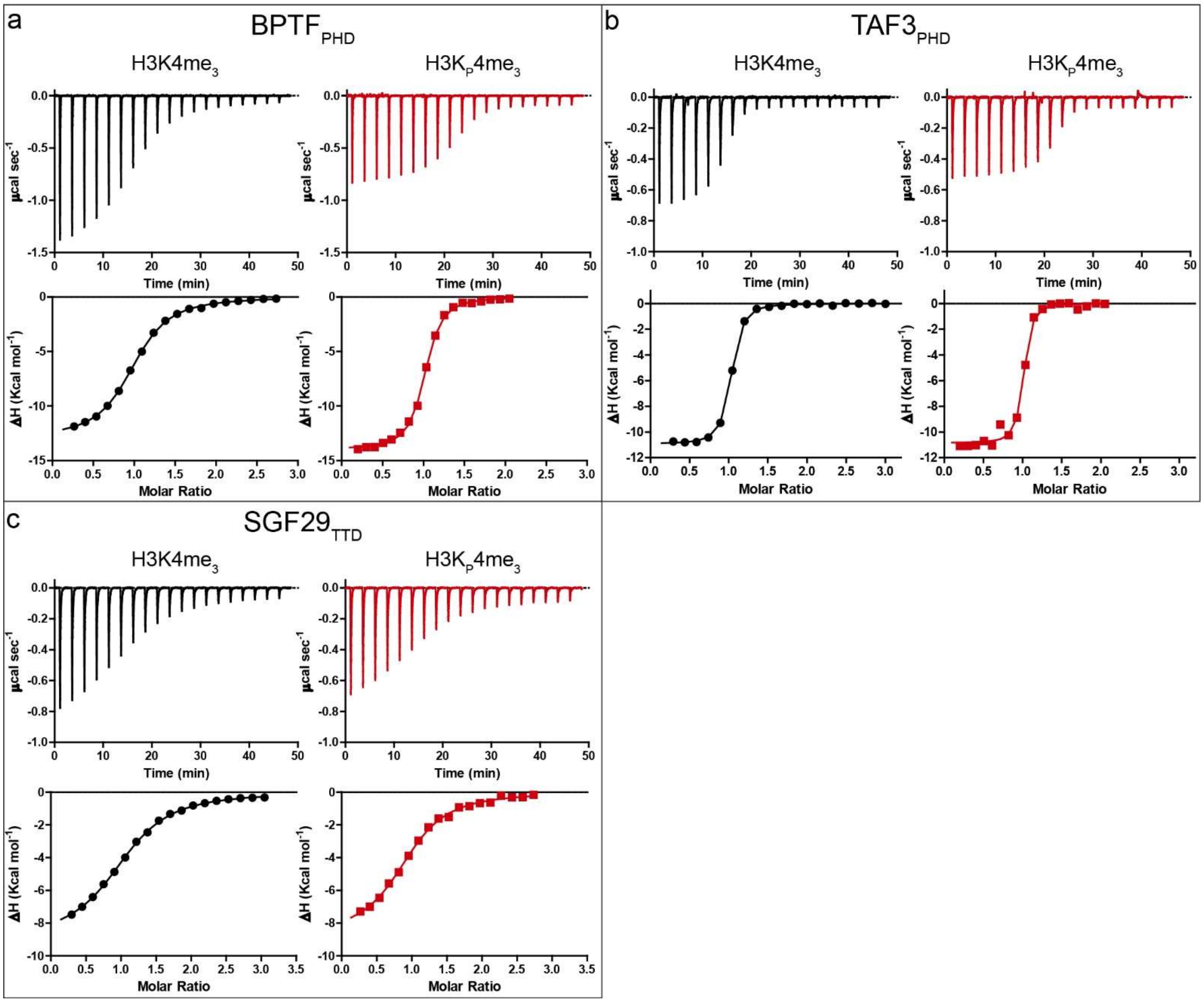


**Supplementary Fig. 1** Representative ITC plots of $H3K4me_3$ and $H3K_{P4}me_3$ titrated with: **a** $BPTF_{PHD}$; **b** $TAF3_{PHD}$ and, **c** $SGF29_{TTD}$. In each case, the top panel shows the raw ITC data; the bottom panel shows the integrated peak and processed data.

**Supplementary Table 1** Calculated average root mean square deviations (RMSDs) and standard deviations of $C^{\alpha}$ atoms of modelled reader proteins in complex with $H3K4me_3$ or $H3K_P4me_3$.

| | RMSD (Å) | | | |
|---|---|---|---|---|
| System | $H3K4me_3$ | | $H3K_P4me_3$ | |
| | Reader | H3 | Reader | H3 |
| $BPTF_{PHD}$ | 5.85 ± 2.49 | 0.47 ± 0.20 | 4.14 ± 1.13 | 0.56 ± 0.20 |
| $KDM4A_{TTD}$ | 2.08 ± 0.64 | 1.17 ± 0.27 | 2.73 ± 0.59 | 1.00 ± 0.31 |
| $KDM5A_{PHD3}$ | 2.45 ± 0.58 | 1.16 ± 0.34 | 3.85 ± 1.01 | 1.07 ± 0.49 |
| $SGF29_{TTD}$ | 1.28 ± 0.20 | 1.03 ± 0.45 | 1.15 ± 0.20 | 0.81 ± 0.29 |
| $TAF3_{PHD}$ | 3.25 ± 0.82 | 3.24 ± 1.20 | 3.62 ± 1.02 | 2.98 ± 0.72 |

**Supplementary Table 2** Cartesian coordinates and charges calculated using the Restrained Electrostatic Potential (RESP) method HF/6-31G* for the H3K$_P$4me$_3$ peptide.

| H3K$_P$4me$_3$ | | | | RESP |
|---|---|---|---|---|
| Atom | X | Y | Z | Charge |
| N | -3.666 | 1.475 | 0.238 | -1.0005 |
| C | -3.561 | 0.213 | -0.462 | 0.4357 |
| C | -2.237 | -0.488 | -0.112 | -0.3177 |
| C | -1.018 | 0.324 | -0.550 | 0.1445 |
| C | 0.299 | -0.288 | -0.063 | 0.0854 |
| C | 1.499 | 0.569 | -0.505 | -0.3835 |
| C | -4.710 | -0.741 | -0.173 | 0.3709 |
| O | -5.513 | -0.552 | 0.680 | -0.4659 |
| C | 3.468 | -1.680 | -0.554 | -0.5899 |
| C | 4.400 | 1.116 | -0.572 | -0.5899 |
| C | 3.210 | -0.019 | 1.872 | -0.5899 |
| H | -3.978 | 1.310 | 1.178 | 0.3783 |
| H | -4.368 | 2.052 | -0.184 | 0.3783 |
| H | -3.567 | 0.409 | -1.533 | -0.0041 |
| H | -2.209 | -1.474 | -0.570 | 0.0768 |
| H | -2.208 | -0.641 | 0.965 | 0.0768 |
| H | -1.001 | 0.394 | -1.636 | 0.0033 |
| H | -1.117 | 1.332 | -0.168 | 0.0033 |
| H | 0.402 | -1.298 | -0.451 | 0.0073 |
| H | 0.277 | -0.363 | 1.020 | 0.0073 |
| H | 1.546 | 0.626 | -1.589 | 0.1262 |
| H | 1.388 | 1.587 | -0.144 | 0.1262 |
| H | -4.749 | -1.645 | -0.790 | 0.0113 |
| H | 3.408 | -1.698 | -1.636 | 0.1945 |
| H | 4.464 | -1.988 | -0.256 | 0.1945 |
| H | 2.752 | -2.385 | -0.152 | 0.1945 |
| H | 4.380 | 1.129 | -1.656 | 0.1945 |
| H | 5.382 | 0.795 | -0.246 | 0.1945 |
| H | 4.223 | 2.121 | -0.208 | 0.1945 |
| H | 2.991 | 0.968 | 2.261 | 0.1945 |
| H | 2.496 | -0.723 | 2.281 | 0.1945 |
| H | 4.204 | -0.307 | 2.195 | 0.1945 |
| P | 3.129 | -0.010 | 0.062 | 0.9599 |

**Supplementary Table 3** Molecular orbital overlaps between the HOMO and HOMO-1 of TRP2 and the LUMO and LUMO+1 of H3K4me$_3$ and H3K$_P$4me$_3$.[a]

| TRP2 MOs | Kme$_3$/K$_P$me$_3$ MOs | TRP2-Kme$_3$ | TRP2-K$_P$me$_3$ |
|---|---|---|---|
| HOMO | LUMO | 0.012 | 0.013 |
| HOMO | LUMO+1 | 0.006 | 0.042 |
| HOMO-1 | LUMO | 0.028 | 0.027 |
| HOMO-1 | LUMO+1 | 0.006 | 0.012 |

[a] Computed using BLYP-D3BJ/TZ2P.

**Supplementary Table 4** EDA analysis (calculated energies in kcal·mol$^{-1}$, distances in Å) homolytic formation of C-H bonds in H3K4me$_3$ and H3K$_P$4me$_3$ systems in aqueous solution.[a]

| | K4me$_3$ | K$_P$4me$_3$ |
|---|---|---|
| $\Delta E$(aq) | –104.7 | -99.0 |
| $\Delta E$(aq)$_{strain}$ | 12.8 | 17.0 |
| $\Delta E$(aq)$_{int}$ | –117.5 | -116.0 |
| $\Delta E$(desolv)$_{int}$ | 3.3 | 3.5 |
| $\Delta E_{int}$ | –120.8 | -119.5 |
| $\Delta E_{Pauli}$ | 96.1 | 93.2 |
| $\Delta V_{elstat}$ | –62.7 | -57.3 |
| $\Delta E_{oi}$ | –153.3 | -154.5 |
| $\Delta E_{disp}$ | –0.8 | -0.8 |
| d(H$_{Me}$-C$_{Me}$) | 2.88 | 2.90 |
| d(H$_{Me}$-C$_{XMe3}$) | 2.78 | 2.68 |

[a] Computed using BLYP-D3BJ/TZ2P with COSMO to simulate aqueous solution. See Supplementary Fig. 9 for geometries.

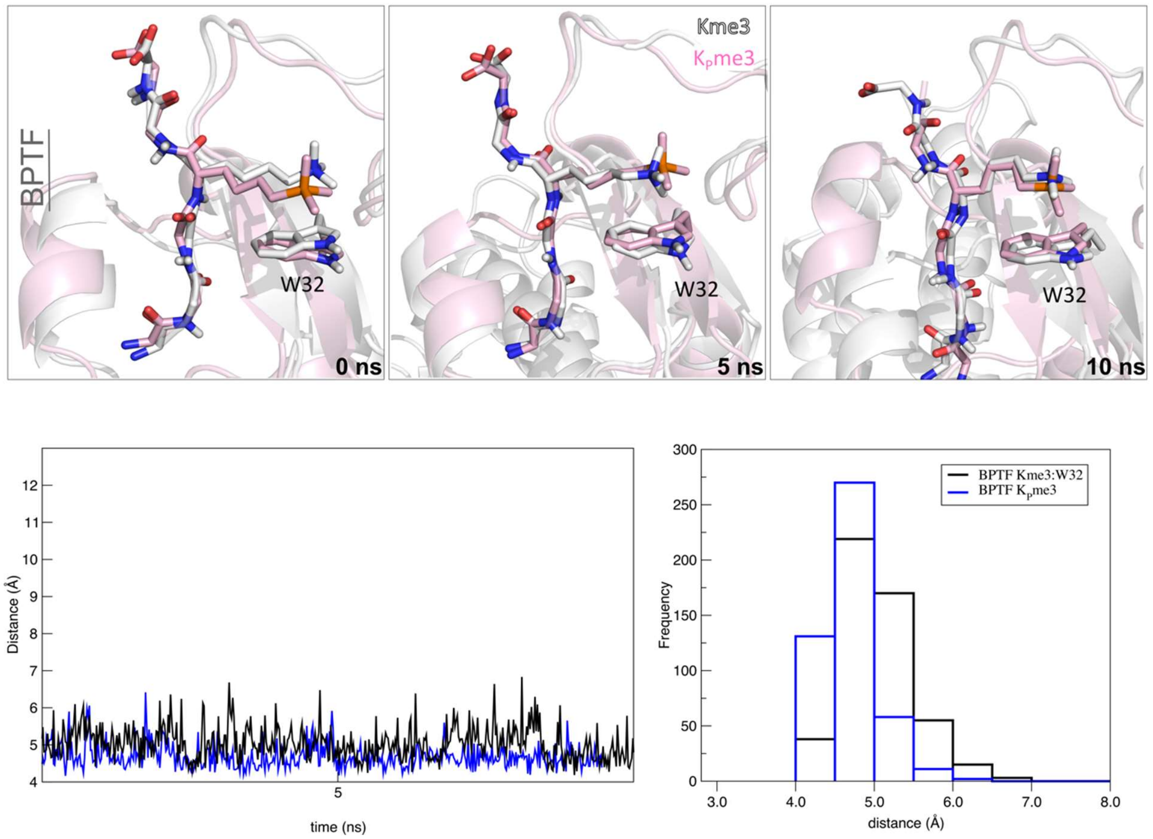


**Supplementary Fig. 2 Molecular Dynamic Simulation of $BPTF_{PHD}$ complexed with $H3K4me_3$ (Kme3) or $H3K_P4me_3$ ($K_Pme3$).** (Top) Snapshots of $BPTF_{PHD}$ complexed with a histone H3 fragment (liquorice) containing $H3K_P4me_3$ (pink) or $H3K4me_3$ (white) at 0 ns, 5 ns, and 10 ns. (Bottom) Distance versus time plots of the $N^+$ /$P^+$ atoms of $H3K4me_3$ or $H3K_P4me_3$, respectively, to the W32 side chain centre of mass over 10 ns.

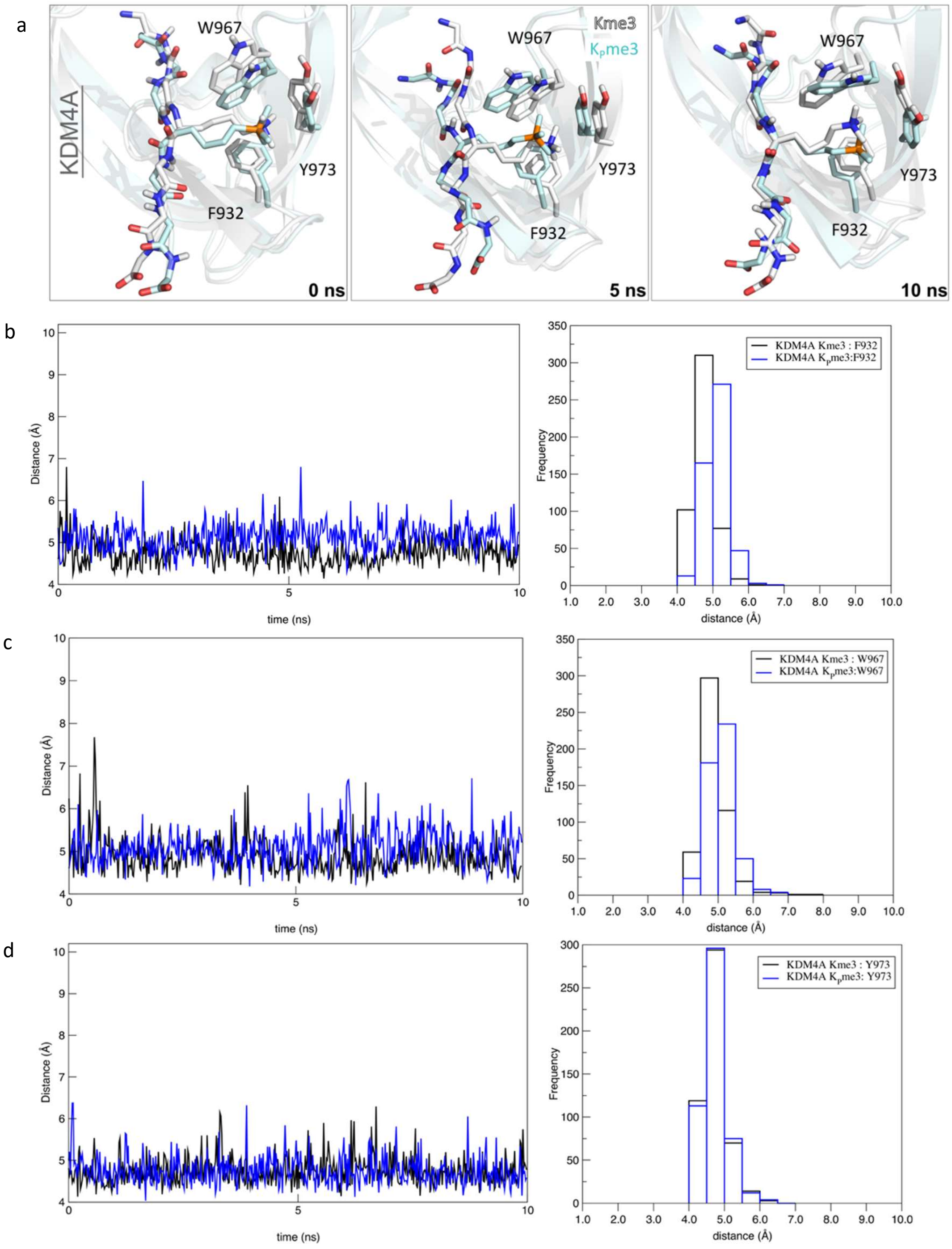


**Supplementary Fig. 3 Molecular dynamic simulation of KDM4A$_{TTD}$ complexed with H3K4me$_3$ (Kme3) or H3K$_P$4me$_3$ (K$_P$me3). a** Snapshots of KDM4A$_{TTD}$ complexed with a histone H3 chain fragment (liquorice) containing H3K$_P$4me$_3$ (cyan) or H3K4me$_3$ (white) at 0 ns, 5 ns, and 10 ns. **b-d** Distance versus time plots of the $N^+$/$P^+$ atoms of H3K4me$_3$ or H3K$_P$4me$_3$, respectively, to the F932, W967, and Y973 side chain centres of mass over 10 ns.

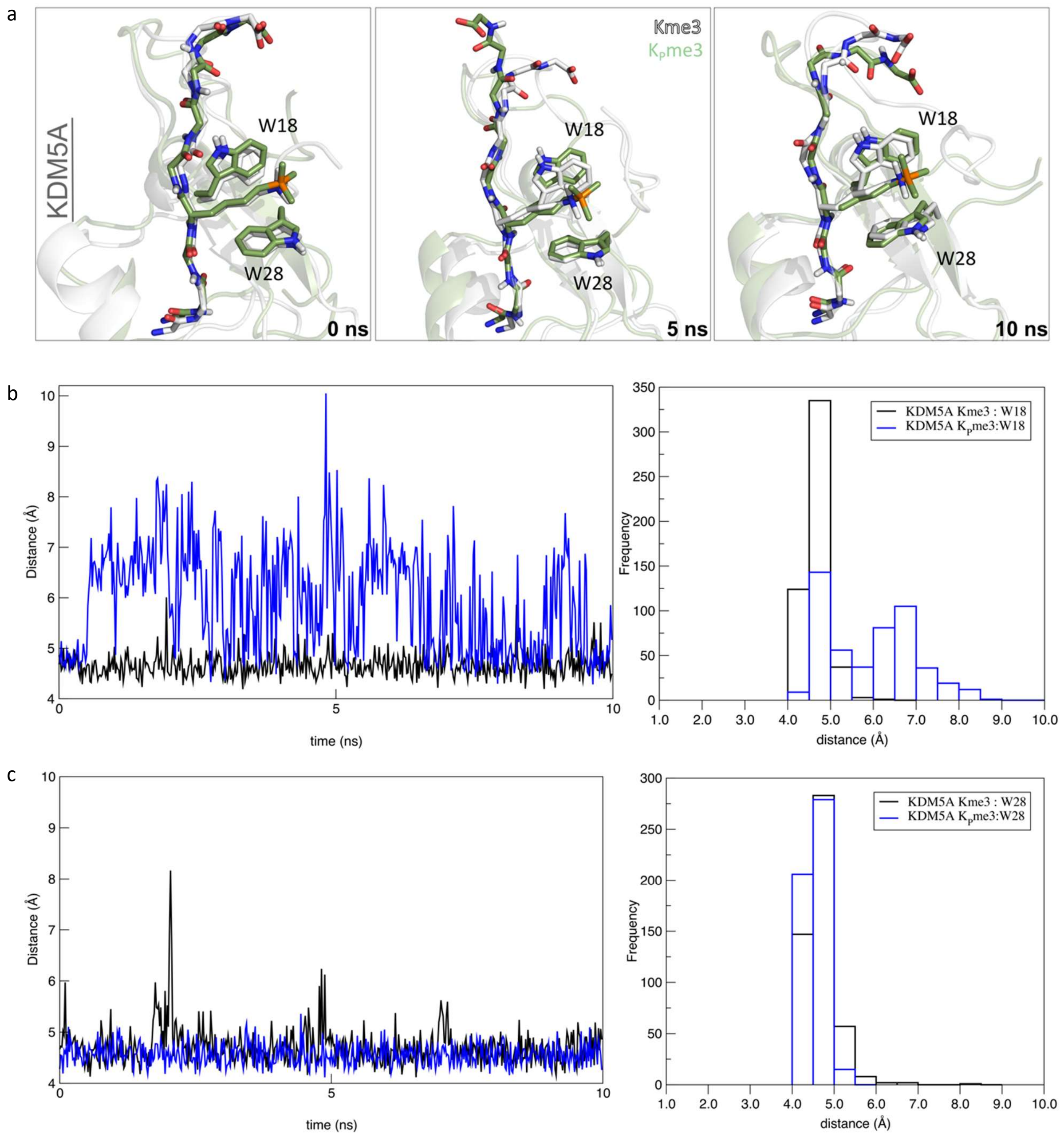


**Supplementary Fig. 4 Molecular dynamic simulation of KDM5A$_{PHD3}$ complexed with H3K4me$_3$ (Kme3) or H3K$_P$4me$_3$ (K$_P$me3). a** Snapshots of KDM5A$_{PHD3}$ complexed with a histone H3 chain fragment (liquorice) containing H3K$_P$4me$_3$ (green) or H3K4me$_3$ (white) at 0 ns, 5 ns, and 10 ns. **b-c** Distance versus time plots of the $N^+$/$P^+$ atoms of H3K4me$_3$ or H3K$_P$4me$_3$, respectively, to W18 and W28 side chain centres of mass over 10 ns.

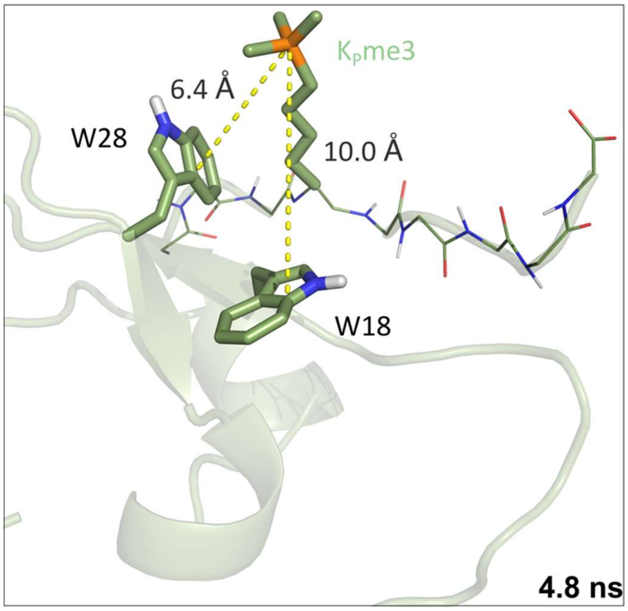


**Supplementary Fig. 5 Molecular dynamic simulation derived view of KDM5A$_{PHD3}$ complexed with H3K$_P$4me$_3$ (K$_P$me$_3$) at time 4.8 ns, when the N$^+$ side chain atom of H3K$_P$4me$_3$ reaches a maximum distance to the W28 side chain.**

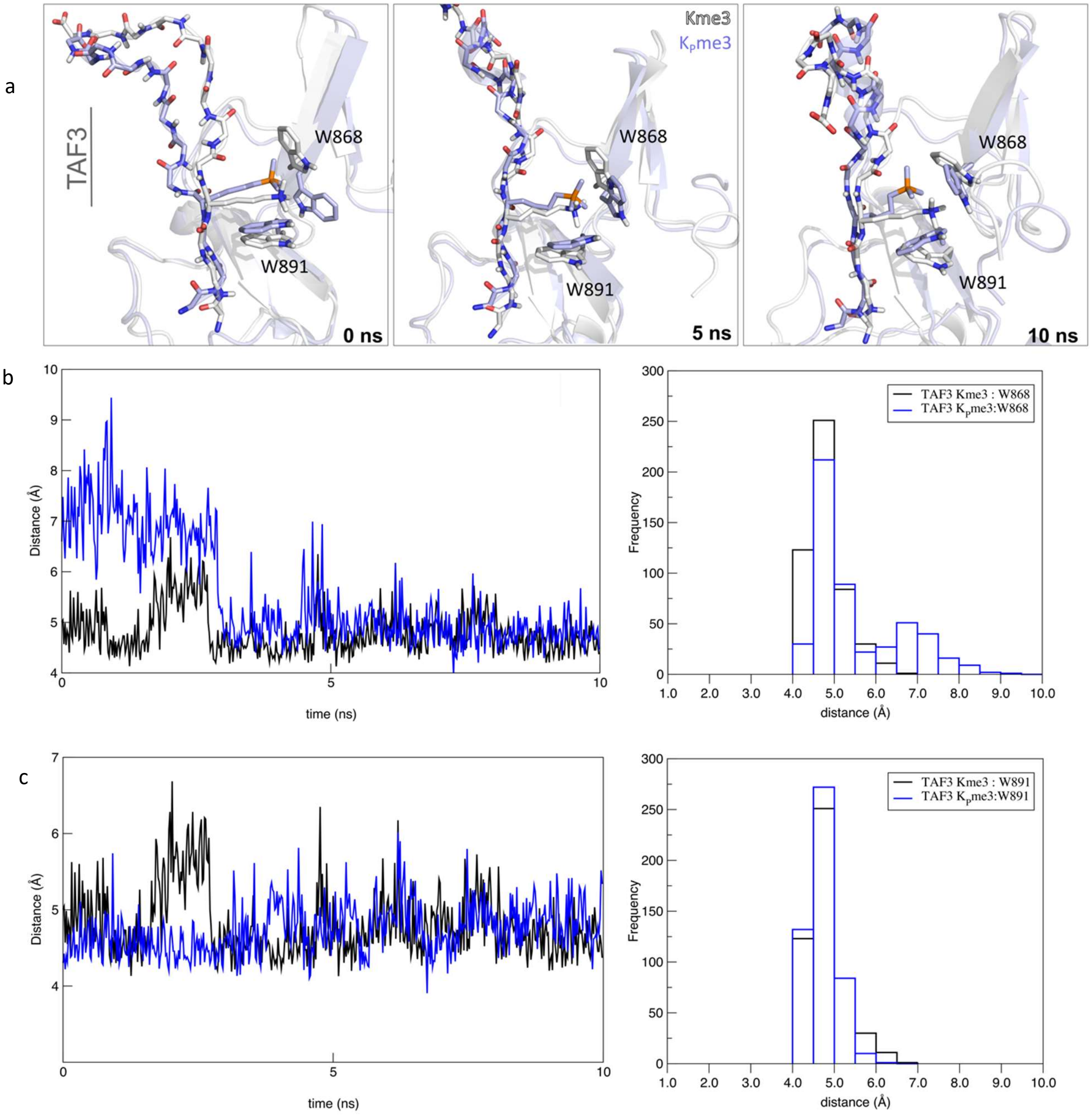


**Supplementary Fig. 6 Molecular dynamic simulation of TAF3$_{PHD}$ complexed with H3K4me$_3$ (Kme3) or H3K$_P$4me$_3$ (K$_P$me3).** **a** Snapshots of TAF3$_{PHD}$ complexed with a histone H3 fragment (liquorice) containing H3K$_P$4me$_3$ (blue) or H3K4me$_3$ (white) at times 0 ns, 5 ns, and 10 ns. **b,c** Distance versus time plots of the $N^+$/$P^+$ atoms of H3K4me$_3$ or H3K$_P$4me$_3$, respectively, to the W868 and W891 side chain centres of mass over 10 ns.

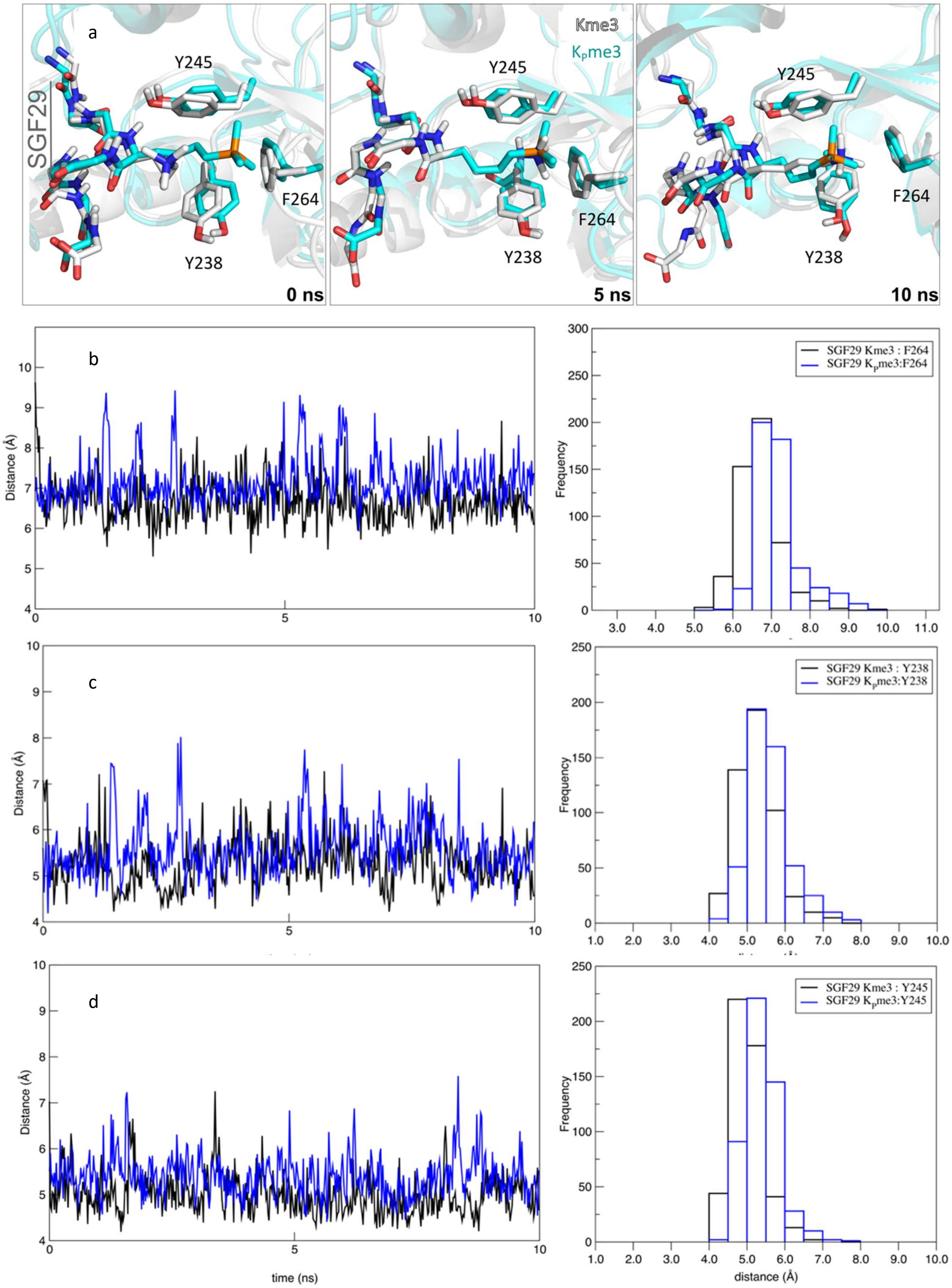


**Supplementary Fig. 7 Molecular Dynamic simulation of $SGF29_{TTD}$ complexed with H3K4me$_3$ (Kme3) or H3K$_P$4me$_3$ (K$_P$me3).** **a** Snapshots of $SGF29_{TTD}$ complexed with a histone H3 chain fragment (liquorice) containing H3K$_P$4me$_3$ (cyan) or H3K4me$_3$ (white) at 0 ns, 5 ns, and 10 ns. **b-d** Distance versus time plots of the $N^+$ /$P^+$ atoms of H3K4me$_3$ or H3K$_P$4me$_3$, respectively, to the F264, Y238, and Y245 side chain centres of mass over 10 ns.

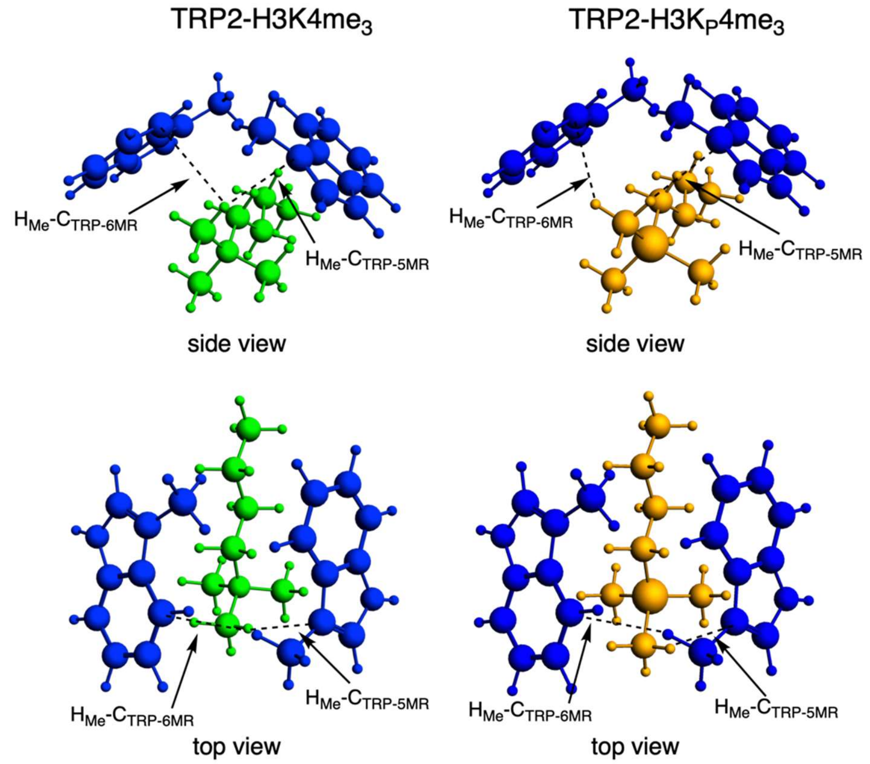


**Supplementary Fig. 8** Side and top geometrical views of TRP2-Kme$_3$ and TRP2-K$_P$me$_3$ model complexes: TRP2 in blue, Kme$_3$ in green, and K$_P$me$_3$ in orange.

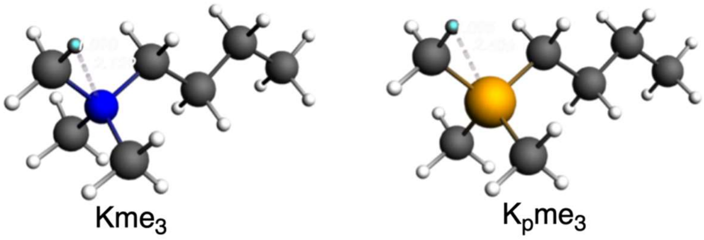


**Supplementary Fig. 9** Geometries of the Kme$_3$ and K$_P$me$_3$ systems with the cleaved proton in blue. Bond lengths (dashed line) enclosed in Supplementary Table 4.

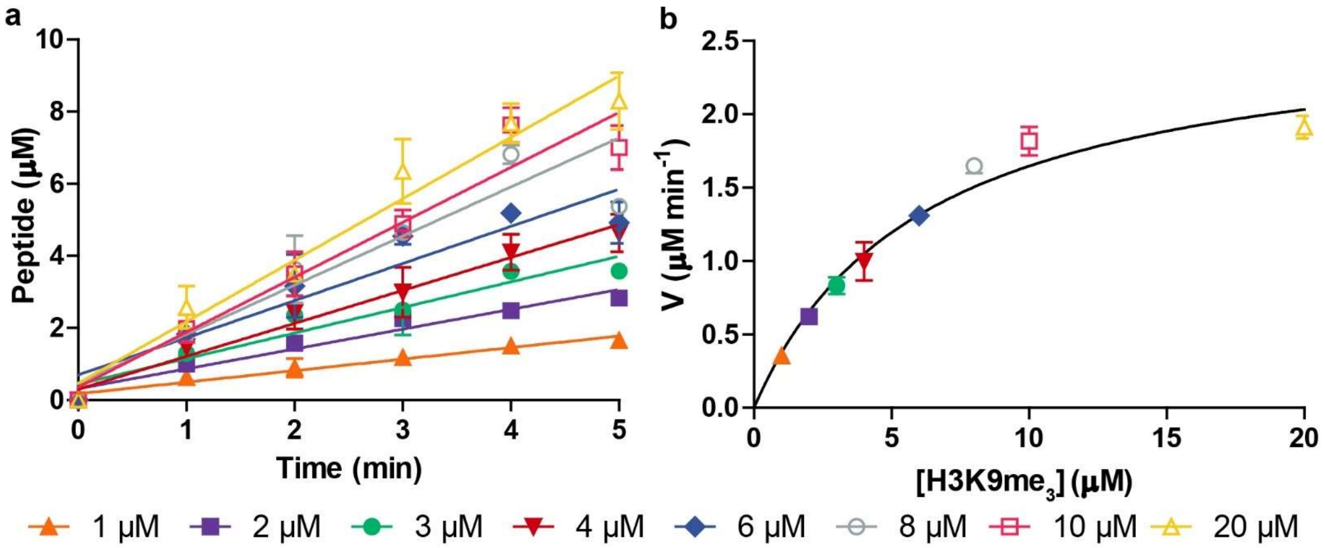


**Supplementary Fig. 10 MALDI-TOF assay time-course and kinetic analysis of KDM4E$_{JmjC}$ (0.5 μM) catalysed demethylation of H3K9me$_3$ (1–20 μM). a** Time-course of KDM4E$_{JmjC}$ against H3K9me$_3$ (1–20 μM). **b** Michaelis-Menten enzyme kinetic analysis. Standard conditions: Asc (500 μM), Fe(II) (50 μM) and 2OG (100 μM). Errors are standard deviations of the mean (n=2).

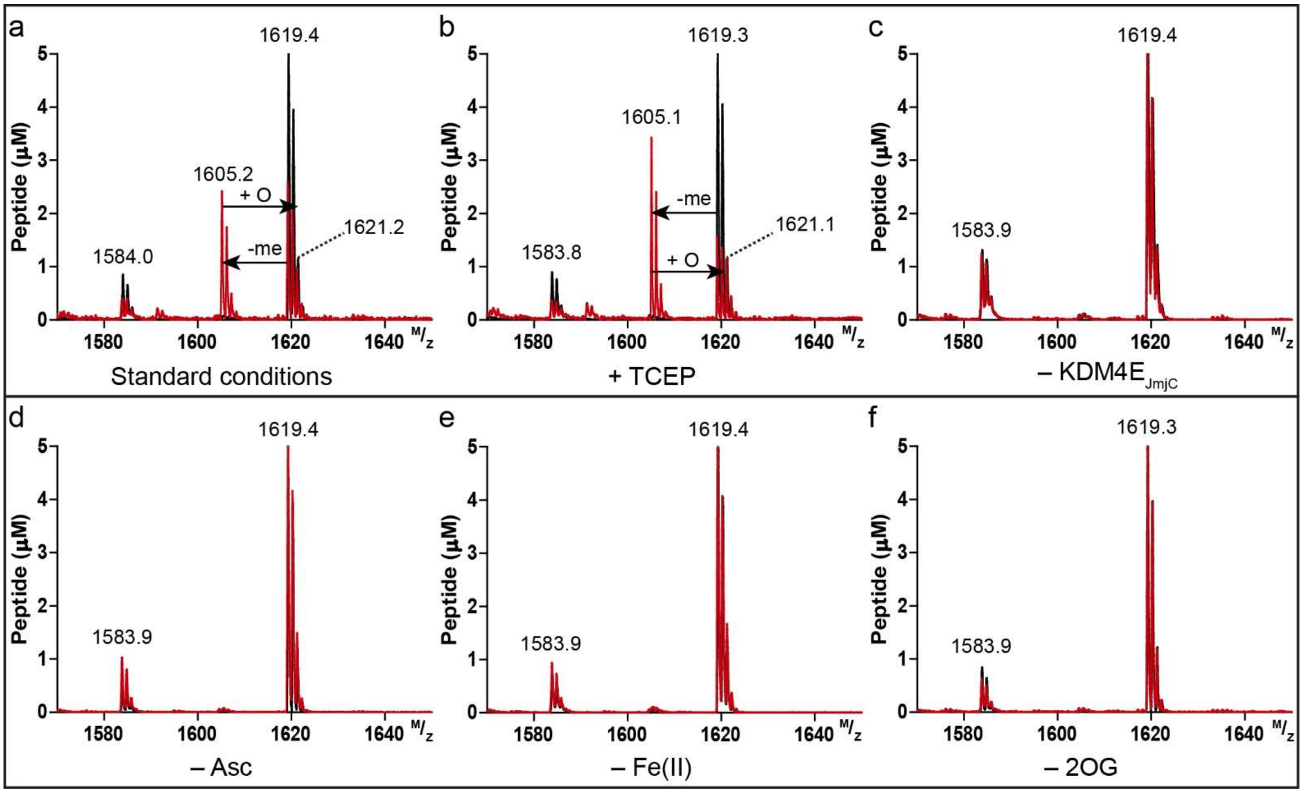


**Supplementary Fig. 11 MALDI-TOF Activity assay of KDM4E$_{JmjC}$ (0.5 µM) catalysed demethylation of H3K$_P$9me$_3$ (5.0 µM) demonstrating that Asc, Fe(II), 2OG and KDM4E$_{JmjC}$ enable or promote demethylation.** **a** Standard conditions, **b** with TCEP, **c** without KDM4E$_{JmjC,}$ **d** without Asc, **e** without Fe(II) and **f** without 2OG. Standard conditions: Asc (500 µM), Fe(II) (50 µM) and 2OG (100 µM), 1 hr. Black t: 0 min, red t: 60 min.

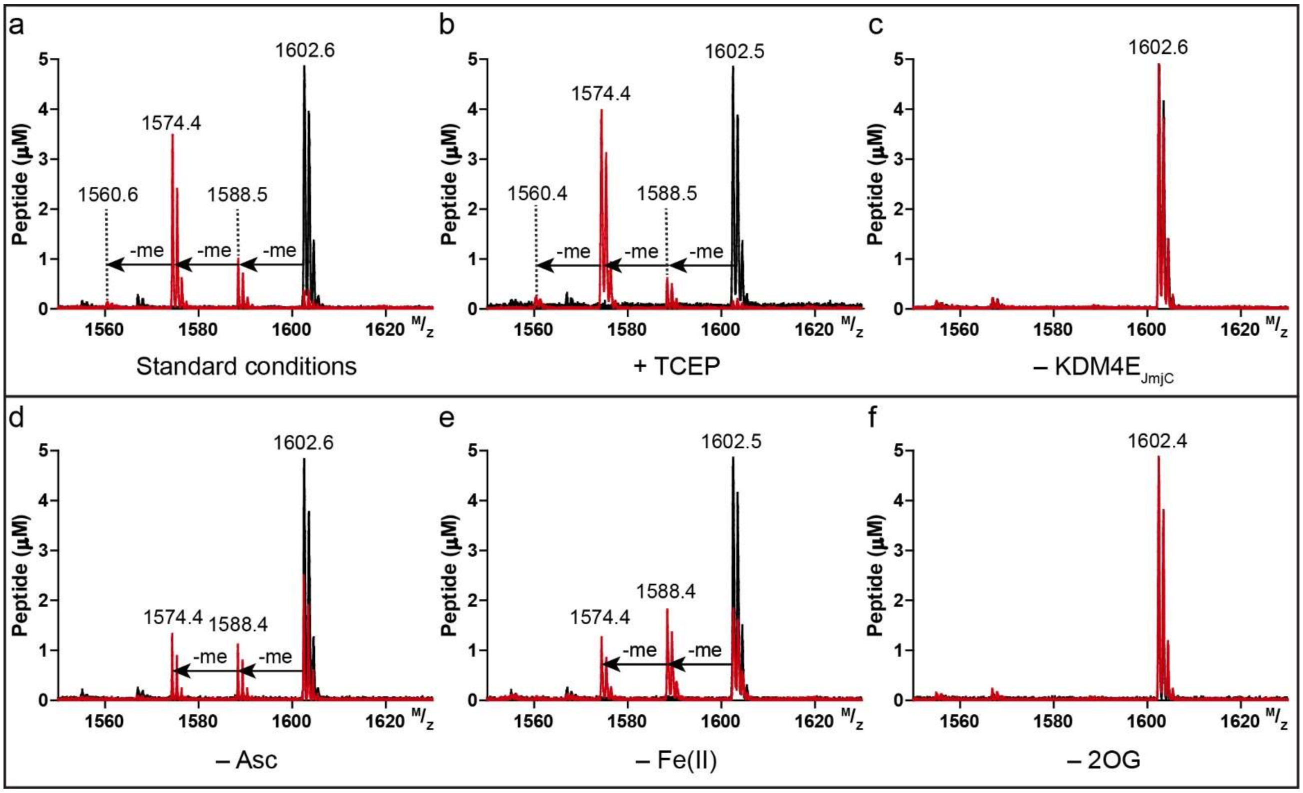


**Supplementary Fig. 12 MALDI-TOF activity assay of KDM4E$_{JmjC}$ (0.5 μM) catalysed demethylation H3K9me$_3$ (5.0 μM) demonstrating that 2OG and KDM4E$_{JmjC}$ are required. The addition of Asc, Fe(II) and TCEP enhances demethylation. a** Standard conditions, **b** with TCEP, **c** without KDM4E$_{JmjC}$, **d** without Asc, **e** without Fe(II) and **f** without 2OG. Standard conditions: Asc (500 μM), Fe(II) (50 μM) and 2OG (100 μM), 1 hr. Black t: 0 min, red t: 60 min.

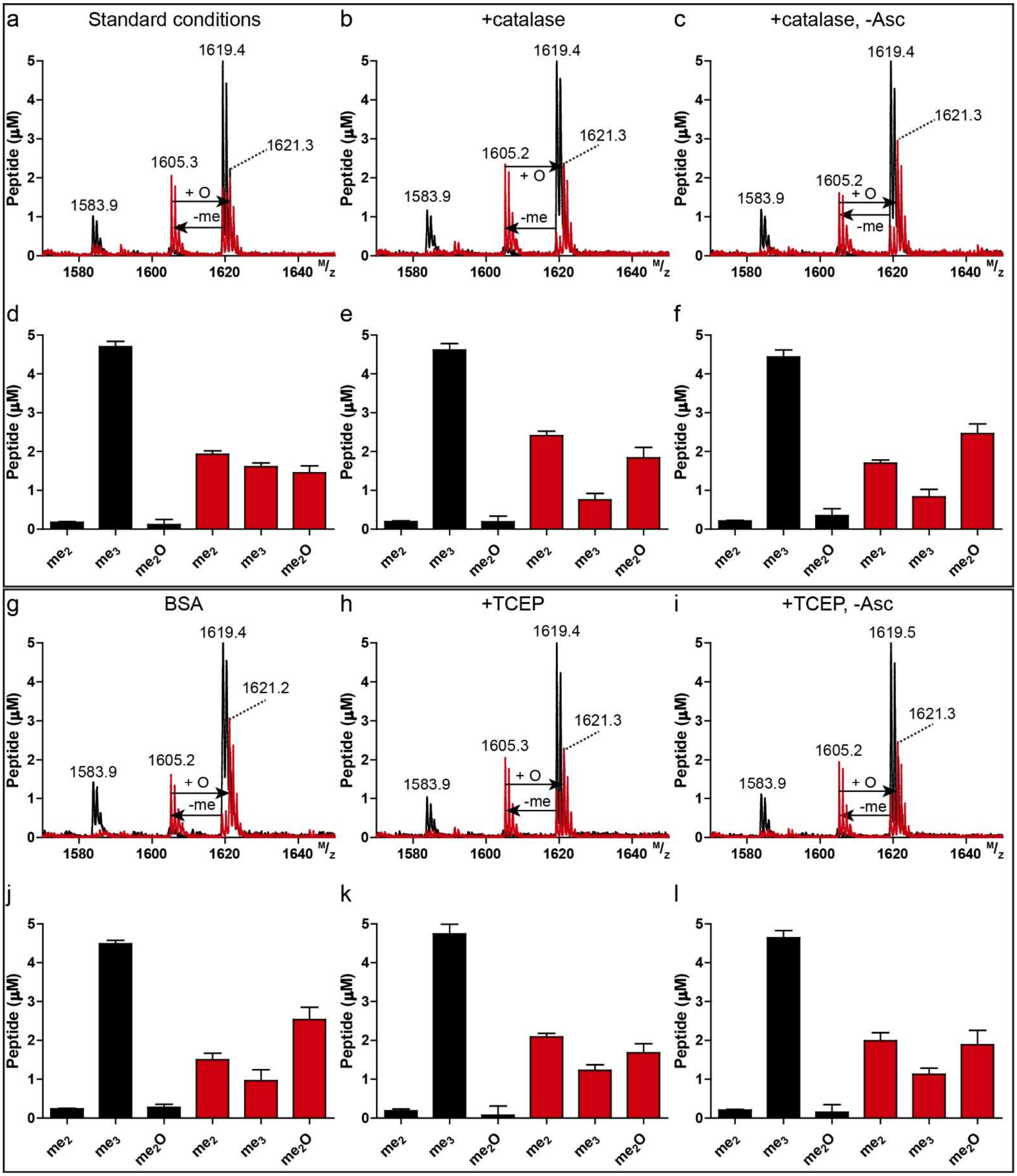


**Supplementary Fig. 13 MALDI-TOF assay of KDM4E$_{JmjC}$ (0.5 µM) catalysed demethylation of H3K$_{P}$9me$_3$ (5.0 µM) under different conditions. a,d** Standard conditions, **b,e** with catalase, **c,f** with catalase and without Asc, **g,j** with BSA, **h,k** with TCEP and **i,l** with TCEP and without Asc. Standard conditions: Asc (500 µM), Fe(II) (50 µM) and 2OG (100 µM), 1 hr. Black t: 0 min, red t: 60 min. Errors are standard deviations of the mean (n = 3–4).

9

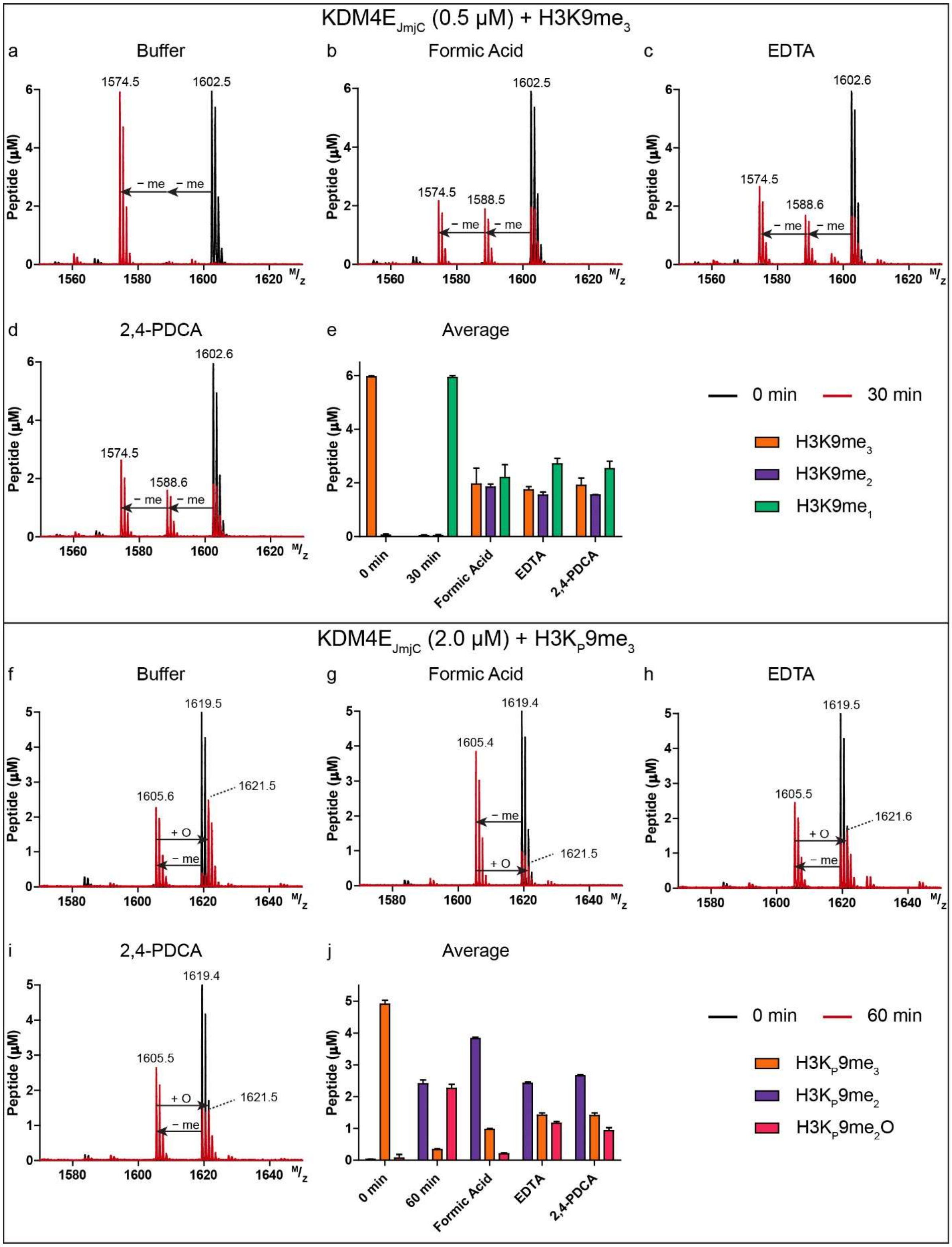


**Supplementary Fig. 14 MALDI-TOF assay of KDM4E$_{JmjC}$ (0.5 µM for a-e, 2.0 µM for f-j) catalysed demethylation of H3K9me$_3$ (6.0 µM, a-e) or H3K$_P$9me$_3$ (5.0 µM, f-j) quenched with different agents.** a-d, f-i Representative mass spectra of H3K9me$_3$ (6.0 µM, a-d) or H3K$_P$9me$_3$ (5.0 µM, f-i) incubated with KDM4E$_{JmjC}$ (0.5 µM for a-e, 2.0 µM for f-i), quenched after 5 min (a-e) or 10 min (f-i) with buffer (a,f) formic acid (1%, b,g), EDTA (5 mM, c,h), or 2,4-pyridinedicarboxylic acid (5 mM, d,i). Reactions were then 'quenched' for a second time (30 min for a-e, 60 min for f-j) with formic acid (1%), then analysed by MALDI-TOF MS. Black t: 0 min, red t: 30 min (a-d) or 60 min (f-i). e,j bar graphs of samples acquired (a-d, f-i). Errors are standard deviations of the mean (n: 3–4). Standard conditions: Asc (500 µM), Fe(II) (50 µM) and 2OG (100 µM), 1 hr.

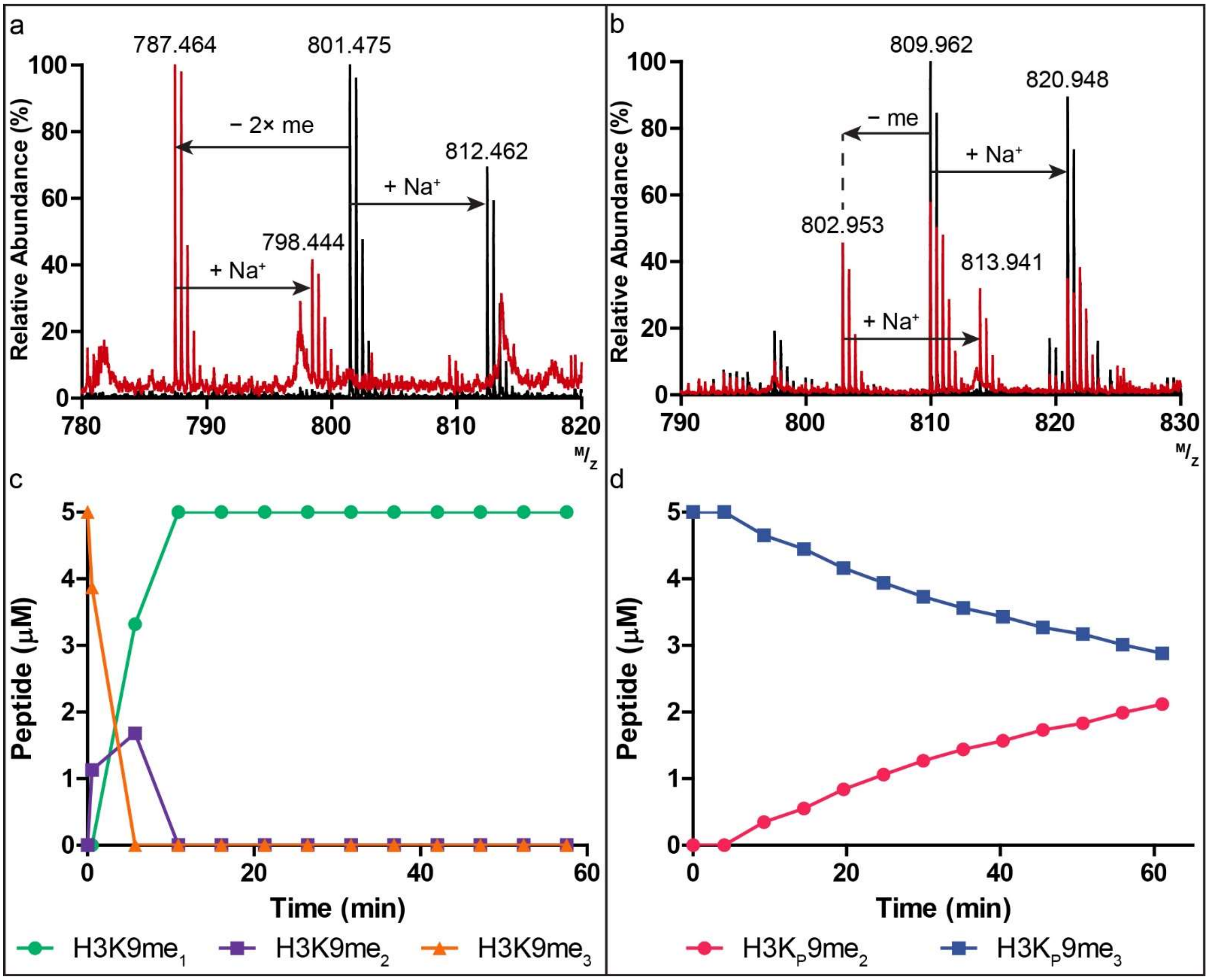


**Supplementary Fig. 15 Time-course of KDM4E$_{JmjC}$ (0.5 µM) catalysed demethylation of H3K9me$_3$ (5.0 µM) or H3K$_P$9me$_3$ (5.0 µM) assayed by LC-MS showing both are substrates.** **a** Mass spectra of H3K9me$_3$, **b** Mass spectra of H3K$_P$9me$_3$, **c** Time-course of substrate H3K9me$_3$ and **d** Time-course of substrate H3K$_P$9me$_3$. Standard conditions: Asc (500 µM), Fe(II) (50 µM) and 2OG (100 µM). **a,b** Black t: 0 min, red t: 66.6 min (**a**) or 70.2 min (**b**).

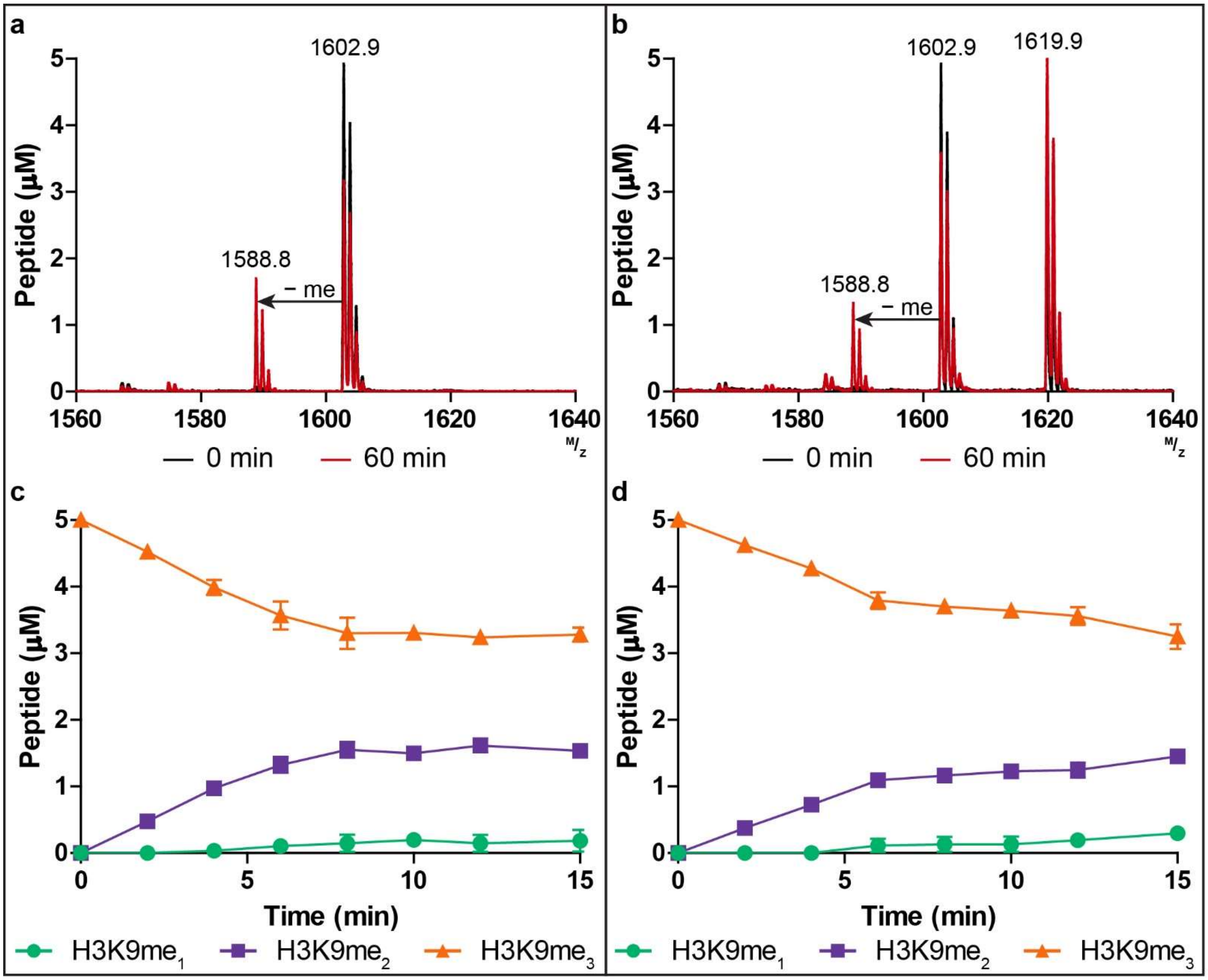


**Supplementary Fig. 16 MALDI-TOF competition assay of KDM4E$_{JmjC}$ (0.10 µM) catalysed demethylation of H3K9me$_3$ (5.0 µM) in competition with H3K$_P$9me$_3$ (5.0 µM).** **a** Mass spectra of control H3K9me$_3$ (5.0 µM), **b** Mass spectra of a competition assay with H3K9me$_3$ (5.0 µM) and H3K$_P$9me$_3$ (5.0 µM), **c** Time-course of H3K9me$_3$, and **d** Time-course of competition assay between H3K9me$_3$ (5.0 µM) and H3K$_P$9me$_3$(5.0 µM). Standard conditions: Asc (500 µM), Fe(II) (50 µM), 2OG (100 µM), 15 min, analysis acquired using MALDI-TOF. Black t: 0 min, red t: 6 min (**a,b**). Errors in the time-course are standard deviations of the mean (n = 2 or 3) (**c,d**).

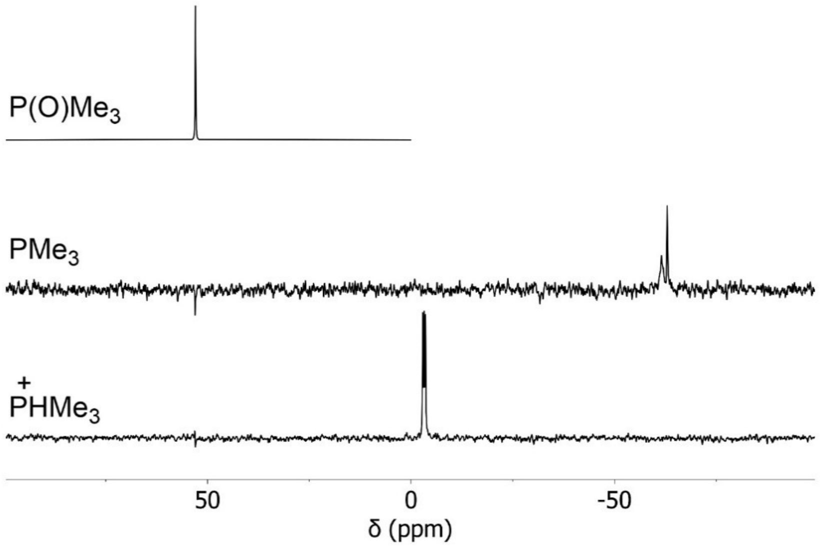


**Supplementary Fig. 17 $^{31}$P NMR analyses of $PMe_3$, $P^+Me_3H$ and $P(O)Me_3$ in $D_2O$.** Analyses of the samples by $^{31}$P NMR show resonances for $P(O)Me_3$, $PMe_3$, $PMe_3H^+$, at $\delta_P$ = 53, -63.0, and -3.3 ppm, respectively.

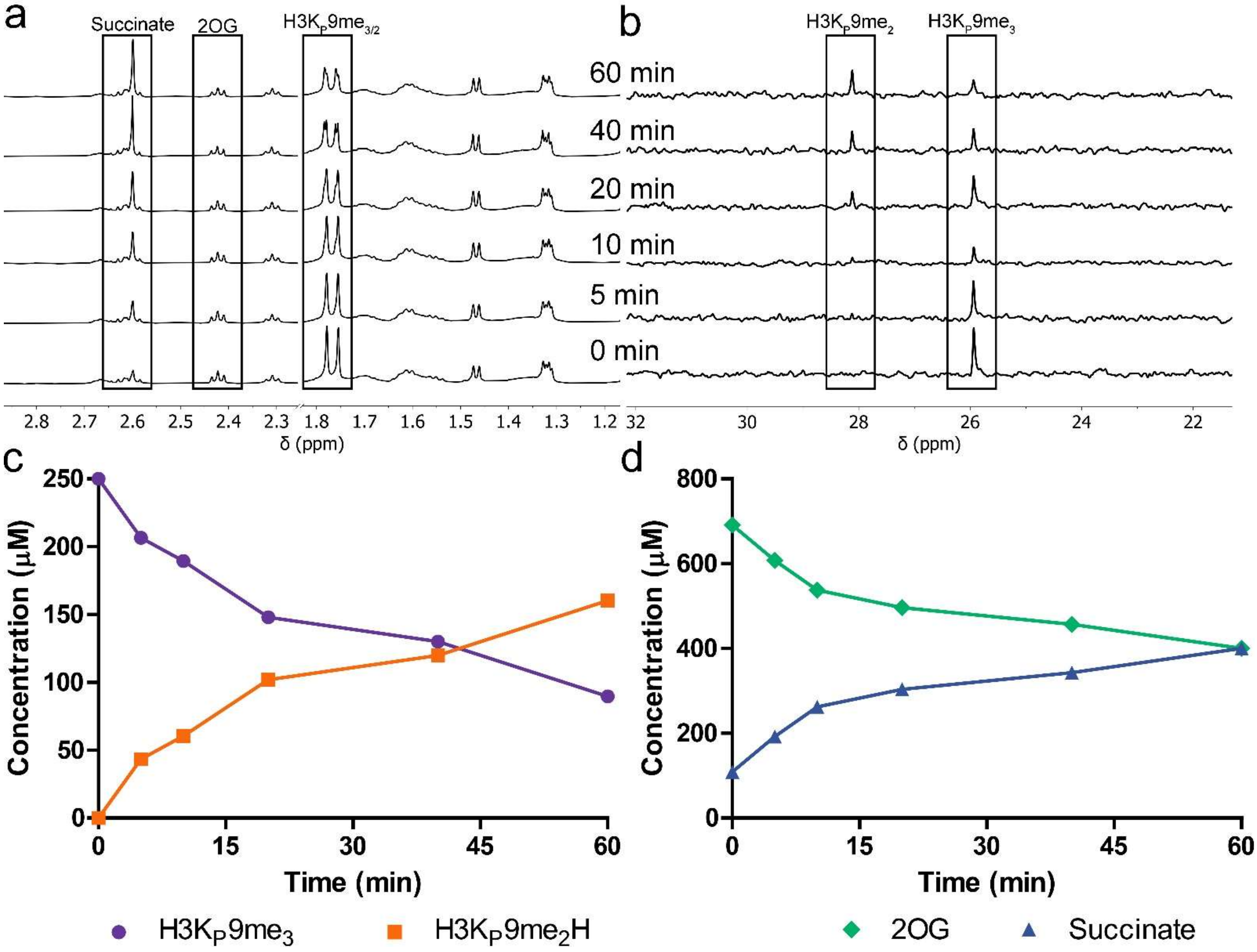


**Supplementary Fig. 18 $^{1}$H and $^{31}$P NMR time-course measurements of KDM4E$_{JmjC}$ catalysed demethylation of H3K$_P$9me$_3$.** **a** $^{1}$H NMR time-course of the incubation of H3K$_P$9me$_3$ (250 μM) with KDM4E$_{JmjC}$ (50 μM), **b** $^{31}$P NMR time-course of the incubation of H3K$_P$9me$_3$ with KDM4E$_{JmjC}$, **c** Plot of the conversion of H3K$_P$9me$_3$ into H3K$_P$9me$_2$H$^+$ based on $^{1}$H NMR data, **d** Plot of the conversion of 2OG into succinate based on $^{1}$H NMR analysis. After quenching the reaction, analyses by $^{31}$P (b) $^{1}$H (a) NMR provided evidence for both H3K$_P$9me$_3$ ($\delta_H$ = 1.75, $\delta_P$ = 25.9 ppm) and H3K$_P$9me$_2$H ($\delta_H$ = 1.74, $\delta_P$ = 28.4 ppm) inside the mixture. Standard conditions: peptide H3K$_P$9me$_3$ (250 μM) was incubated with sodium ascorbate (500 μM) 2-oxoglutarate (500 μM), $Fe(NH_4)_2(SO_4)_2$ (50 μM), and KDM4E (50.0 μM, in HEPES-d$_{18}$ buffer (50 mM, pH 7.5) in $D_2O$ (>95% $^{2}$H) for 1 hour.

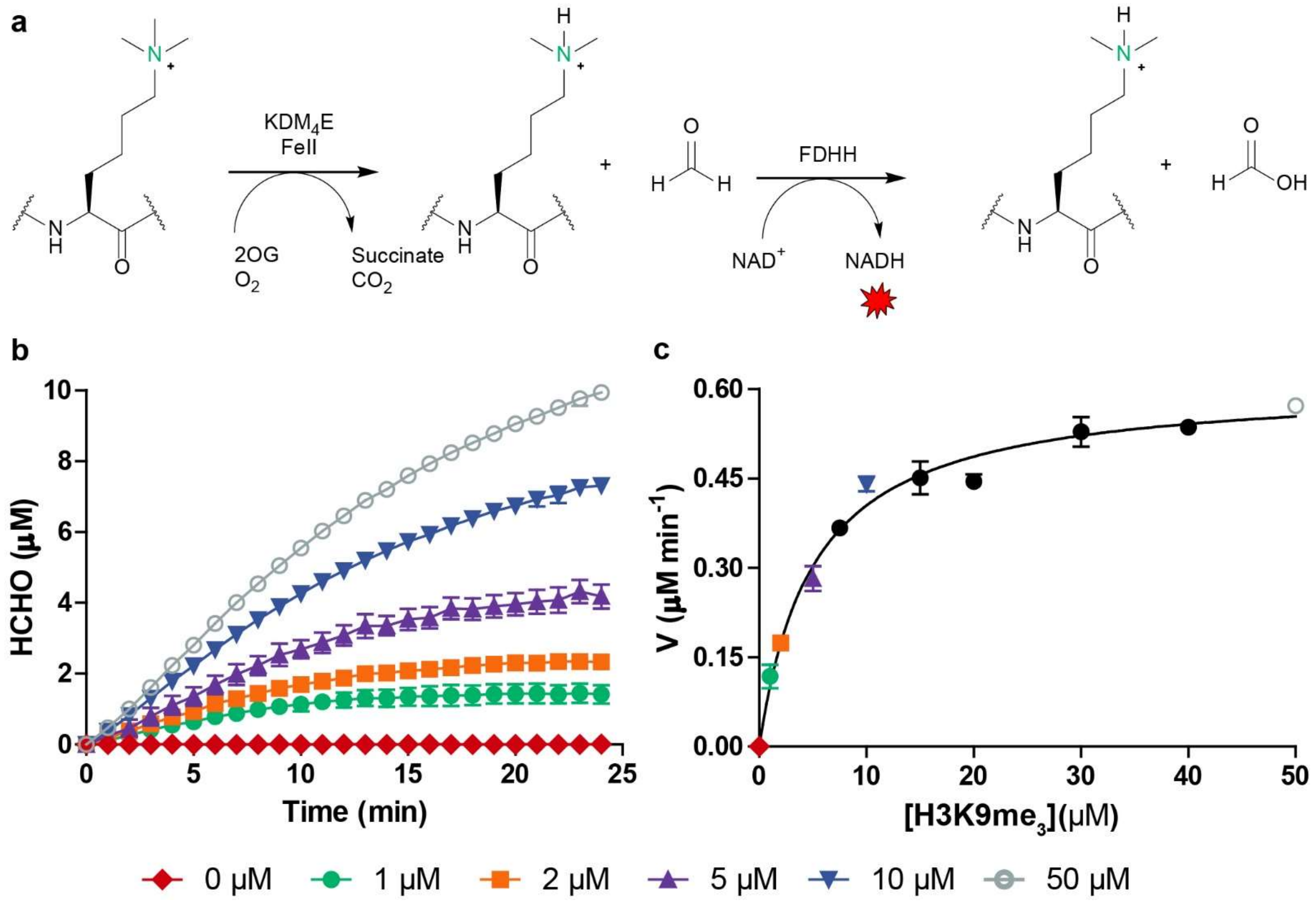


**Supplementary Fig. 19 KDM4E$_{JmjC}$ catalysed demethylation of H3K9me$_3$ coupled to formaldehyde dehydrogenase measurement of the formation of NADH. a** FDH enzyme coupled to KDM4E$_{JmjC}$ demethylation assay measuring the levels of NADH as function of the demethylation. **b** FDH Activity assay with KDM4E$_{JmjC}$ (0.1 µM) and varied concentrations of H3K9me$_3$ (0–50 µM). **c** Michaelis-Menten analysis. Standard conditions: FDH (1.0 µM), Asc (1.0 mM), Fe(II) (50 µM), 2OG (200 µM) and NAD$^+$ (250 µM). Errors are standard deviations of the mean (n = 2 or 3).

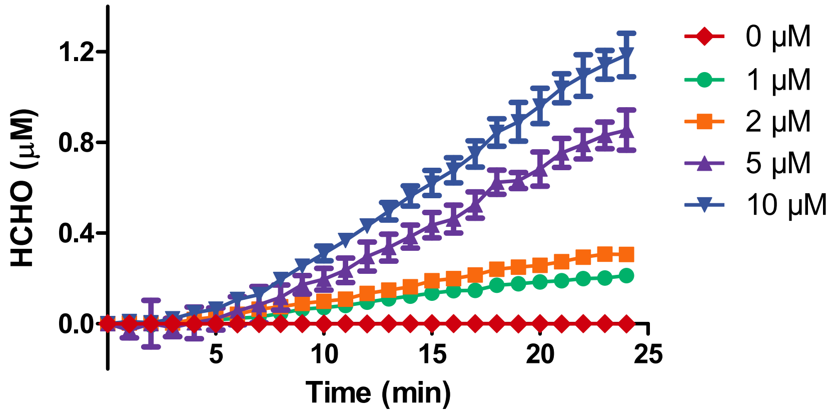


**Supplementary Fig. 20 FDH activity assay of KDM4E$_{JmjC}$ (0–10 μM) catalysed demethylation of H3K$_{P}$9me$_{3}$ (10 μM) demonstrating that KDM4E$_{JmjC}$ can demethylate H3K$_{P}$9me$_{3}$ in an enzyme dependent manner that releases formaldehyde.** Standard conditions: FDH (1.0 μM), Ascorbate (1.0 mM), Fe(II) (50 μM), 2OG (200 μM) and $NAD^{+}$ (250 μM). Errors are standard deviations of the mean (n = 3 or 4).

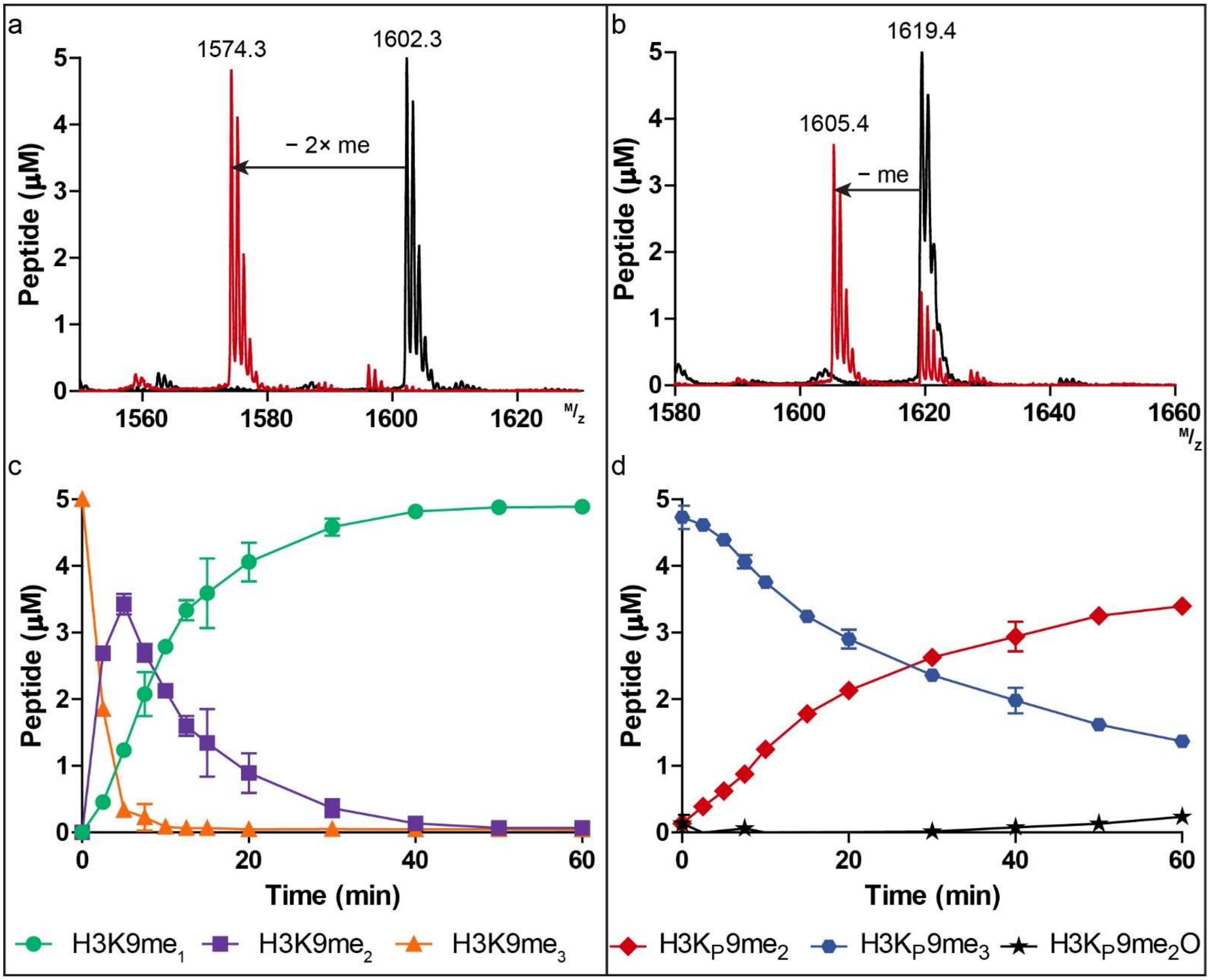


**Supplementary Fig. 21 MALDI-TOF assay of KDM4A$_{JmjC}$ (2.4 µM) catalysed demethylation of H3K9me$_3$ (5.0 µM) or H3K$_P$9me$_3$ (5.0 µM). a** Mass spectra of H3K9me$_3$, **b** Mass spectra of H3K$_P$9me$_3$, **c** Time-course of H3K9me$_3$, and **d** Time-course of H3K$_P$9me$_3$. Standard conditions: Asc (500 µM), Fe(II) (50 µM) and 2OG (100 µM), 1 hr. **a,b** Black t: 0 min, red t: 60 min. **c,d** Data points in the time-course are duplicates with errors as standard deviations of the mean.

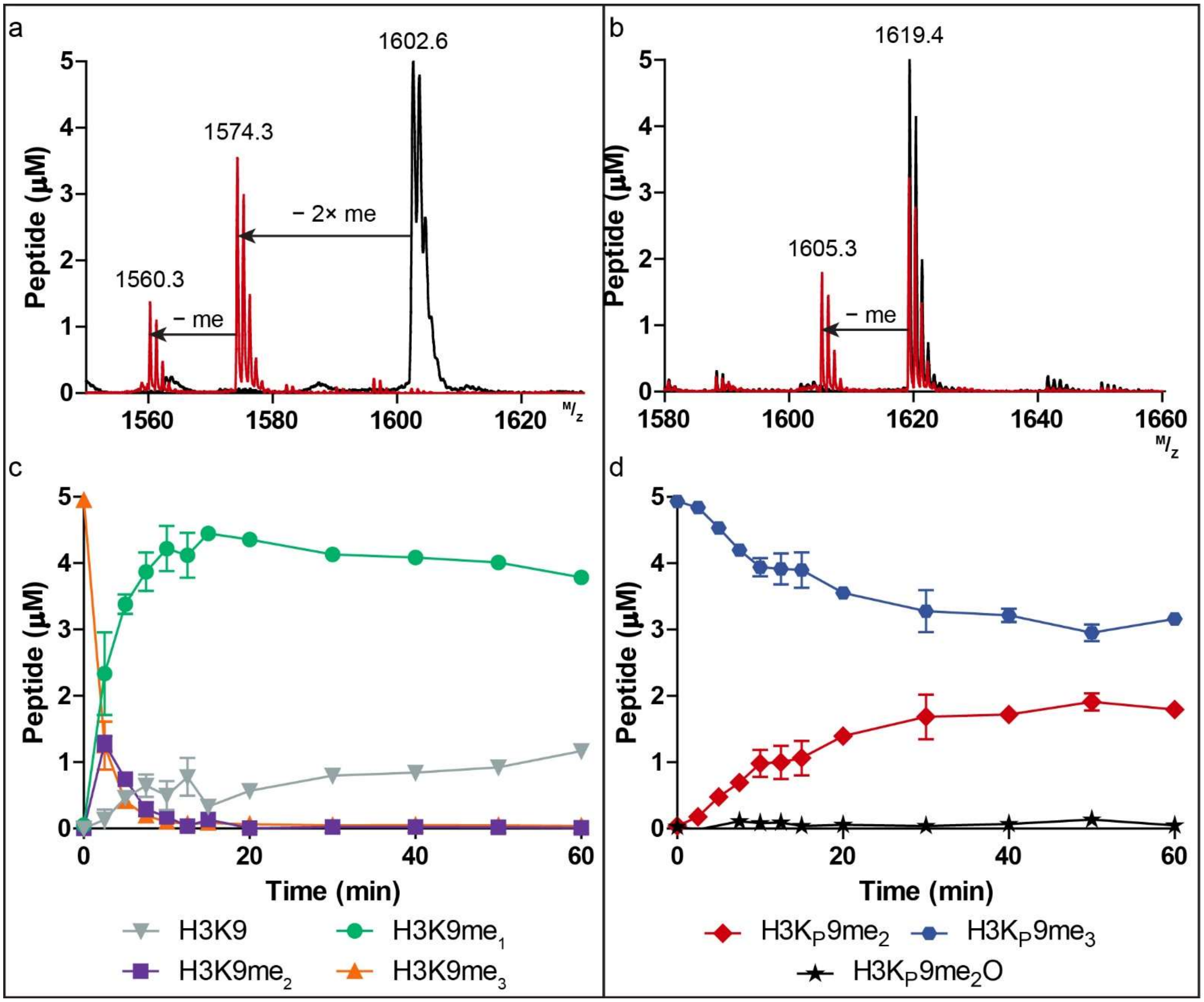


**Supplementary Fig. 22 MALDI-TOF assay of KDM4D$_{JmjC}$ (2.4 µM) catalysed demethylation of H3K9me$_3$ (5.0 µM) or H3K$_P$9me$_3$ (5.0 µM).** **a** Mass spectra of H3K9me$_3$, **b** Mass spectra of H3K$_P$9me$_3$, **c** Time-course of H3K9me$_3$, and **d** Time-course of H3K$_P$9me$_3$. Standard conditions: Asc (500 µM), Fe(II) (50 µM) and 2OG (100 µM), 1 hr. **c,d** Data points in the time-course are duplicates with errors as standard deviations of the mean.

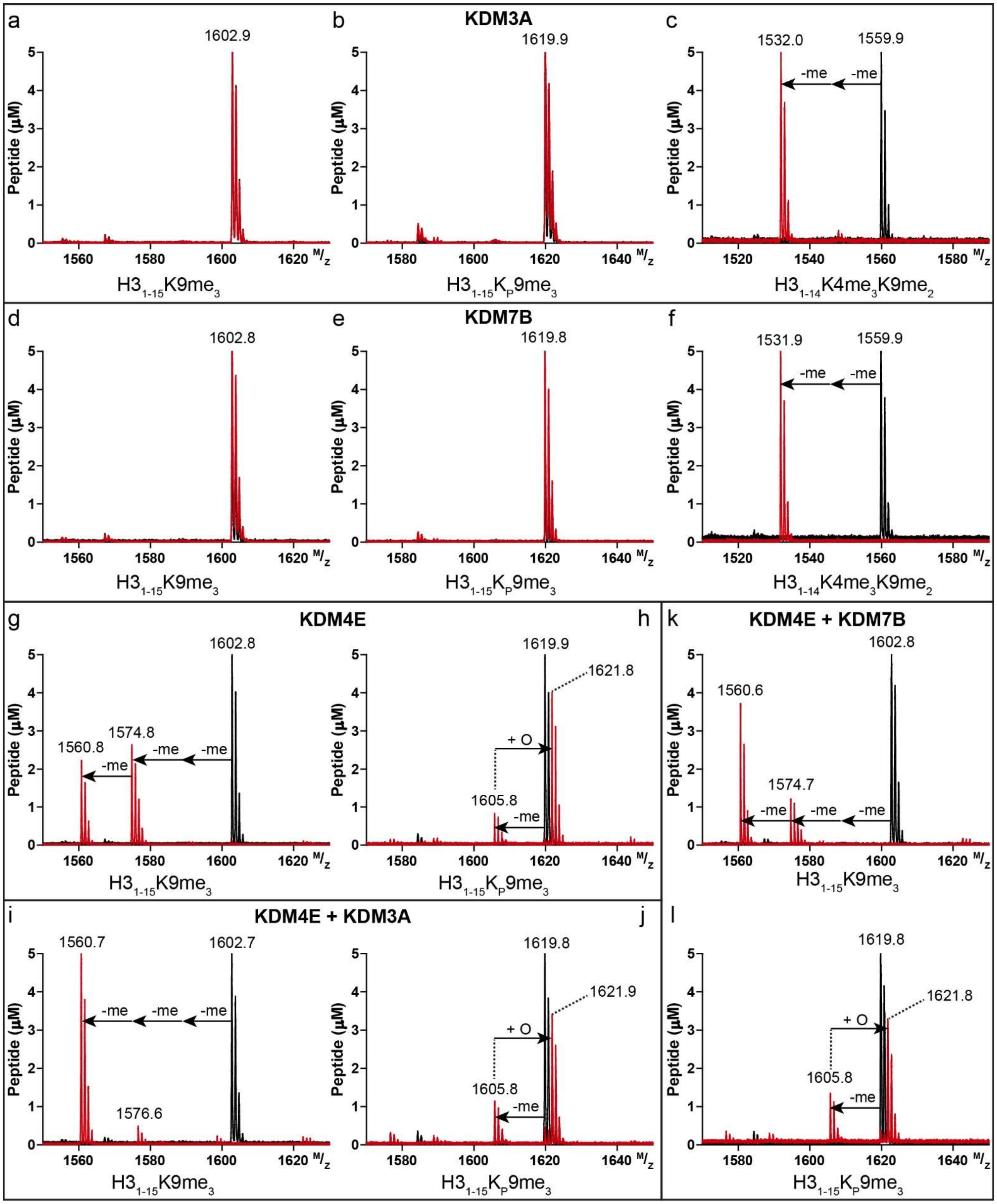


**Supplementary Fig. 23 MALDI-TOF demethylation activity assay of H3$_{1-15}$K9me$_3$ (5.0 µM) and H3$_{1-15}$K$_P$9me$_3$ (5.0 µM) and if applicable H3$_{1-14}$K4me$_3$K9me$_3$ by KDM4E$_{JmjC}$ (2.0 µM), KDM3A$_{JmjC}$ (2.0 µM) and KDM7B$_{JmjC}$ (2.0 µM). Based on these results only KDM4E$_{JmjC}$ can demethylate H3$_{1-15}$K$_P$9me$_3$.** Standard conditions: KDM4E$_{JmjC}$, KDM3A$_{JmjC}$ and / or KDM7B$_{JmjC}$ (2.0 µM), H3 peptide (5.0 µM), Asc (500 µM), Fe(II) (50 µM), 2OG (100 µM) and, TCEP (500 µM), 1 hr. Black t: 0 min, red t: 60 min.

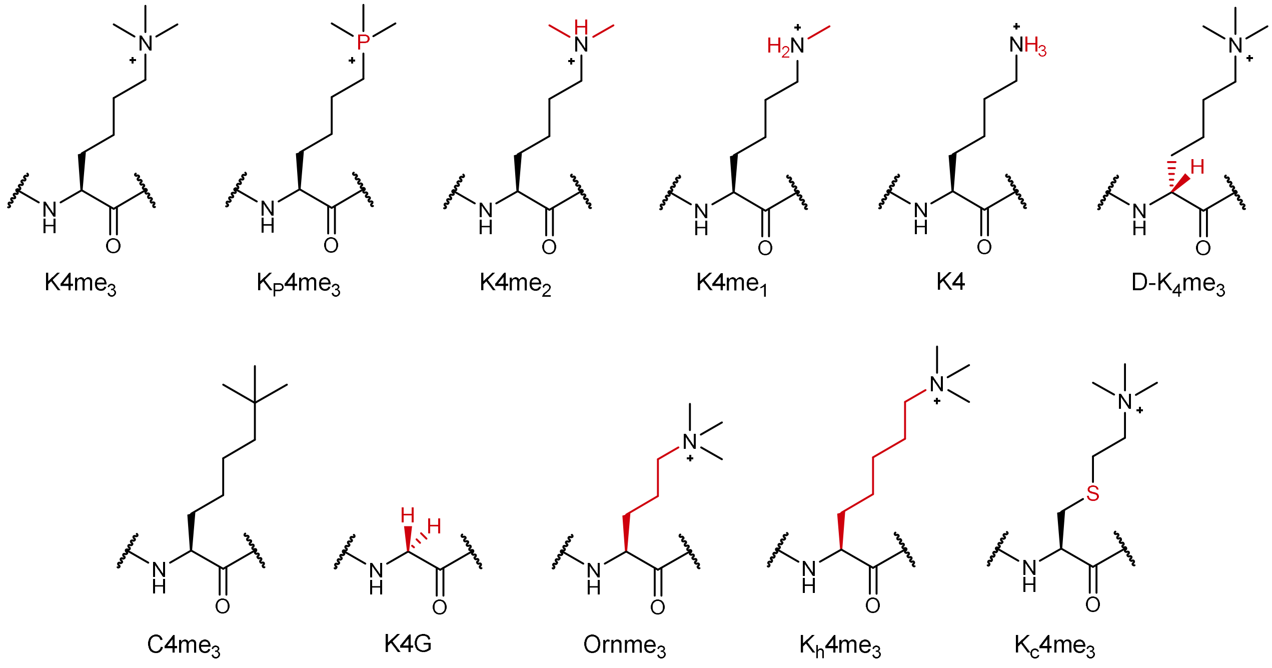


**Supplementary Fig. 24 Structure of lysine analogues and related derivatives used to probe binding in the aromatic cage of chromatin binding modules**[1,2,11,3–10].

**Supplementary Table 5 Overview of reported ITC binding values of chromatin binding modules with H3 peptides ($K_d$ in µM) with various lysine modification.** The corresponding lysine analogue structures are displayed in Fig. 25. **a** Histone H3 1-15 peptides were used for ITC measurements. **b** Tryptophan intrinsic fluorescence assay using histone H3 1-17. **c** Histone H3 1-11 peptide was used for ITC measurements. **d** Values acquired using surface plasmon resonance with histone H3 substrate H3 1-10 - GGG-Biotin. N.D. not determined; N.M: not measured.

| Modification | $KDM5A_{PHD3}$ | $TAF3_{PHD}$ | $BPTF_{PHD}$ | $SGF29_{TTD}$ | $KDM4A_{TTD}$ | Substrate | Publication |
|---|---|---|---|---|---|---|---|
| $K4me_3$ | 0.52 | 0.11 | 1.4 | 3.2 | 3.5 | $H3_{1-10}$ | This paper |
| $K_P4me_3$ | 0.2 | 0.048 | 0.2 | 2.8 | 0.3 | $H3_{1-10}$ | |
| $K4me_3$ | 0.75[a] | 0.17[a], 0.16[b] | 2.7[a] | 0.5[c] | 0.5[a], 10[d] | $H3_{1-10}$ | $KDM5A_{PHD3}$: Wang *et al.*[1]; $BPTF_{PHD}$: Li *et al.*[2]; $TAF3_{PHD3}$: Zhao *et al.*[3], Vermeulen *et al.*[4]; $SGF29_{TTD}$ Bian *et al.*[5]; $KDM4A_{TTD}$: Lee *et al.*[6], Huang *et al.*[7] |
| $K4me_2$ | 0.93[a] | 1.7[b] | 5.0[a] | 1.0[c] | N.M. | $H3_{1-10}$ | |
| $K4me_1$ | 2.79[a] | >200[b] | N.D.[a] | 4.0[c] | N.M. | $H3_{1-10}$ | |
| K4 | 20.7[a] | >200[b] | N.D.[a] | 24[c] | N.D.[d] | $H3_{1-10}$ | |
| L-$K4me_3$ | 0.11 | 0.082 | 0.44 | 1.7 | 1.1 | $H3_{1-10}$ | Belle *et al.*[8] |
| D-$K4me_3$ | 1.2 | 0.73 | 3.8 | 50 | 39 | $H3_{1-10}$ | |
| $K4me_3$ | 0.094 | 0.024 | 0.49 | 1.7 | 0.94 | $H3_{1-10}$ | Kamps *et al.*[9] |
| $K_C4me_3$ | 0.34 | 0.79 | 0.76 | 1.4 | 16 | $H3_{1-10}$ | |
| K4G | 88 | 36 | N.M. | N.M. | N.M. | $H3_{1-10}$ | |
| $K4me_3$ | 0.52 | 0.082 | 1.4 | 3.1 | 4.0 | $H3_{1-10}$ | Al Temimi *et al.*[10] |
| $Ornme_3$ | 10 | 1.0 | 18 | 19 | 15 | $H3_{1-10}$ | |
| $K_h4me_3$ | 0.36 | 0.41 | 3.1 | 7.3 | 5.7 | $H3_{1-10}$ | |
| $K4me_3$ | 0.071 | 0.084 | 1.9 | 2.6 | 6.6 | $H3_{1-10}$ | Hintzen *et al.*[11] |
| $K4Cme_3$ | 0.15 | 0.042 | 3.8 | 6.1 | 3.1 | $H3_{1-10}$ | |

**Supplementary Table 6 Benchmark analysis of binding energies (in kT) of methylated ammonium ions with benzene.**

| | BLYP-D3BJ/TZ2P | PBE0-D4/TZ2P | LNO-CCSD(T)/CBS[a] |
|---|---|---|---|
| $Me_0$ | -30.9 | -31.0 | -30.8 |
| $Me_1$ | -31.0 | -30.5 | -29.2 |
| $Me_2$ | -29.4 | -29.3 | -28.5 |
| $Me_3$ | -27.4 | -27.8 | -27.5 |
| $Me_4$ | -17.0 | -16.9 | -15.8 |

[a] Reported values by Rahman *et al.*[12]

# 2 Supplementary Methods

## 2.1 General experimental conditions

Reactions were carried out in oven dried round-bottomed flasks with magnetic stirring in a fume-hood, unless stated otherwise. Anhydrous solvents, reagents, and starting materials were obtained from commercial sources and used without further purification. Formic acid for demethylation assays were obtained from Fisher scientific. Alternatively, anhydrous solvents were obtained by purification of HPLC grade solvents over activated alumina column using an MBraun SPS800 solvent purification system. When stated, sparging of solvents was performed by passing gaseous $N_2$ through the solution for a period of at least 30 minutes prior to use. Reaction progress was monitored by thin layer chromatography (TLC) using Merck silica gel 60 F254 coated on aluminium foil or glass TLC plates (TLC Silica gel 60G, F254, Merck, Germany) and visualised using UV light (254 nm), or by staining with ninhydrin or permanganate. Progress was also monitored using a liquid chromatography − mass spectrometry (LC−MS) system (Agilent Technologies 1260 Infinity Series, equipped with a 6120 Quadrupole mass spectrometer and optionally run over a Merck Chromolith Performance C18 (100 × 4.2 mm) HPLC column). Chromatographic purification of synthetic compounds was performed using Silica gel, Merck™ grade (pore size 60 Å; particle size 230-400 mesh, 40-63 µm), or alternatively, using a Biotage Isolera flash purification system, fitted with Biotage prepacked SNAP KPSil or SNAP-ULTRA columns and analytical grade solvents. NMR spectra were recorded using a Varian Inova 400 equipped with a 5 mm PFG Indirect Detection Probe, a Bruker Avance III 500 MHz equipped with a BBFO $N_2$ Prodigy CryoProbe, a Bruker DMX300 equipped with a QNP-probe, or a Bruker AVIII HD 600 equipped with a BB-F/H $N_2$ Prodigy CryoProbe. NMR samples were prepared in 5 mm tubes, and recorded at 298 K, unless stated otherwise. Coupling constants (*J*) are reported to the nearest 0.5 Hz. Chemical shifts (δ) are reported in parts per million (ppm) and referenced to their respective solvent resonance. Multiplicities are reported as singlet (s), doublet (d), triplet (t), multiplet (m), doublet of doublet (dd), broad singlet (br s). Infrared (IR) spectra were recorded using a Bruker Tensor 27 instrument and reported in wavenumbers ($cm^{-1}$). Optical rotation, $[\alpha]_D^T$ in $10^{-1}$ deg $cm^2$ $g^{-1}$, values were obtained using a PerkinElmer 341 polarimeter and corresponding samples were prepared in analytical grade solvents with concentrations reported in g 100 $mL^{-1}$. When specified, purification of products was carried out by preparative HPLC using a Shimadzu LC 20AT, equipped with a Phenomenex Gemini NX (particle size: 10 µm, pore size: 110 Å, C18) in conjunction with a Shimadzu SPD 20A deuterium lamp at 215 and 254 nm as detector.

# 2.2 Synthesis of Fmoc-Lys(Phosme$_3$)-OH

**Synthesis of (*S*)-6-(((benzyloxy)carbonly)amino)-2-((*tert*-butoxycarbonly)amino)hexanoic acid 1**

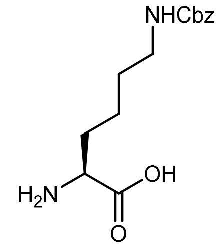


*The preparation of* ***1*** *was performed according to the procedure of Bambal et al*[13]. To aqueous NaOH (50.0 mL 0.200 M) were added L-lysine mono HCl salt (2.00 g, 11.0 mmol, 1.00 equivalent) and $CuSO_4$ (1.03 g, 6.40 mmol, 0.600 equivalent). After stirring for 2 hours, the pH was adjusted to pH 9, using aqueous $NaHCO_3$ (1.85 g, 22.0 mmol, 2.00 equivalents) and aqueous NaOH (1.00 M) solution. Benzyl chloroformate (2.30 mL, 16.0 mmol, 1.50 equivalents) was then added over 5 minutes. The blue solution was stirred for 24 hours, after which solid was collected by filtration. The residue was washed with $H_2O$ (40 mL). The solid filter cake was redissolved in an aqueous $Na_2$EDTA (300 mL, saturated) solution and stirred overnight. The white solid was collected by filtration and used as a crude product in the next step.

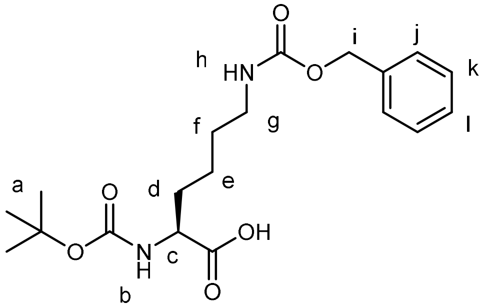


The crude product was suspended in 1,4-dioxane : $H_2O$ (120 mL, 1 : 1); the pH was adjusted to 10 with aqueous NaOH (1.00 M). $Boc_2O$ (2.63 g, 12.1 mmol, 1.10 equivalents) was then added; the resulting suspension was stirred overnight. The reaction was quenched by addition of aqueous $KHSO_4$ (50 mL, 1 M), and extracted with $Et_2O$ (3 × 75 mL). The combined ether layers were washed with brine (100 mL), dried over $Na_2SO_4$, filtered, and concentrated under reduced pressure. The crude product was purified using column chromatography ($SiO_2$, MeOH with 0.1% (v/v) AcOH in $CH_2Cl_2$, 3 – 5%): the desired compound was obtained as a viscous colourless oil (1.97 g, 5.18 mmol, 47 %). FT-IR $\nu_{max}$ (cm$^{-1}$): 3341, 2974, 2931, 1705, 1533, 1424, 1392, 1367, 1242, 1159, 1052, 1023. $^1$H NMR (400 MHz, $CDCl_3$) δ: 7.41 – 7.29 (m, 5H, $H_j$ and $H_k$ and $H_l$), 6.32 – 6.27 (m, 1H, $H_h$), 5.27 (d, $J$ = 8.0 Hz, 1H, $H_b$), 5.09 (s, 2H, $H_i$), 4.29 (s, 1H, $H_c$), 3.20 (t, $J$ = 6.0 Hz, 2H, $H_g$), 1.74 – 1.62 (m, 2H, $H_d$), 1.60 – 1.48 (m, 2H, $H_f$), 1.44 (s, 9H, $H_a$), 1.43 – 135 (m, 2H, $H_e$). $^{13}$C NMR (126 MHz, $CDCl_3$) δ: 176.2, 156.7, 155.9, 136.5, 128.5,128.2, 128.2, 80.3, 66.8, 53.2, 40.5, 31.8, 29.4, 28.3, 22.3. LC-MS analysis $t_r$: 16.15 MS r/e (rel. intensity): 403 (100, [M + Na]$^+$), 381 (23, [M+H]$^+$). The analytical data are in agreement with reported values[13].

**Synthesis of *(S)-tert*-butyl 6-(((benzyloxy)carbonyl)amino)-2-((*tert*-butoxycarbonyl)amino)hexanoate 2**

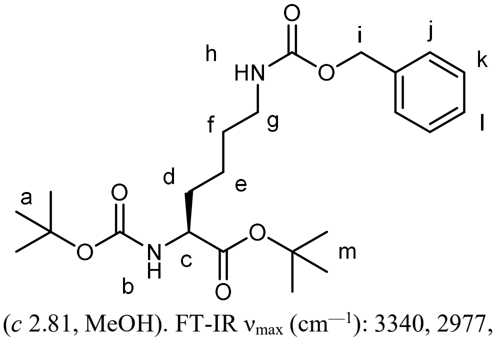


*The preparation of* ***2*** *was performed according to the procedure of Strazzolini et al*[14]. Acid **1** (1.48 g, 3.90 mmol, 1 equivalent) was dissolved in $^t$BuOH (40 mL) and warmed to 30 °C, while stirring. *N,N*-Dimethyl-4-aminopyridine (143 mg, 1.16 mmol, 0.30 equivalents), and $Boc_2O$ (1.03 g, 4.68 mmol, 1.20 equivalents) were then added. The resulting mixture was stirred for 20 hours. The solvent was then removed under reduced pressure. The crude yellow oil was purified by column chromatography ($SiO_2$, EtOAc in *n*-heptane 30 – 40%) after which the desired compound was obtained as an oil (1.48 g, 3.40 mmol, 87%). $[\alpha]^{25}_D$ – 16.5 (*c* 2.81, MeOH). FT-IR $\nu_{max}$ (cm$^{-1}$): 3340, 2977, 2934, 1702, 1524, 1456, 1392, 1367, 1249, 1153, 1047, 1024. $^1$H NMR (400 MHz, $CDCl_3$) δ: 7.40 – 7.28 (m, 5H, $H_j$ and $H_k$ and $H_l$), 5.08 (d, $J$ = 9.0 Hz, 2H, $H_i$), 4.81 (br s, 1H, $H_h$), 4.21 – 4.09 (m, 1H, $H_c$), 3.19 (dt, $J$ = 13.0, 6.5 Hz, 2H, $H_g$), 1.76 (d, $J$ = 9.5 Hz, 2H, $H_d$), 1.66 – 1.51 (m, 2H, $H_f$), 1.45 (s, 9H, $H_a$ or $H_m$), 1.43 (s, 9H, $H_a$ or $H_m$), 1.43 – 1.35 (m, 2H, $H_e$). $^{13}$C NMR (101 MHz, $CDCl_3$) δ: 171.9, 156.4, 155.5, 136.6, 128.5, 128.1, 128.1, 81.8, 79.6, 66.6, 53.6, 40.8, 32.6, 29.4, 28.3, 28.0, 22.3. LC-MS analysis: $t_r$ = 19.46 MS r/e 459.4 (100, [M+Na]$^+$), 437 (12, [M+H]$^+$). HR ESI-MS: 437.2640, ([M+H]$^+$) $C_{23}H_{37}N_2O_6$, calc: 437.2646. The analytical data are in agreement with reported values [15,16].

**Synthesis of *(S)-tert*-butyl 6-amino-2-((*tert*-butoxycarbonyl)amino)hexanoate 3**

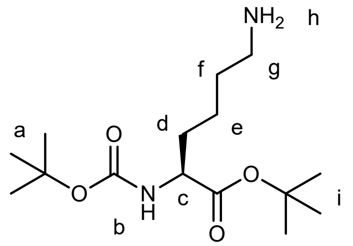


*The preration of **3** was performed according to the procedure of Fekner et al*[15]. A solution of ester **2** (500 mg, 1.15 mmol, 1.00 equivalent) in MeOH (40 mL), was mixed with Pd/C (70.3 mg, 14 w/w%). The mixture was placed under an $H_2$ atmosphere and stirred for 24 hours. The black suspension was filtered through Celite® and washed with MeOH (5 × 40 mL). The filtrate was concentrated under reduced pressure, affording the desired compound as a brown oil (304 mg, 1.01 mmol, 88%). The crude product was used in the next step without further purification. $[\alpha]^{20}_D$ + 6.8 (*c* 1.00, $CHCl_3$). $^1$H NMR (400 MHz, $CDCl_3$) δ: 5.04 (d, *J* = 8.0 Hz, 1H, $H_b$), 4.17 (q, *J* = 5.5 Hz, 1H, $H_c$), 2.71 (t, *J* = 6.5 Hz, 2H, $H_g$), 1.67 – 1.56 (m, 2H, $H_d$), 1.53 – 1.47 (m, 2H, $H_f$), 1.47 – 1.42 (m, 18H, $H_a$ and $H_i$), 1.39 – 1.17 (m, 2H, $H_e$). $^{13}$C NMR (126 MHz, $CDCl_3$) δ: 172.1, 155.4, 81.7, 79.6, 53.9, 41.9, 33.3, 32.8, 28.3, 28.0, 22.5. LC-MS analysis: $t_r$ = 13.30 MS r/e (rel. intensity): 303 (100, $[M+H]^+$). HR ESI-MS: 303.2275 ($[M+H^+]$) $C_{15}H_{31}N_2O_4$, calc. 303.2278, found. The analytical data are in agreement with reported values[15,16].

**Synthesis of *(S)-tert*-butyl 2-((*tert*-butoxycarbonyl)amino)-6-hydroxyhexanoate 4**

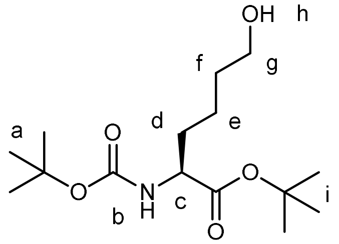


*An adaptation of the procedure reported by Bambal et al. was used for the preparation of **4***[17]. A solution of amine **3** (192 mg, 0.64 mmol, 1 equivalent) in 1,4-dioxane (10 mL) was diluted with aqueous borate buffer (40 mL, 0.2 M, pH 9.50). After heating the solution to 60 °C, $Na_2[Fe(CN)_5NO]$ (390 mg, 2.02 mmol, 2.02 equivalents) was added to give a clear red solution. The resulting solution was stirred for 24 hours, quenched with aqueous $KHSO_4$ (20 mL, 1.0 M) solution. The aqueous solution was then extracted with EtOAc (3× 50 mL). The combined organic extracts were washed with brine (100 mL), dried over $Na_2SO_4$, filtered, and concentrated under reduced pressure. Purification by silica column chromatography ($SiO_2$, EtOAc in *n*-heptane 30 – 50%): the desired compound was obtained as an oil (95 mg, 0.31 mmol, 52 %). $[\alpha]^{25}_D$ – 23.7 (*c* 1.37, MeOH). FT-IR $\nu_{max}$ ($cm^{-1}$): 3369, 1712, 1367, 1154. $^1$H NMR (400 MHz, $CDCl_3$) δ: 5.06 (d, *J* = 8.0 Hz, 1H, $H_b$), 4.21 – 4.12 (m, 1H, $H_c$), 3.70 – 3.58 (m, 2H, $H_g$), 1.87 – 1.74 (m, 1H, $H_h$), 1.70 – 1.51 (m, 4H, $H_d$ and $H_f$), 1.47 (s, 9H, $H_a$ or $H_i$), 1.44 (s, 9H, $H_a$ or $H_i$), 1.33 – 1.16 (m, 2H, $H_e$). $^{13}$C NMR (126 MHz, $CDCl_3$) δ: 172.0, 155.5, 81.8, 79.6, 62.5, 53.8, 32.8, 32.2, 28.3, 28.0, 21.4. LC-MS analysis: $t_r$ = 16.31 MS r/e (rel. intensity): 326 (100, $[M+Na]^+$), 303 (8, $[M+H]^+$). HR ESI-MS: 326.19443 ($[M+Na]^+$) $C_{15}H_{29}NO_5Na$, calc. 326.1943. The analytical data are in agreement with reported values[18].·

**Synthesis of *(S)*-tert-butyl 2-((tert-butoxycarbonyl)amino)-6-((methylsulfonyl)oxy)hexanoate 5**

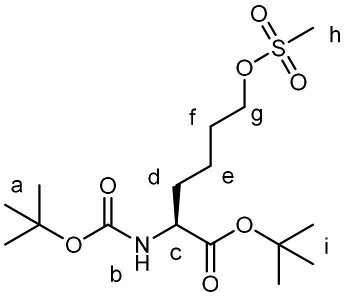


*An adaptation of the procedure reported by Jeon et al. was used for the preparation of **5***[19]. To a solution of alcohol **4** (116 mg, 0.38 mmol, 1 equivalent) in dried $CH_2Cl_2$ (20 mL), were added $Et_3N$ (220 µL, 1.58 mmol, 4.10 equivalents) and methanesulfonyl chloride (61 µL, 0.77 mmol, 2.0 equivalents) under a $N_2$ atmosphere. After 3 hours the stirring was stopped; the solution was then washed $H_2O$ (3 × 25 mL), with $NaHCO_3$ (2 × 25 mL, 1 : 4 saturated $NaHCO_3$ solution : $H_2O$), brine (25 mL), before drying over $Na_2SO_4$. After filtration the solvent was removed under reduced pressure. The crude product was purified using silica column chromatography ($SiO_2$, EtOAc in *n*-heptane 20 to 50%), affording **5** (107 mg, 0.280 mmol, 74 %) as a clear colourless oil. $[\alpha]^{25}_D$ + 8.5 (*c* 0.96, $CHCl_3$). FT-IR $v_{max}$ ($cm^{-1}$): 3390, 2936, 1712, 1508, 1355, 1174, 1154, 930, 846. $^1$H NMR (400 MHz, $CDCl_3$) δ: 5.05 (d, *J* = 8.0 Hz, 1H, $H_b$), 4.25 – 4.20 (m, 2H, $H_g$), 4.20 – 4.14 (m, 1H, $H_c$), 3.00 (s, 3H, $H_h$), 1.88 – 1.70 (m, 2H, $H_d$), 1.68 – 1.55 (m, 2H, $H_f$), 1.55 – 1.46 (m, 2H, $H_e$) 1.48 (s, 9H, $H_a$ or $H_i$), 1.45 (s, 9H, $H_a$ or $H_i$). $^{13}$C NMR (101 MHz, $CDCl_3$) δ: 171.7, 155.4, 82.1, 79.8, 69.6,

53.6, 37.4, 32.4, 28.7, 28.3, 28.0, 21.2. LC-MS analysis: $t_r$ = 17.70 MS r/e (rel. intensity): 404 (100, $[M+Na]^+$). HR ESI-MS: 404.1722 ($[M+Na]^+$) $C_{16}H_{31}NO_7SNa$, calc 404.1719.

**Synthesis of *(S)*-(6-(*tert*-butoxy)-5-((*tert*-butoxycarbonyl)amino)-6-oxohexyl)trimethylphosphonium Mesylate 6**

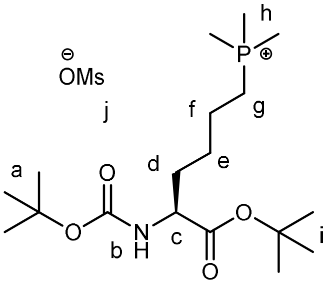


Crude **5** (420 mg 1.10 mmol, 1 equivalent) was dissolved in a trimethylphosphine (2.4 mL, 2.4 mmol, 2.2 equivalents, 1 M in toluene) under an Ar atmosphere. The mixture was reacted in a microwave oven (300 W, 150 °C for 2 hours), then concentrated under reduced pressure. The clear colourless oil was used in the next step without further purification. $[\alpha]^{25}_D$ – 9.7 (*c* 1.15, MeOH). FT-IR $\nu_{max}$ ($cm^{-1}$): 3437, 2979, 2921, 1714, 1524, 1367, 1304, 1173, 1043, 982. $^1H$ NMR (400 MHz, $CDCl_3$) δ: 5.12 (m, 1H, $H_b$), 3.97 –3.91 (m, 1H, $H_c$), 2.56 (s, 3H, $H_j$) 2.19 (m, 2H, $H_g$), 1.91 (s, 9H, $H_h$), 1.57 –1.48 (m, 4H, $H_d$ and $H_f$), 1.33 (s, 11H, $H_e$ and $H_a$ or $H_i$), 1.31 (s, 9H, $H_a$ or $H_i$). $^{13}C$ NMR (101 MHz, $CDCl_3$) δ: 171.7, 155.6, 81.8, 79.5, 53.6, 39.6, 31.7, 28.3, 27.9, 26.4 (d, $J_{C\text{-}P}$ = 16.5 Hz), 23.1 (d, $J_{C\text{-}P}$ = 53.0 Hz), 20.9 (d, $J_{C\text{-}P}$ = 4.5 Hz), 8.0 (d, $J_{C\text{-}P}$ = 54.0 Hz). $^{31}P$ NMR (121 MHz, $CDCl_3$) δ: 26.47 (m, 1P). LC-MS analysis $t_r$: 13.68 MS r/e 362 (100, M). HR ESI-MS: 362.2459 ($[M]^+$) $C_{18}H_{37}NO_4P$, calc. 362.2460.

**Synthesis of *(S)*-(5-(((9*H*-fluoren-9-yl)methoxy)carbonyl)amino)-6-(trimethylphosphonio)hexanoate 7**

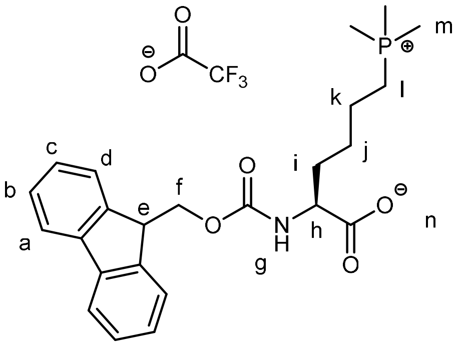


*An adaptation of the procedure by Kamps et al. was used for the preparation of 7*[9]*.* To a round bottom flask was added Boc protected **6** (100 mg, 0.22 mmol, 1 equivalent) and $CF_3COOH$ (TFA, 5 mL). After stirring for 2 hours the solvent was removed under reduced pressure. The crude oil was then redissolved in a mixture of 1,4-dioxane : $H_2O$ (40 mL, 1 : 1). To this solution was added $K_2CO_3$ (423 mg, 3.06 mmol, 14.0 equivalents), adjusting the pH to 8/ 9. Fmoc-OSu (81 mg, 0.24 mmol, 1.1 equivalents) was then added and the resulting suspension stirred for 16 hours. The solvent was then removed under reduced pressure. The resultant crude oil was purified by preparative HPLC. Injections of the crude product in MeCN : $H_2O$ (1 : 2) 1 mL at a time, 10 mg/mL. Typical conditions per run: 67 bar, mobile phase; starting with 5% MeCN and 95% $H_2O$, reaching 80% MeCN in $H_2O$ over 18 minutes, maintained for 2 minutes, subsequently increasing to 100% MeCN over 5 minutes. The desired compound was obtained as a viscous clear oil (60.1 mg, 0.111 mmol, 51%). $[\alpha]^{25}_D$ + 5.2 (*c* 1.1, MeOH). FT-IR $\nu_{max}$ ($cm^{-1}$): 3018, 2922, 1689, 1536, 1450, 1304, 1200, 1128, 981, 762, 742. LC-MS analysis $t_r$: 9.81 MS r/e 428 (100, M). $^1H$ NMR (400 MHz, $CD_3OD$) δ: 7.76 (d, $J$ = 7.5 Hz, 2H, $H_a$), 7.64 (d, $J$ = 7.5 Hz, 2H, $H_d$), 7.36 (t, $J$ = 7.5 Hz, 2H, $H_b$), 7.28 (t, $J$ = 7.5 Hz, 2H, $H_c$), 4.33 (m, 2H, $H_f$), 4.19 (m, 1H, $H_e$), 4.15 (m, 1H, $H_h$), 2.22 – 2.09 (m, 2H, $H_m$), 1.96 –1.67 (m, 11H, $H_m$ and $H_i$), 1.66 – 1.47 (m, 4H, $H_j$ and $H_k$). $^{13}C$ NMR (101 MHz, $CD_3OD$) δ: 174.2, 157.3, 143.9, 141.1, 127.4, 126.7, 124.8, 119.5, 66.5, 53.5, 46.9, 30.4, 26.5 (d, $J_{C\text{-}P}$ = 16.5 Hz), 22.5 (d, $J_{C\text{-}P}$ = 53.0 Hz), 20.5 (d, $J_{C\text{-}P}$ = 4.0 Hz), 6.3 (d, $J_{C\text{-}P}$ = 55.0 Hz). $^{31}P$ NMR (121 MHz, $CD_3OD$) δ: 27.25 (m, 1P). HR ESI-MS: 428.1985 ($[M]^+$) $C_{24}H_{31}NO_4P$, calc. 428.1985.

# 3 Supplementary References


1. Wang, G. G. *et al.* Haematopoietic malignancies caused by dysregulation of a chromatin-binding PHD finger. *Nature* **459**, 847–851 (2009).
2. Li, H. *et al.* Molecular basis for site-specific read-out of histone H3K4me3 by the BPTF PHD finger of NURF. *Nature* **442**, 91–95 (2006).
3. Zhao, S. *et al.* Kinetic and high-throughput profiling of epigenetic interactions by 3D-carbene chip-based surface plasmon resonance imaging technology. *Proc. Natl. Acad. Sci. U. S. A.* **114**, E7245–E7254 (2017).
4. Vermeulen, M. *et al.* Selective anchoring of TFIID to nucleosomes by trimethylation of histone H3 lysine 4. *Cell* **131**, 58–69 (2007).
5. Bian, C. *et al.* Sgf29 binds histone H3K4me2/3 and is required for SAGA complex recruitment and histone H3 acetylation. *EMBO J.* **30**, 2829–2842 (2011).
6. Lee, J., Thompson, J. R., Botuyan, M. V. & Mer, G. Distinct binding modes specify the recognition of methylated histones H3K4 and H4K20 by JMJD2A-tudor. *Nat. Struct. Mol. Biol.* **15**, 109–111 (2008).
7. Huang, Y., Fang, J., Bedford, M. T., Zhang, Y. & Xu, R. M. Recognition of histone H3 lysine-4 methylation by the double tudor domain of JMJD2A. *Science* **312**, 748–751 (2006).
8. Belle, R. *et al.* Investigating D-lysine stereochemistry for epigenetic methylation, demethylation and recognition. *Chem. Commun.* **53**, 13264–13267 (2017).
9. Kamps, J. J. A. G. *et al.* Chemical basis for the recognition of trimethyllysine by epigenetic reader proteins. *Nat. Commun.* **6**, 8911 (2015).
10. Al Temimi, A. H. K. *et al.* Recognition of shorter and longer trimethyllysine analogues by epigenetic reader proteins. *Chem. Commun.* **54**, 2409–2412 (2018).
11. Hintzen, J. C. J. *et al.* Comparison of molecular recognition of trimethyllysine and trimethylthialysine by epigenetic reader proteins. *Molecules* **25**, 1918 (2020).
12. Rahman, S. *et al.* Methyl-Induced Polarization Destabilizes the Noncovalent Interactions of N-Methylated Lysines. *Chem. Eur. J.* **27**, 11005–11014 (2021).
13. Chalker, J. M. *et al.* Methods for converting cysteine to dehydroalanine on peptides and proteins. *Chem. Sci.* **2**, 1666–1676 (2011).
14. Strazzolini, P., Melloni, T. & Giumanini, A. G. Selective nitrolytic deprotection of N-BOC-amines and N-BOC-amino acids derivatives. *Tetrahedron* **57**, 9033–9043 (2001).
15. Fekner, T., Li, X., Lee, M. M. & Chan, M. K. A pyrrolysine analogue for protein click chemistry. *Angew. Chem. Int. Ed.* **48**, 1633–1635 (2009).
16. Pedatella, S. *et al.* Antitumor agents 7. Synthesis, antiproliferative activity and molecular modeling of new L-lysine-conjugated pyridophenoxazinones as potent DNA-binding ligands and topoisomerase IIα inhibitors. *Eur. J. Med. Chem.* **187**, 111960 (2020).
17. Bambal, R. & Hanzlik, R. P. Synthesis of Nϵ(p-Bromophenyl)-L-lysine and Nτ-(p-Bromophenyl)-L-histidine as Models for Adducts of Bromobenzene 3,4-Oxide to Protein. Observation of an Unusual Pd-Catalyzed Nτ - to Nπ-Aryl Substituent Migration. *J. Org. Chem.* **59**, 729–732 (1994).
18. Yokokawa, F. *et al.* An expeditious synthesis of pentosidine, an advanced glycation end product. *Tetrahedron* **57**, 4759–4766 (2001).
19. Jeon, S. *et al.* Synthesis of photoresponsive dual NIR two-photon absorptive [60]fullerene triads and tetrads. *Molecules* **18**, 9603–9622 (2013).

# 4 Spectral data for synthesised compounds

***(S)-tert*-Butyl 6-(((benzyloxy)carbonyl)amino)-2-((*tert*-butoxycarbonyl)amino)hexanoate 2**

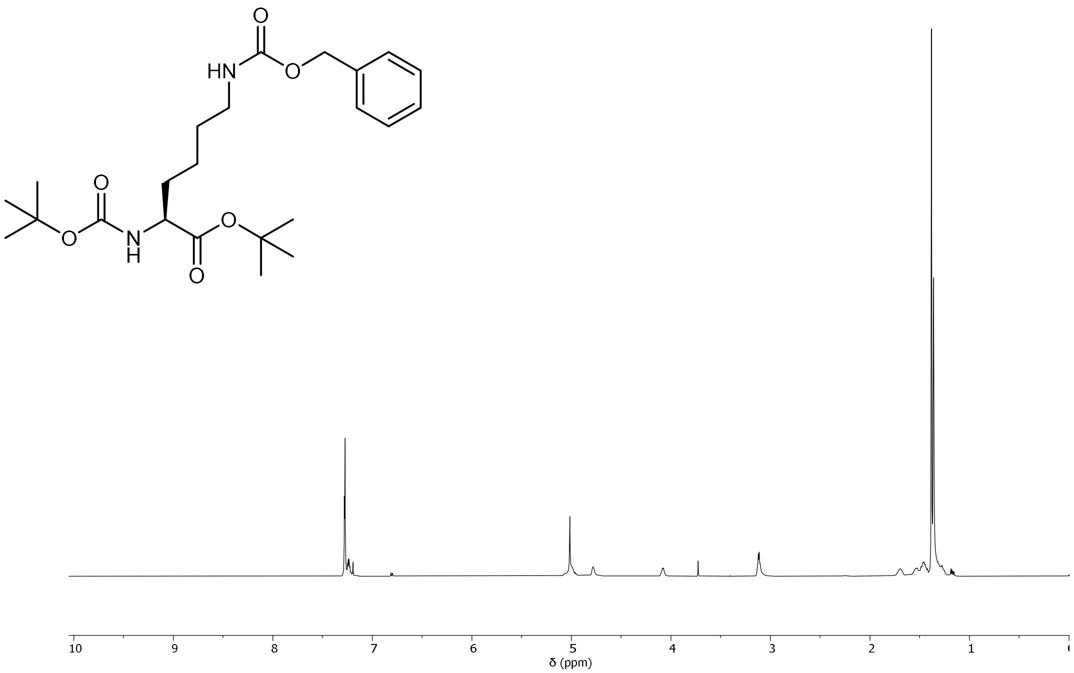


**Supplementary Fig. 25** $^{1}$H NMR (600 MHz) spectrum of **2** in $CDCl_3$.

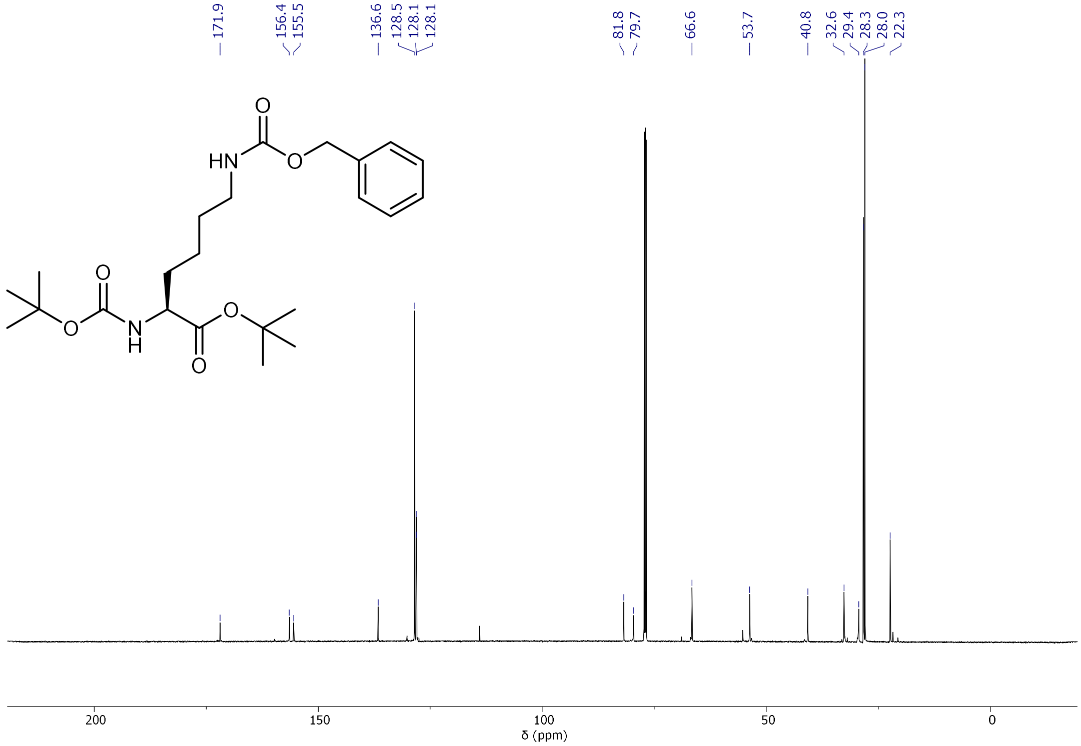


**Supplementary Fig. 26** $^{13}$C NMR (151 MHz) spectrum of **2** in $CDCl_3$.

***(S)-tert*-Butyl 6-amino-2-((*tert*-butoxycarbonyl)amino)hexanoate 3**

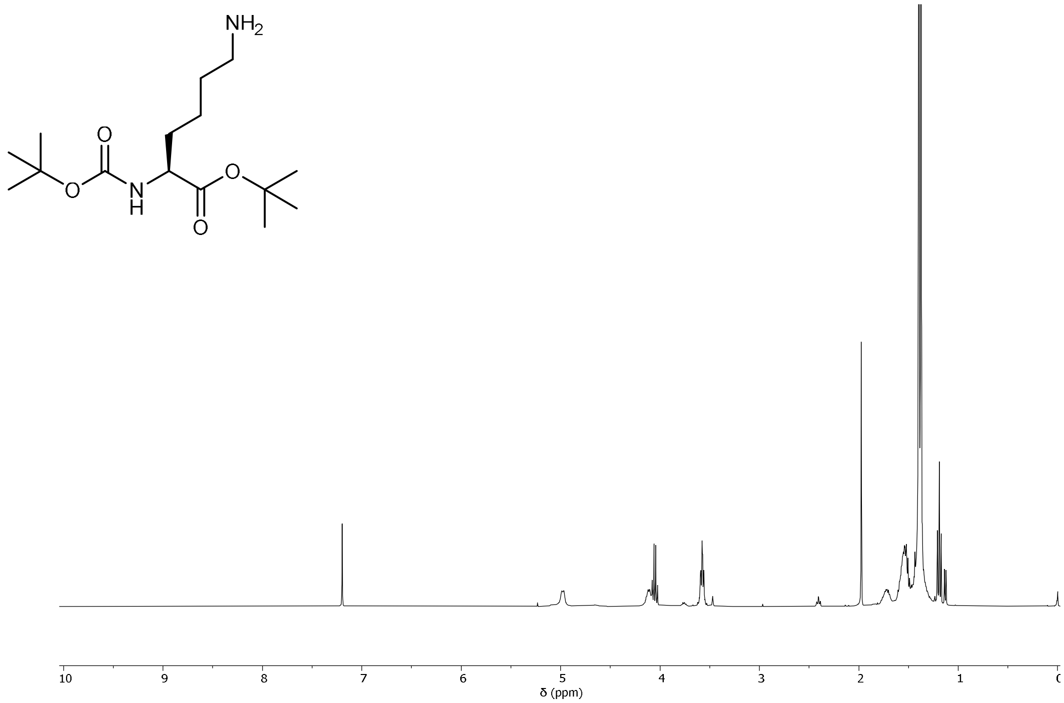


**Supplementary Fig. 27** $^{1}$H NMR (400 MHz) spectrum of **3** in $CDCl_3$.

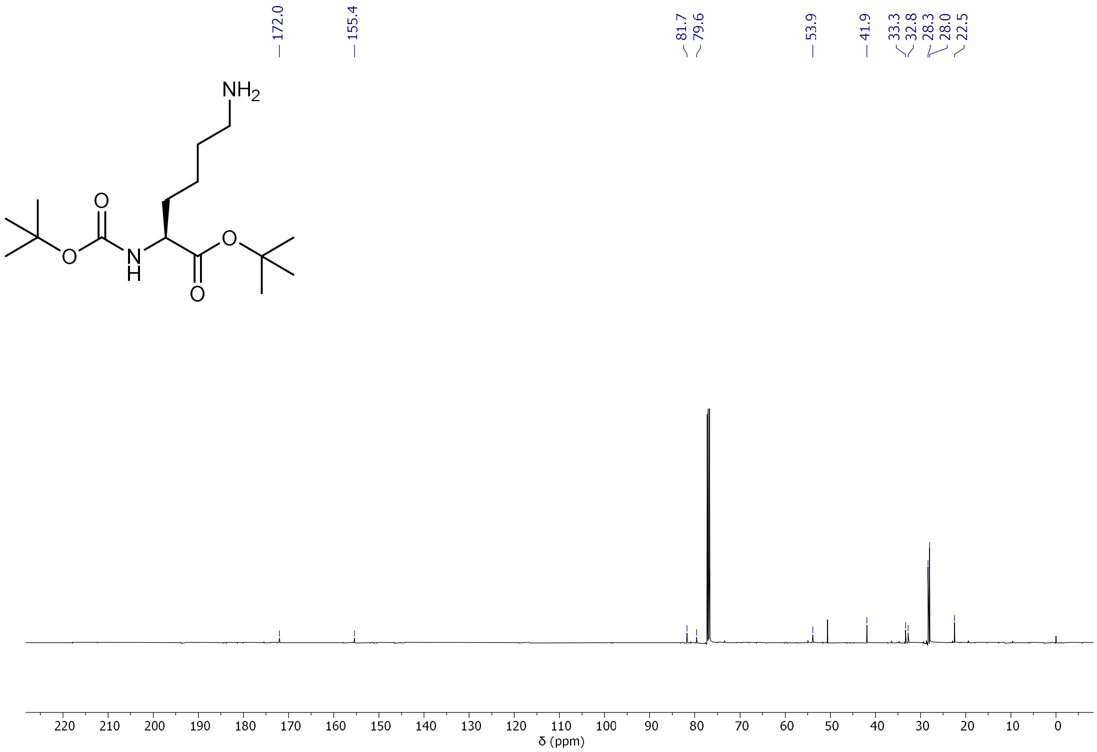


**Supplementary Fig. 28** $^{13}$C NMR (126 MHz) spectrum of **2** in $CDCl_3$.

***(S)-tert*-Butyl 2-((*tert*-butoxycarbonyl)amino)-6-hydroxyhexanoate 4**

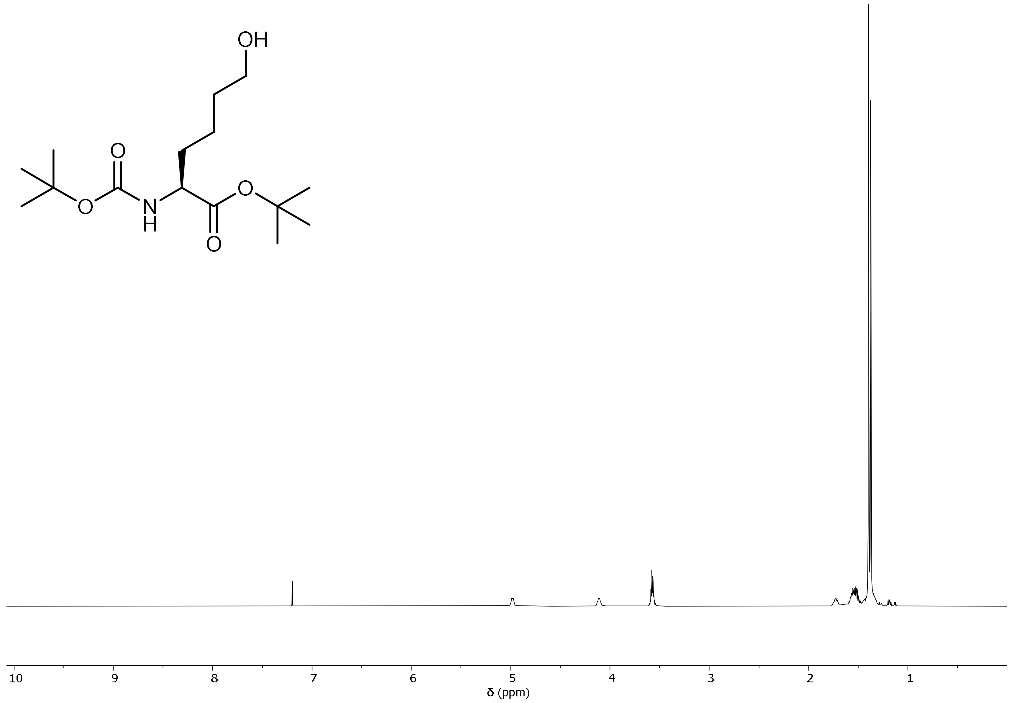


**Supplementary Fig. 29** $^{1}H$ NMR (600 MHz) spectrum of **4** in $CDCl_3$.

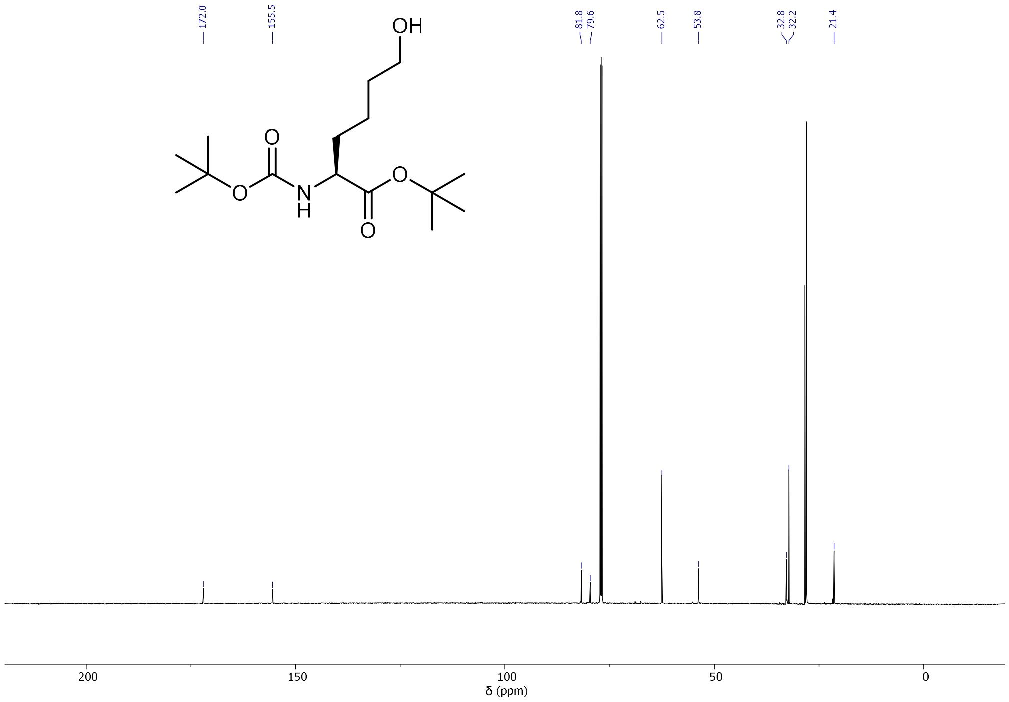


**Supplementary Fig. 30** $^{13}C$ NMR (151 MHz) spectrum of **4** in $CDCl_3$.

***(S)-tert*-Butyl 2-((tert-butoxycarbonyl)amino)-6-((methylsulfonyl)oxy)hexanoate 5**

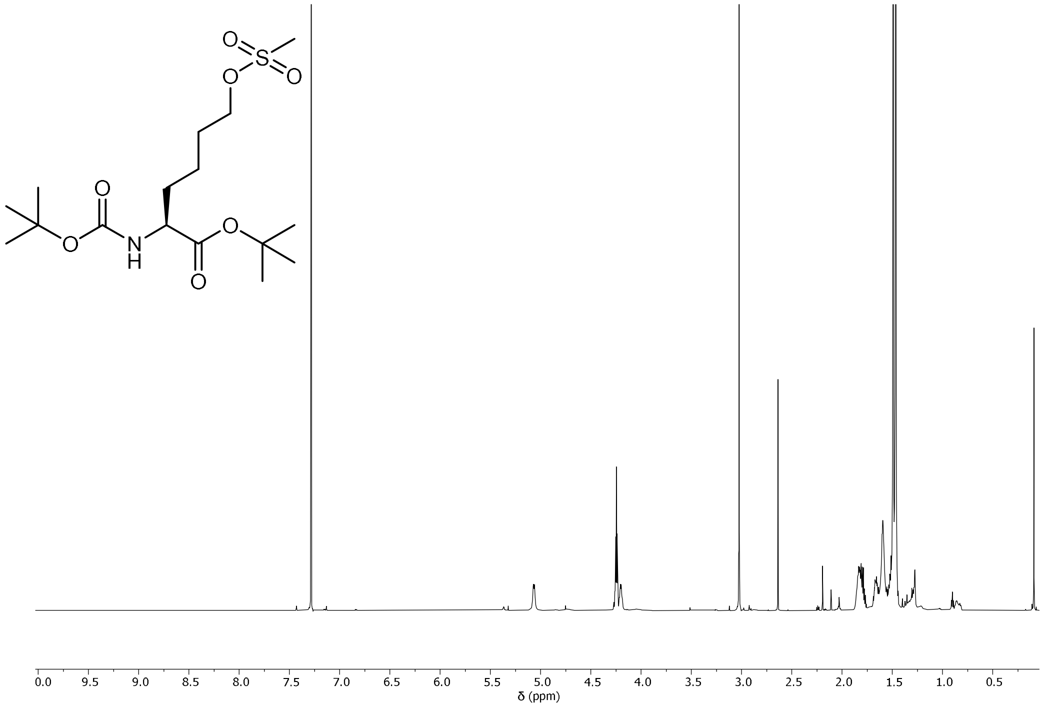


**Supplementary Fig. 31** $^{1}$H NMR (400 MHz) spectrum of **5** in $CDCl_3$.

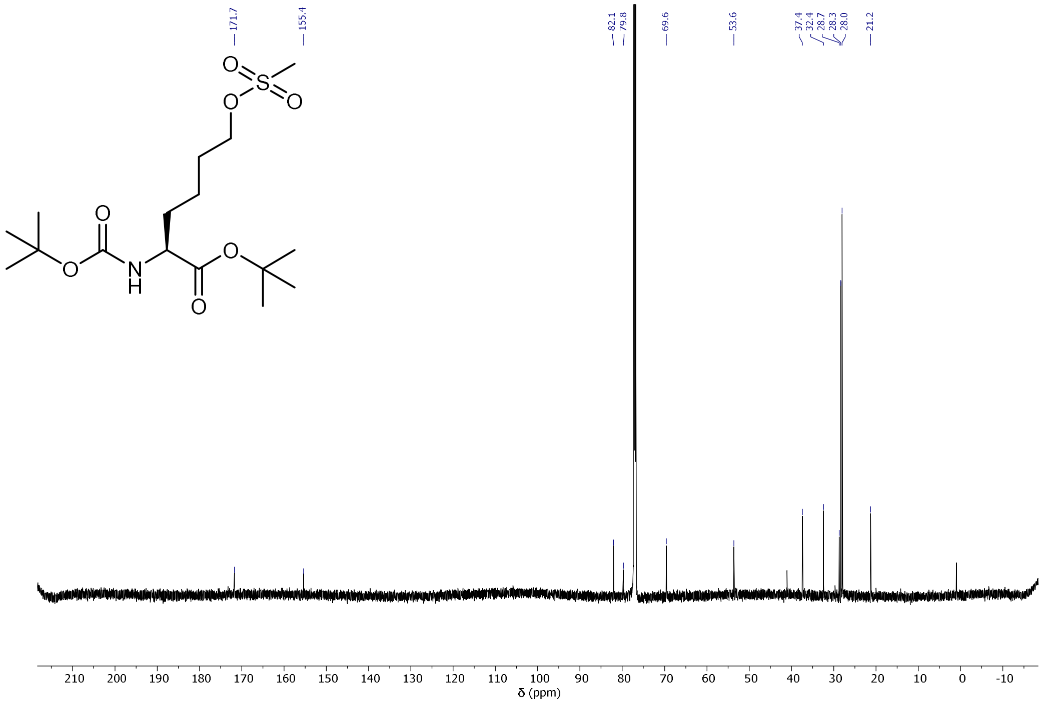


**Supplementary Fig. 32** $^{13}$C NMR (101 MHz) spectrum of **5** in $CDCl_3$.

***(S)*-(6-(*tert*-Butoxy)-5-((*tert*-butoxycarbonyl)amino)-6-oxohexyl)trimethylphosphonium methansulfonate salt 6**

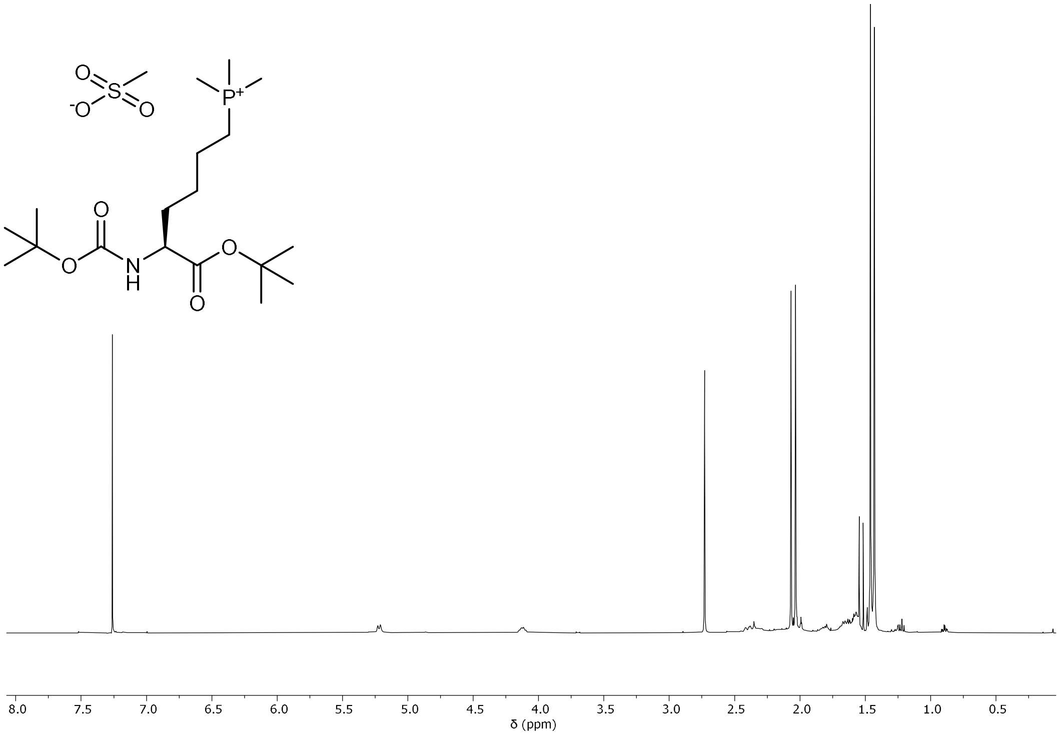


**Supplementary Fig. 33** $^{1}$H NMR (400 MHz) spectrum of **6** in $CDCl_3$.

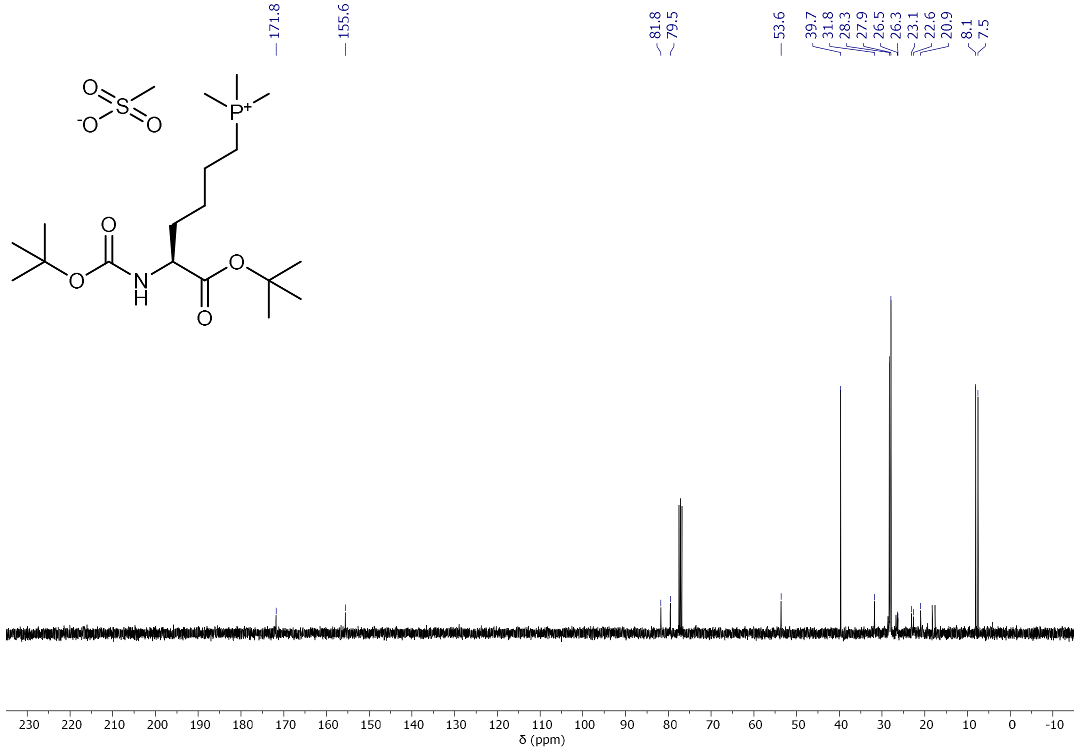


**Supplementary Fig. 34** $^{13}$C NMR (101 MHz) spectrum of **6** in $CDCl_3$.

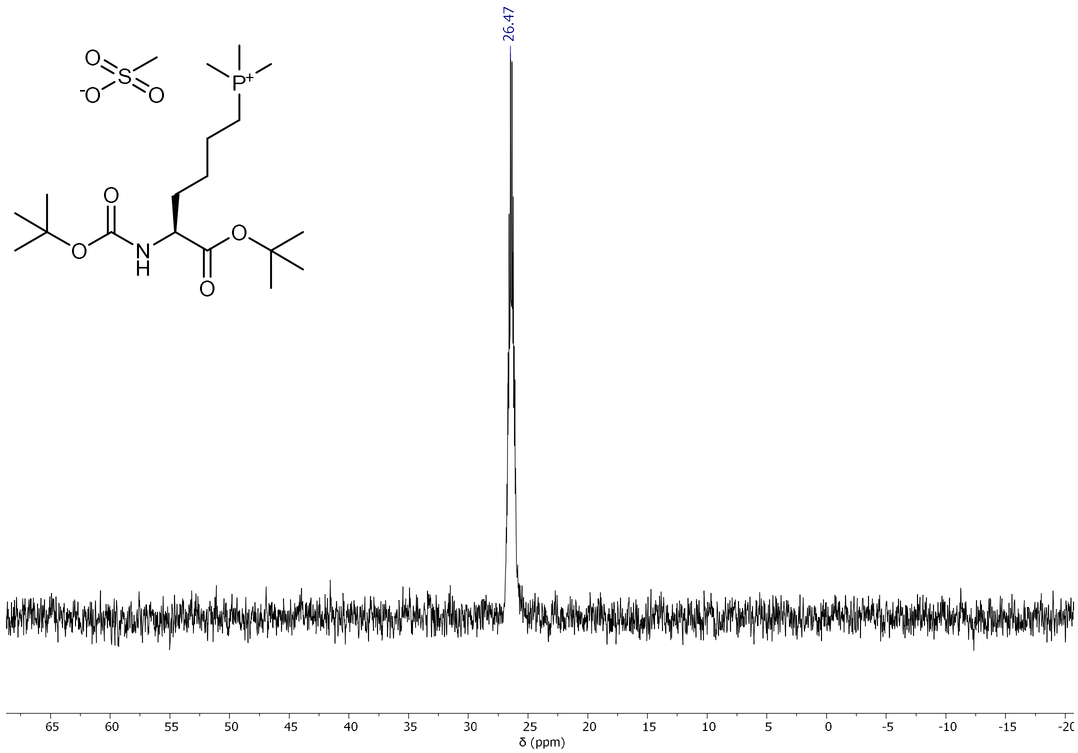


**Supplementary Fig. 35** $^{31}P$ NMR (121 MHz) spectrum of **6** in $CDCl_3$.

***(S)*-(5-((((9*H*-Fluoren-9-yl)methoxy)carbonyl)amino)-6-(trimethylphosphonio)hexanoate 7**

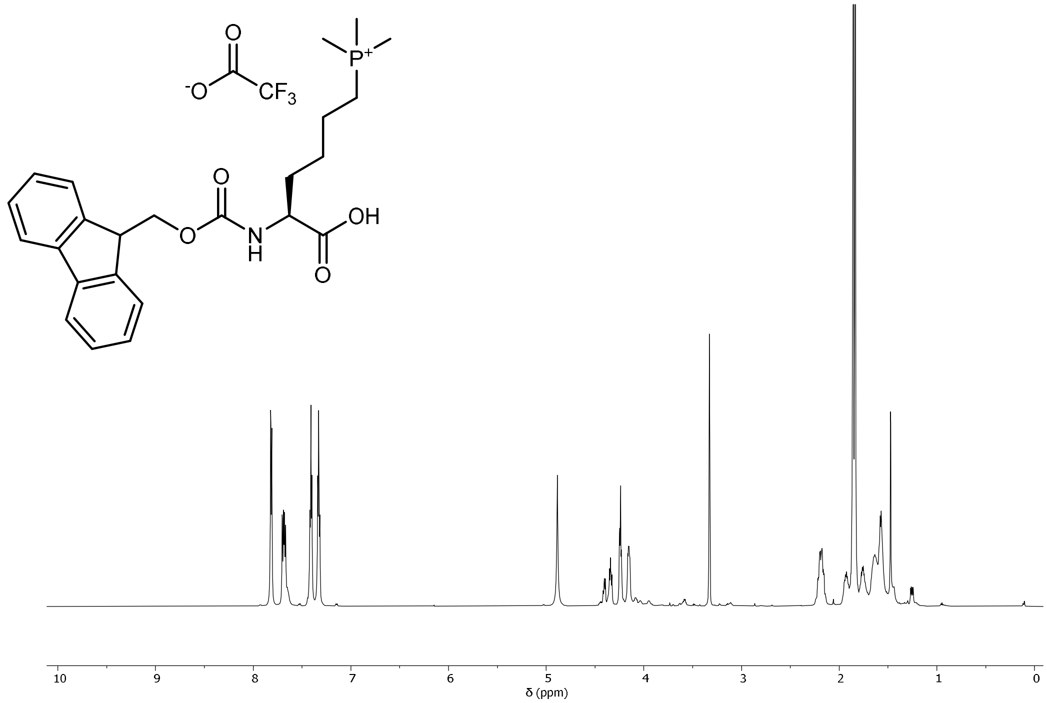


**Supplementary Fig. 36** $^{1}$H NMR (400 MHz) spectrum of the Fmoc-K$_{P}$me$_{3}$-OH TFA salt **7** in $CD_3OD$.

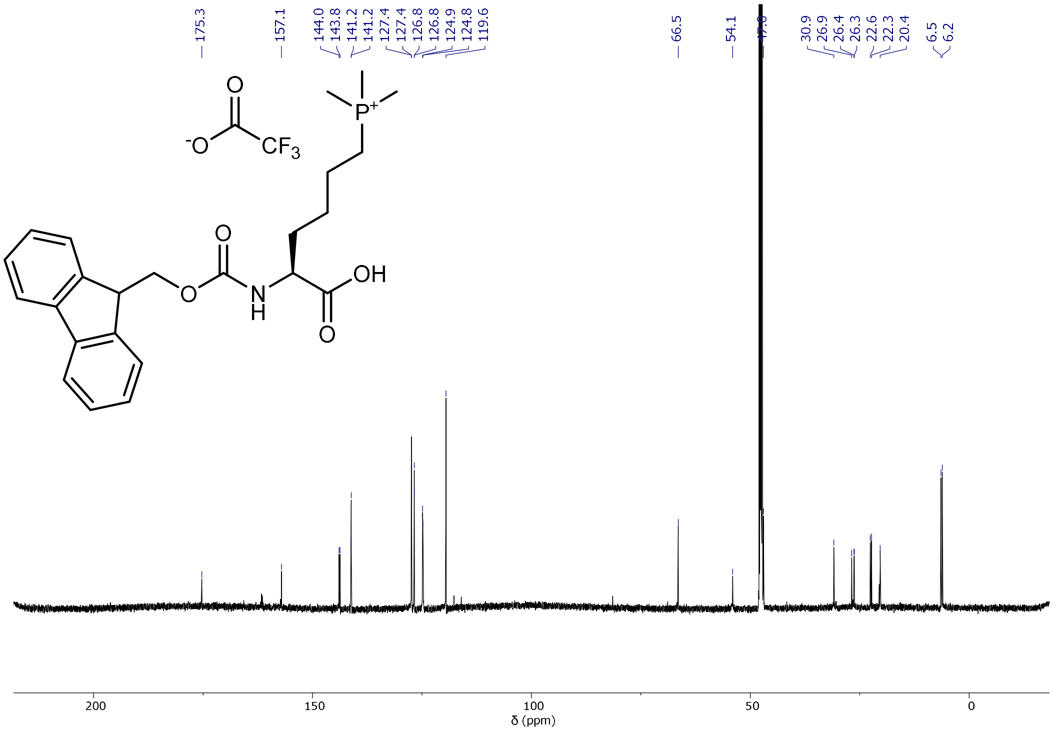


**Supplementary Fig. 37** $^{13}$C NMR (101 MHz) spectrum of Fmoc-K$_{P}$me$_{3}$-OH TFA salt **7** in $CD_3OD$.

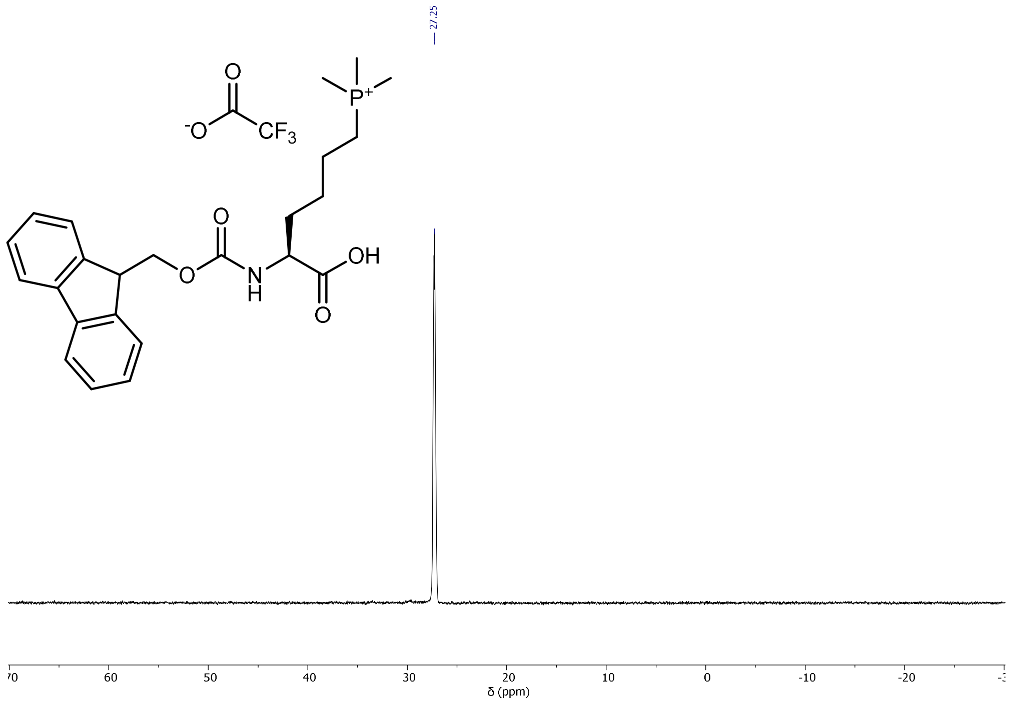


**Supplementary Fig. 38** $^{31}$P NMR (121 MHz) spectrum of Fmoc-K$_P$me$_3$-OH TFA salt **7** in $CD_3OD$.
